# Combined Chromatin and Expression Analysis Reveals Specific Regulatory Mechanisms within Cytokine Genes in the Macrophage Early Immune Response

**DOI:** 10.1371/journal.pone.0032306

**Published:** 2012-02-27

**Authors:** Maria Jesus Iglesias, Sarah-Jayne Reilly, Olof Emanuelsson, Bengt Sennblad, Mohammad Pirmoradian Najafabadi, Lasse Folkersen, Anders Mälarstig, Jens Lagergren, Per Eriksson, Anders Hamsten, Jacob Odeberg

**Affiliations:** 1 Atherosclerosis Research Unit, Department of Medicine, Centre for Molecular Medicine, Karolinska Institute, Stockholm, Sweden; 2 Science for Life Laboratory, Division of Gene Technology, School of Biotechnology, KTH Royal Institute of Technology, Stockholm, Sweden; 3 Department of Computational Biology, KTH Royal Institute of Technology, Stockholm, Sweden; 4 Department of Cardiology, Karolinska University Hospital, Solna, Stockholm, Sweden; 5 Centre for Hematology, Karolinska University Hospital Solna, Stockholm, Sweden; 6 Science for Life Laboratory, Division of Proteomics, School of Biotechnology, KTH Royal Institute of Technology, Stockholm, Sweden; University of Crete, Greece

## Abstract

Macrophages play a critical role in innate immunity, and the expression of early response genes orchestrate much of the initial response of the immune system. Macrophages undergo extensive transcriptional reprogramming in response to inflammatory stimuli such as Lipopolysaccharide (LPS).

To identify gene transcription regulation patterns involved in early innate immune responses, we used two genome-wide approaches - gene expression profiling and chromatin immunoprecipitation-sequencing (ChIP-seq) analysis. We examined the effect of 2 hrs LPS stimulation on early gene expression and its relation to chromatin remodeling (H3 acetylation; H3Ac) and promoter binding of Sp1 and RNA polymerase II phosphorylated at serine 5 (S5P RNAPII), which is a marker for transcriptional initiation. Our results indicate novel and alternative gene regulatory mechanisms for certain proinflammatory genes. We identified two groups of up-regulated inflammatory genes with respect to chromatin modification and promoter features. One group, including highly up-regulated genes such as tumor necrosis factor (*TNF*), was characterized by H3Ac, high CpG content and lack of TATA boxes. The second group, containing inflammatory mediators (interleukins and CCL chemokines), was up-regulated upon LPS stimulation despite lacking H3Ac in their annotated promoters, which were low in CpG content but did contain TATA boxes. Genome-wide analysis showed that few H3Ac peaks were unique to either +/−LPS condition. However, within these, an unpacking/expansion of already existing H3Ac peaks was observed upon LPS stimulation. In contrast, a significant proportion of S5P RNAPII peaks (approx 40%) was unique to either condition. Furthermore, data indicated a large portion of previously unannotated TSSs, particularly in LPS-stimulated macrophages, where only 28% of unique S5P RNAPII peaks overlap annotated promoters. The regulation of the inflammatory response appears to occur in a very specific manner at the chromatin level for specific genes and this study highlights the level of fine-tuning that occurs in the immune response.

## Introduction

Macrophages play a critical role in innate immunity, and recognize ‘microbial non-self’ via receptors known as toll-like receptors (TLRs) that recognize pathogen-associated molecular patterns (PAMPs) [1]. Macrophages display very different transcriptional profiles during different conditions, and in particular there are major changes in transcriptional output in response to inflammatory stimuli, such as lipopolysaccharide (LPS). It has been shown, using expression profiling, that a number of macrophage genes are induced or repressed in response to LPS [2], and this response has been described as an activation process that is controlled by a dynamically inducible transcriptional regulatory network [3]. This process is important, especially for the early response genes, including those encoding proinflammatory cytokines and chemokines, since many of these only undergo a transient induction that peaks between 2–7 hours [2]. Cytokines are small proteins expressed by cells of the immune system, and control both physiological and pathological conditions. There is strong linkage between the expression and secretion of cytokines, and pathological conditions in which inflammation plays an important role, such as that seen in the development and progression of coronary artery disease [4].

The expression and repression of genes is associated with alterations in chromatin structure mediated by enzymatic modifications (acetylation, methylation and phosphorylation) of the N-terminal ends of the core histones in nucleosomes [5], [6]. Histone acetylation is associated with active promoters and open chromatin [7], and is important for transcriptional regulation because it can be added or removed in response to the state of the cell [8]. It has previously been shown that LPS-stimulated macrophages undergo extensive transcriptional reprogramming that is mediated in part by changes in histone acetylation [2]. Acetylation of histone 3 (H3Ac) at lysine residues (K9 and K14) is one of the more widely studied chromatin modifications. It has been shown to have a functional impact on many aspects of chromatin accessibility for DNA binding proteins, in particular within promoter regions, and may determine the transcriptional regulatory state of a given gene [9].

The recruitment of the RNA polymerase II (RNAPII) in the core promoter is essential for gene expression. However, there is not necessarily a correlation between the rate of its recruitment and mRNA levels of a gene [10]. RNAPII bound at the core promoter is functionally regulated through dynamic changes of the serine phosphorylation status at the C-terminal domain YSPTSPS (CTD). Different degrees of phosphorylation at the CTD are associated with different stages of gene transcription (preinitiation, initiation, and elongation) [11], [12]. The RNAPII CTD is thought to be hypophosphorylated in the preinitiation complex; which is poised in the core promoter at the active transcription start site (TSS) and becomes increasingly phosphorylated at initiation of transcription [11]–[13]. Serine 5 phosphorylation of the RNAPII (S5P RNAPII), which occurs in the preinitiation stage, has been found to be concentrated near active promoters, and is sufficient to recruit and stimulate capping enzymes to the RNAPII initiation complex prior to elongation [12].

Studies of gene regulation have advanced rapidly in recent years, and this is due to the development of next generation sequencing techniques. Chromatin immunoprecipitation followed by massively parallel sequencing (ChIP-seq) has become a valuable experimental tool for examining protein-DNA interactions [14], and allows identification of promoter regions over the whole genome. We have used two genome-wide approaches, ChIP-seq and gene expression profiling to examine how LPS stimulation affects chromatin remodeling and S5P RNAPII and the transcription factor (TF) Sp1 binding at gene promoters in macrophages, and to see whether these modifications have a down-stream effect on gene expression. Our results identify alternative regulatory mechanisms of gene expression in innate immunity of some proinflammatory cytokines. Improved knowledge about changes in histone modifications, such as acetylation, in these genes will provide better understanding of the molecular mechanisms behind various chronic inflammatory diseases. Additionally, our results indicate a significant number of novel previously unannotated TSSs in genes affected by the inflammatory stimuli.

## Materials and Methods

### THP-1 cell culture and differentiation

THP-1 cells (monocyte cell line) (ATCC, Manassas, VA, USA) were maintained in culture in RPMI 1640 medium containing 10% fetal bovine serum, 1 mM sodium pyruvate, 100 units/ml penicillin and 100 µg/ml streptomycin at 37°C with 5% CO_2_. For differentiation of THP-1 cells into macrophages a protocol using conditioned media as previously described by Whatling et al [15] was used. To confirm differentiation of THP-1 monocytes into macrophages, *CD11b* expression was measured using FACS (Text S1) and quantitative polymerase chain reaction (qPCR) (Text S1).

THP-1 macrophages were then either left unstimulated (−LPS) or stimulated with 1 µg/ml LPS (+LPS) from *Escherichia coli* O55:B5 for 2 hours (Sigma Chemical Co., St. Louis, MO, USA) to induce an acute inflammatory response. To confirm an induction of the inflammatory response in the THP-1 macrophages, *TNF* mRNA levels were measured in +/−LPS stimulated samples (Text S1).

### mRNA extraction and microarray gene expression analysis

THP-1 macrophages (0.5×10^6^) were incubated for 2 hours (+/−LPS, n = 4) and total mRNA was extracted using TRIZOL® reagent (Invitrogen, Carlsbad, CA, USA) and purified using the RNeasy Mini Kit following the manufacturer's instructions, including DNase treatment (Qiagen, Valencia, CA, USA). RNA concentration and quality were analyzed using Nanodrop 1000 Spectrophotometer (NanoDrop Technologies, Wilmington, DE, USA) and Agilent 2100 Bioanalyzer (Agilent Technologies, Inc., Santa Clara, CA, USA) respectively. The RNA was then hybridized and scanned on the Affymetrix® Genechip® Human Exon 1.0 ST array (Affymetrix, Santa Clara, CA, USA) following manufacturer's protocols at the Karolinska Institute microarray core facility. Standard Affymetrix quality controls were followed (Text S1). The subset of genes with a ≥+/−2 fold change in expression and P<0.05 (Student's t-test two-sided) between +/−LPS stimulated samples were defined as regulated. Gene Ontology (GO) analysis was performed on the significant up- and down regulated genes using GENOMATIX software v.2 (http://www.genomatix.de).

### Chromatin immunoprecipitation

Chromatin immunoprecipitation (ChIP) was performed on differentiated THP1 macrophage cells +/−LPS stimulation for 2 hours using a method previously described by Rada-Iglesias et al [16]. Immunoprecipitation was performed with specific antibodies raised against acetylated groups (K9 and K14) of histone 3 (H3Ac) (Upstate, Charlottesville, VA, USA; ref 06-599), phosphorylated serine at the 5 position of the C- terminal domain repeat (YSPTSPS) of RNA polymerase II (Abcam Cambridge, UK; ref ab5131) or Sp1 transcription factor (Upstate, Charlottesville, VA, USA; ref 17-601). Anti-rabbit IgG antibody (Abcam Cambridge, UK) was used as a negative control. DNA-protein complexes were eluted, treated with RNase (USB, Cleveland, OH, USA) for 4–6 hours at 45°C and Proteinase K (USB, Cleveland, OH, USA) overnight at 65°C. DNA was extracted by phenol/chloroform/isoamyl alcohol extraction, purified and resuspended in water. Immunoprecipitated DNA was validated using qPCR (Text S1). ChIP experiments were performed independently and in duplicate for sequencing.

### ChIP-sequencing and peak calling

Sequencing was performed on the Illumina Genome Analyzer I (SNP&SEQ technology platform, Uppsala University, Sweden). The data is available from gene expression omnibus under accession number GSE32325. All reads that passed mapping quality checks (Text S1) were analyzed using SICER [17], a clustering approach for identification of enriched domains from histone modification ChIP-seq data or a model-based analysis of ChIP-seq (MACS) for transcription factor binding [18] to predict enriched islands (peaks) (Text S1). Galaxy, an online data analysis platform (http://main.g2.bx.psu.edu/) [19] was used to determine significant and replicated peaks between the biological replicas.

### *In silico* TATA box searching

TATA-box patterns were identified in the promoter sequences of genes downloaded from the UCSC browser. Using GENOMATIX, classical TATA boxes matrices were used for *in silico* analysis and a matrix similarity value of 0.9 was used as the cut-off point.

### Integrated analysis

Using the genes identified in the microarray gene expression analysis, a list was generated of those that had peaks that were replicated in the biological replicas. Data for CpG island coordinates were downloaded from the UCSC genome browser [20]. Integration was performed by examining the promoter regions of the genes identified, and looking for the presence or absence of (i) H3Ac, (ii) S5P RNAPII, (iii) Sp1, (iv) CpG islands and (v) TATA box.

## Results

### Genes involved in the inflammatory response dominate amongst the up-regulated genes

The differentiation of THP-1 monocytes into THP-1 macrophages was confirmed by microscopy [Figure S1A] and measurement of *CD11b* expression using FACS [Figure S1B] and qPCR (data not shown). A genome-wide analysis of changes in gene expression between differentiated THP-1 cells after 2 hours of stimulation with LPS or control cells was performed. Using the described expression array criteria (≥+/−2 fold change and P<0.05), we identified 181 up-regulated genes and 26 down-regulated genes (for a complete list of up and down-regulated genes see Table S1). The genes showing the highest up-regulation, with a 20-fold induction or more after two hours of LPS stimulation were, *TNF*, *CCL4, IL1A*, *PTGS2*, *CXCL2* and *NFkBIZ*. Gene Ontology (GO) analysis was used to establish the functional and biological roles of these identified genes and the major GO-terms identified for the up-regulated genes were related to the immune system, inflammatory response, apoptosis, and cell death [Table S2].

### The majority of Histone 3 acetylation sites are preserved after LPS stimulation

ChIP-seq was used to study changes in macrophage chromatin state during the early inflammatory response and to identify genomic regions with active chromatin, which we defined as the acetylation of histone 3 (H3Ac) [21], [22]. ChIP-seq analysis for H3Ac was performed for two biological replicates each for 2 hours +/−LPS-stimulated cells, and the results showed high sample reproducibility with a similar number of significant peaks (false discovery rate (FDR) ≤0.001) found in each replicate [Table S3].

A major observation was that only a small proportion of H3Ac peaks were unique to either unstimulated or LPS-stimulated macrophages. 2,272 H3Ac peaks disappeared upon LPS stimulation and only 217 novel peaks appeared; and we refer to these as unique peaks [Table 1]. Instead, a large proportion of the identified peaks (11,834 peaks) overlapped in their genomic location before and after stimulation; these are called common H3Ac peaks [Table 1].

**Table 1:** Summary of H3Ac and S5P RNAPII ChIP-seq peaks identified and their locations.

	H3AC ChIP-Seq Results				
	−LPS	+LPS	+/−LPS Common peaks	−LPS Unique peaks	+LPS Unique peaks
Total number of H3Ac peaks	14,704 (3,552 bp)	12,113 (6,016 bp)	11,834 (n/a)	2,272 (2,569 bp)	217 (5,936 bp)
% of peaks located in promoter	87.9%	93.8%	94.4%	61.2%	66.8%
High CpG island content peaks	12,911 (3,588 bp)	11,339 (6,016 bp)	11,161 (n/a)	1,365 (2,459 bp)	134 (5,933 bp)
% Located in promoter	91.8%	94.8%	95.1%	71.6%	70.1%
Low CpG island content peaks	1,793 (3,287 bp)	774 (6,007 bp)	673 (n/a)	907 (2,734 bp)	83 (5,941 bp)
% Located in promoter	60%	79.2%	82.0%	45.6%	61.4%

	S5P RNAP II ChIP-Seq Results				
	−LPS	+LPS	+/−LPS Common peaks	−LPS Unique peaks	+LPS Unique peaks
Total number of S5P RNAPII peaks	10,000 (941 bp)	9,220 (1,018 bp)	5,629 (n/a)	4,371 (769 bp)	3,591 (741 bp)
% of peaks located in promoter	74.2%	66.6%	91.5%	51.9%	27.8%
High CpG island content peaks	7,212 (1,067 bp)	5,880 (1,195 bp)	5,083 (n/a)	2,129 (973 bp)	802 (1,021 bp)
% Located in promoter	94.2%	95.1%	96.3%	89.1%	88%
Low CpG island content peaks	2,788 (616 bp)	3,340 (705 bp)	546 (n/a)	2,242 (574 bp)	2,803 (661 bp)
% Located in promoter	22.6%	16.5%	47.1%	16.6%	10.5%

Average peak length showed in brackets for each group of peaks. n/a, not applicable; the common peaks represent those that are present in both −LPS total and +LPS total, and the average length of these are shown in the −LPS and +LPS peaks columns.

### H3Ac peaks are significantly widened in LPS-stimulated cells

**Figure 1:**
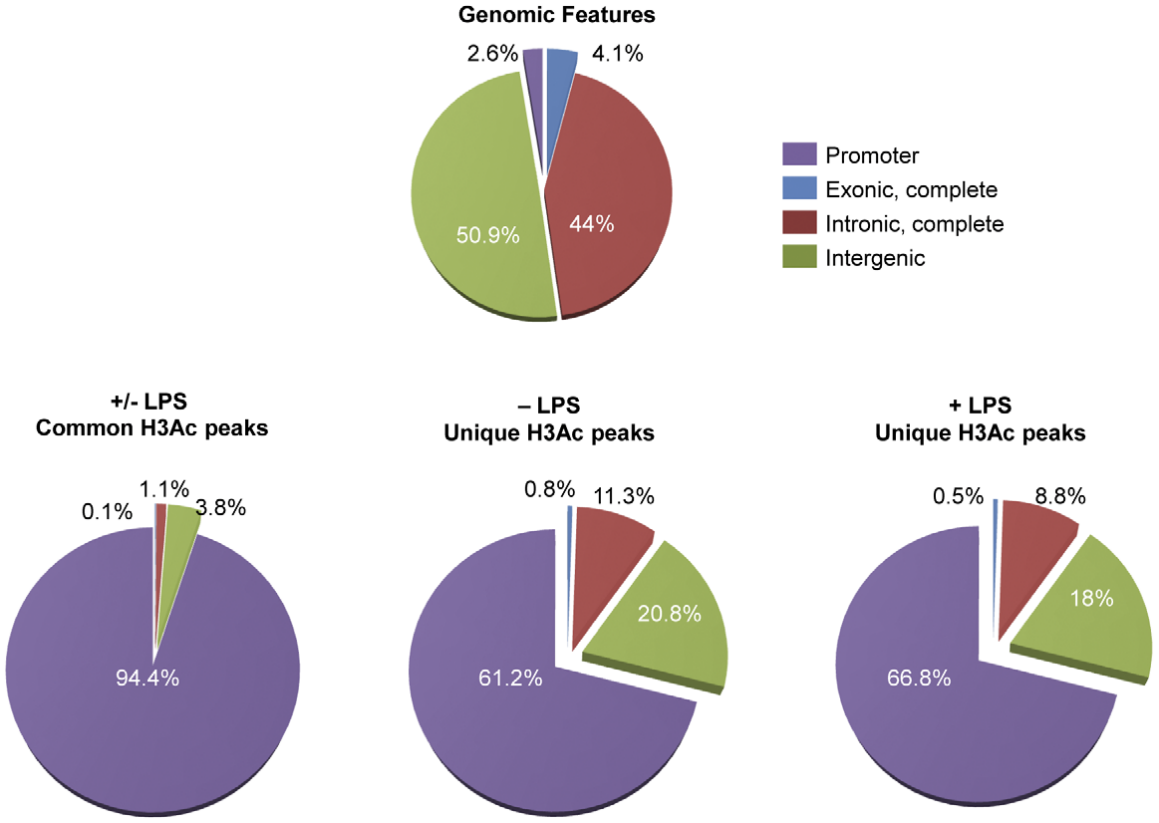
Distribution of H3Ac peaks across the macrophage genome. Graph (A) shows a summary of the expected distribution of genomic features of the whole human genome (2.9 billion base pairs). Graph (B) shows the location of common H3Ac peaks from the macrophage genome. (C) & (D) show the location of unique H3Ac peaks in macrophages during the experimental conditions, unstimulated (−LPS) and LPS-stimulated (+LPS), respectively.

The average lengths of the acetylated peaks detected in macrophages were approximately doubled upon LPS stimulation [Table 1]. Data indicated that an additional chromatin unpacking occurs in genomic regions that already have accessible chromatin [Figure S2]. Over 90% of the common peaks overlapped a known promoter region of a gene, and very few were found in exons, introns or intergenic regions [Table 1 & Figure 1]. Furthermore, the common peaks showed a high level of co-localization with CpG islands (94%; 11,161 out of 11,834) [Table 1].

Unique H3Ac peaks were found to have less overlap with promoters and CpG islands compared to common peaks. Only a 66.8% (+LPS) and 61.2% (−LPS) of the unique peaks mapped to a known promoter region, and the remainder were mostly located in intron and intergenic regions [Figure 1] The unique H3Ac peaks in LPS-stimulated cells were found on average to be wider than those identified in the unstimulated cells [Table 1]. Furthermore, the unique peaks did not show the same extent of co-localization with CpG islands as the common peaks (60% compared to 94%) [Table 1].

### H3Ac peaks specific to LPS stimulation can appear inside genes whose promoter already had open chromatin

**Figure 2:**
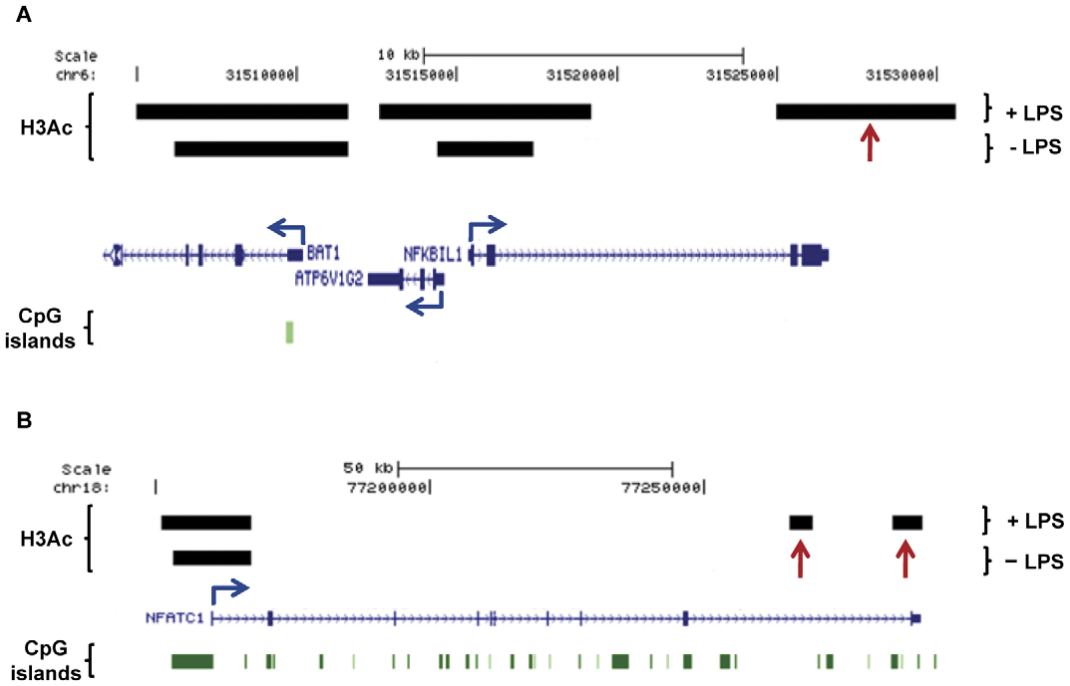
LPS treatment and the appearance of unique H3Ac peaks. *NFKBIL1* and *NFATC1* are examples of genes having common H3Ac peaks in both +/−LPS conditions. They also have unique H3Ac peaks that appear upon LPS stimulation (indicated with red arrows). (2A) A unique H3Ac peak appears at the 3′ end of *NFKBIL1* after LPS stimulation. (2B) For *NFATC1* two unique H3Ac peaks appear, these are located at the intronic and 3′end region, respectively. Genes identified by RefSeq using the UCSC browser are shown with blue arrows indicating TSSs locations and directions (GRCh37/hg19).

The LPS-induced intronic/exonic unique peaks not overlapping CpG islands were in many instances found in genes that have a common peak in their promoter region (35 peaks out of 45). This shows that novel acetylated regions appear inside genes that already have open chromatin in their promoters, indicating an unpacking of chromatin and an exposure of intragenic enhancer elements (this is shown for *NFkBIL1* and *NFATC1* in Figures 2A and 2B, respectively). However, in the gene expression analysis described below, no consistent pattern of up- or down-regulation among the subset of genes with this H3Ac pattern could be deduced.

### Significant alterations in S5P RNAPII binding occur upon LPS stimulation

**Figure 3:**
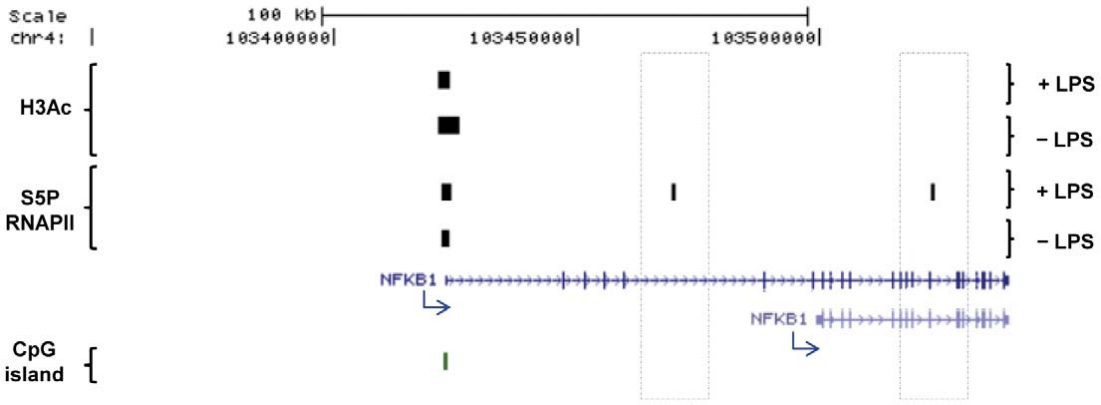
S5P RNAPII ChIP-seq peaks indicate novel alternative promoters and transcription start sites in macrophages upon LPS. *NFKB1* is an example of the identification of a previously unannotated promoter in macrophages after LPS. There is a S5P RNAPII signal for both conditions at the TSS of the large annotated transcript. This TSS also harbours acetylated peaks before and after LPS and high CpG content. Two new S5P RNAPII binding sites (highlighted with a line dots square) appear in response to LPS. These two LPS-induced S5P RNAPII peaks do not colocalize either with regions having H3Ac peaks or high CpG content. Genes identified by RefSeq using the UCSC browser are shown with blue arrows indicating TSSs locations and directions (GRCh37/hg19).

The localization of S5P RNAPII, characterized to be poised at the promoter region [11]–[13], was analyzed by ChIP-seq in macrophages after 2 hours of LPS stimulation. A total of 10,000 and 9,220 S5P RNAP II peaks were found in unstimulated and LPS-treated conditions, respectively [Table 1]. In contrast to the H3Ac patterns, there was significant non-overlap in the genomic locations of the S5P RNAPII peaks, where 5,629 peaks were common while 4,371 peaks disappeared and 3,591 appeared upon LPS stimulation. Of the common peaks, the majority (91.5%) co-localized with a known (annotated) promoter, while only 51.9% of the unique −LPS and 27.8% of the unique +LPS peaks did mapped to annotated promoters. This indicates the use of novel promoters and transcription factor start sites (TSSs) in genes with unique S5P RNAPII peaks [Table 1; Figure 3]. Furthermore, while common S5P RNAPII peaks colocalized to a high degree (91.7%) with H3Ac modified regions, unique peaks showed a lower degree of co-localization with acetylated histone 3 regions (53% and 20.3% for −LPS and +LPS unique peaks, respectively) [Table 2]. Approximately 85% of these +LPS unique promoters that do not overlap H3Ac peaks were not in previously annotated TSS/promoters [Table 2], indicating that for these novel promoters, H3Ac is not a prerequisite for assembling of the transcription complex/machinery. *In silico* TF search in unique S5P RNAPII peaks (either overlapping or not overlapping H3Ac regions) revealed a different pattern of overrepresented putative transcription binding elements in these two subgroups [Table S4]

**Table 2:** Summary of the combined analysis of S5P RNAPII and H3Ac ChIP-seq peaks and their locations.

S5P RNAPII ChIP-seq peaks co-localized with H3Ac ChIP-seq peaks					
	−LPS	+LPS	+/−LPS Common peaks	−LPS unique peaks	+LPS unique peaks
Number of peaks (% of total S5P RNAPII*)	7,510 (75.1%)	5,879 (63.7%)	5,162 (91.7%)	2,348 (53.%)	731 (20.3%)
% of peaks located in promoter	93.9%	95.8%	96.6%	88%	90.8%

S5P RNAPII ChIP-seq peaks NOT co-localized with H3Ac ChIP-seq peaks					
	−LPS	+LPS	+/−LPS Common peaks	−LPS unique peaks	+LPS unique peaks
Number of peaks (% of total S5P RNAPII*)	2,490 (24.9%)	3,341 (36.2%)	328 (5.8%)	2,162 (49.4%)	3,013 (83.9%)
% of peaks located in promoter	14.7%	15.1%	19.8%	14.2%	14.6%

(* % of the total number of S5P RNAPII peaks described in table 1).

### Sp1 binding sites are increased upon LPS stimulation

Similar to S5P RNAPII data, ChIP-Seq analysis of Sp1 binding showed a large number of LPS-induced unique Sp1 peaks [Table 3]. Approximately half of LPS-unique Sp1 peaks localized to known promoters and regions with high CpG content. Combined analysis showed that of Sp1 peaks overlapping both S5P RNAPII and H3Ac, over 90%, are found in known promoters, regardless of CpG content [Table S5].

**Table 3:** Summary of Sp1 ChIP-seq identified peaks and their locations.

		SP1 ChIP-Seq Results			
	−LPS	+LPS	Common Peaks (+/−LPS)	−LPS Unique peaks	+LPS Unique peaks
Total number of Sp1 peaks	5,041 (202 bp)	8,254 (270 bp)	3,240 (n/a)	1,801 (137 bp)	5,090 (216 bp)
% of peaks located in promoter	74.1%	69.5%	91.8%	42.4%	55.4%
High CpG island content Peaks	3,655 (244 bp)	5,836 (316 bp)	2,955 (n/a)	700 (159 bp)	2,927 (269 bp)
% Located in promoter	93%	90.7%	93.8%	89.6%	87.2%
Low CpG island content Peaks	1,386 (145 bp)	2,418 (160 bp)	285 (n/a)	1,101 (124 bp)	2,163 (145 bp)
% Located in promoter	24.4%	18.4%	70.9%	12.4%	12.4%

Average peaks length for Sp1 showed in brackets for each group of peaks.(n/a), not applicable; the common peaks represent those that are present in both −LPS and +LPS, and the average length of these are shown in the −LPS and +LPS peaks columns.

### Integrated analysis of gene expression and ChIP-seq data in response to LPS stimulation reveals two distinct subgroups of inflammatory genes

The chromatin modification (H3Ac) and binding of S5P RNAPII and Sp1 were analysed in the subset of differentially regulated genes with a known role or function with respect to inflammation. We performed a gene-by-gene integrated analysis of the chromatin changes and genomic motifs present in the up- and down-regulated genes identified. Genes were grouped based on the presence or absence of significant H3Ac, S5P RNAPII and Sp1 ChIP-seq peaks [Table S6] and CpG islands content in proximal promoter region. *In silico* TATA box core promoter analysis of these genes was also included [Table S7]. We identified two groups of inflammatory genes regarding chromatin and promoter features, gene expression and gene class.

### Group 1 genes are up-regulated in response to LPS but typically lack H3 acetylation in their promoters, which are also low in CpG content

**Figure 4:**
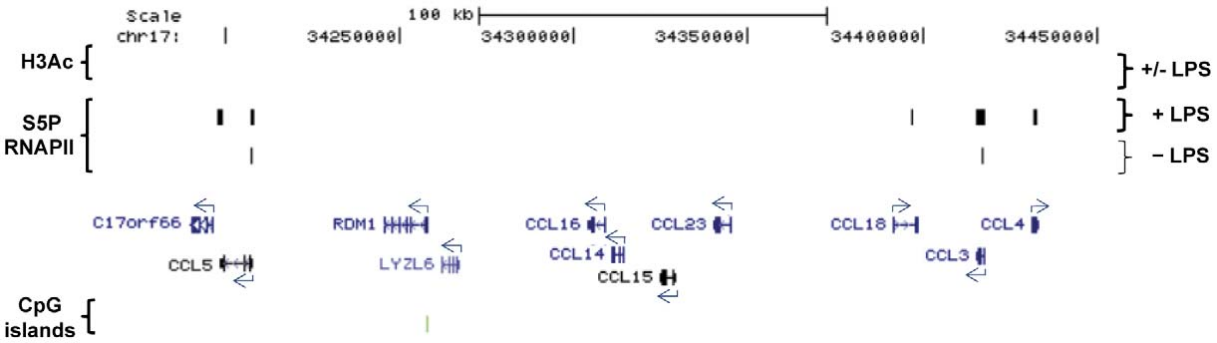
Overview of the human CCL cluster. The CCL chemokines cluster is an example of a genomic region lacking any significant H3Ac peaks. It is also a region with a low presence of CpG islands. The significant up-regulated genes of the cluster, *CCL4* and *CCL18*, contain S5P RNAPII binding. Genes identified by RefSeq using the UCSC browser are shown with blue arrows indicating TSSs locations and directions (GRCh37/hg19).

This group contained inflammatory mediators (mainly interleukins and the CCL chemokine subfamily) that were found to be up-regulated despite the fact that their promoters lack the typical H3Ac mark of actively regulated transcription, as well as having a low CpG content [Table 4]. However a few genes of this group had a binding signal for S5P RNAPII upon LPS stimulation [Figure 4]. The integrated analysis was extended in order to examine the regulatory pattern in other chemokine, interleukin and interferon family members (including their respective receptors) regardless of whether their expression was changed significantly by LPS treatment [Table S6]. An example of the group 1 is the CCL family cluster on chromosome 17 where H3Ac was absent for most of the genes in this cluster while S5P RNAPII promoter binding was found for a number of genes of the family cluster [Figure 4]. Genes included in this group also lacked active Sp1 binding [Table 4, Table S6 and data not shown]. Furthermore, genes in this group generally contained a classical TATA box in the annotated core promoter [Table 4 and Table S7]. Within the group 1 genes, we also found other inflammation-related genes sharing all of these features [Table 5 and Tables S6 and S7].

**Table 4:** Up-regulated Cytokine family members and their promoter composition.

CC Chemokine family										
					Annotated promoter composition					
Gene	Chr. Pos	TSS position	P-value	Fold change	Common H3Ac	Peak extension	S5P RNAPII	Sp1	CpG island	TATA box
*CCL1*	17q12	32,691,253	3.50E-02	3.10	NO	-	NO	NO	NO	NO
*CCL4*	17q12	34,431,220	5.20E-04	29.59	NO	-	+LPS	NO	NO	YES
*CCL18*	17q2	34,391,643	9.62E-05	2.76	NO	-	NO	NO	NO	YES
*CCL20*	2q36	228,678,558	4.50E-03	5.97	NO	-	+LPS	NO	NO	YES
*CCL22*	16q13	57,392,718	4.80E-02	2.18	NO	-	NO	NO	NO	NO

Interleukins and Interleukin receptors										
*IL1A*	2q14	113,543,971	1.20E-04	28.62	NO	-	NO	NO	NO	YES
*IL23A*	12q13	56,732,663	4.42E-06	17.87	NO	-	+LPS	NO	NO	YES
*IL27B*	19p13	4,229,540	5.36E-03	2.98	NO	-	+LPS	NO	NO	NO
*IL1R1*	2q12	102,770,401	4.58E-02	2.43	NO	-	NO	NO	NO	YES
		102,759,246			YES	→	−LPS	NO	YES	NO
		102,686,836			NO	-	NO	NO	NO	NO
*IL7R*	5p13	35,856,991	3.60E-04	5.13	NO	-	NO	NO	NO	YES
*IL18R1^#^*	2q12	^#^102,979,093	8.13E-05	5.62	NO	-	NO	NO	NO	YES
		102,927,962			NO	-	NO	NO	NO	YES

This is a summary of the results of gene expression and ChIP-seq data integration.

Table shows **gene** name and **chromosome position** (Chr.Pos); **TSS position**, annotated transcription start site (hg19); **P-value** (Student's t-test) and **fold change** due to LPS stimulation; SICER **H3Ac peaks** presence in annotated promoter (−2 Kb/+1 Kb of TSS in hg19); the effect of LPS, **Peak extension**; direction of chromatin expansion (→, upstream only; ←, downstream only; -, no expansion); S5P RNAP II peaks (MACS) in annotated promoters (−2 Kb/+1 Kb of TSS in hg19); Sp1 peaks (MACS) in annotated promoters (−2 Kb/+1 Kb of TSS in hg19); **CpG island** presence in annotated promoter (−2 Kb/+1 Kb of TSS); **TATA box** *in silico* finding (−150 bp/+50 bp of TSS). **#** in a 5 prime- upstream distance this gene has a positive signal for H3Ac and CpG islands.

**Table 5:** Inflammation related genes without histone acetylation in their annotated promoters.

					Annotated promoter composition					
Gene	Chr. Pos	TSS position	P-value	Fold change	CommonH3Ac	Peak extension	S5P RNAPII	Sp1 Peak	CpG Island	TATA Box
*ATP2B1*	12q21.33	90,020,392	2.82E-3	2.95	NO	-	NO	NO	NO	YES
		90,049,844			NO	-	NO	NO	NO	NO
*BCL2A1*	15q25.1	80,263,643	3.32E-04	2.76	NO	-	NO	NO	NO	YES^Δ^
*CD69*	12p13.31	9,804,764	8.74E-06	5.11	NO	-	NO	NO	NO	YES
*EREG*	4q13.3	75,230,860	4,68E-02	2.80	NO	-	NO	NO	NO	YES
*GPR84*	12q13.3	54,758,258	1.20E-02	2.01	NO	-	NO	NO	NO	NO
*KYNU*	2q22.2	143,635,195	6.80E-04	3.71	NO	-	NO	NO	NO	YES
*NAV3*	12q21.2	78,511,807	2.81E-02	2.19	NO	-	NO	NO	NO	YES
		78,430,643			NO	-	NO	NO	NO	NO
		78,225,069			NO	-	NO	NO	NO	NO
*NIACR1*	12q24.31	123,187,904	1.06E-02	3.84	NO	-	NO	NO	NO	NO
*NIACR2*	12q24.31	121,767,392	8.01E-04	8.78	NO	-	NO	NO	NO	NO
*OLR1*	12p13.2	10,320,198	4.75E-04	3.47	NO	-	NO	NO	NO	NO
		10,324,790			NO	-	NO	NO	NO	NO
*SERPINB2*	18q21.33	61,554,939	2.88E-02	3.05	NO	-	NO	NO	NO	YES
		61,564,325			NO	-	NO	NO	NO	YES
*SLAMF7*	1q23.3	160,709,077	2.91E-02	2.47	NO	-	NO	NO	NO	NO
*STAT4*	2q32.3	191,934,497	2.24E-02	2.51	NO	-	NO	NO	NO	NO
		192,015,925			NO	-	+LPS	NO	NO	NO
*TNFAIP6*	2q23.3	152,214,105	1.27E-04	19.21	NO	-	NO	NO	NO	NO
*UNQ9364*	6p23	14,124,115	1.54E-02	8.45	NO	-	NO	NO	NO	NO
U*NQ9368*	4q35.1	185,719,451	1.08E-02	7.57	NO	-	NO	NO	NO	NO

Listed are representative genes of the results from the integration of gene expression and ChIP-seq data.

Table shows **gene** name and **chromosome position** (Chr.Pos); **TSS position**, annotated transcription start site (hg19); **P-value** (Student's t- test) and **fold change** due to LPS stimulation; **H3Ac peaks** presence in annotated promoter (−2 Kb/+1 Kb of TSS, hg19); the effect of LPS, **Peak extension**; direction of chromatin expansion (→, upstream only; ←, downstream only; -, no expansion); S5P RNAPII peaks (MACS) in annotated promoters (−2 Kb/+1 Kb of TSS in hg19); Sp1 peaks (MACS) in annotated promoters (−2 Kb/+1 Kb of TSS in hg19); **CpG island** presence in annotated promoter (−2 Kb/+1 Kb of TSS); **TATA box** *in silico* finding (−150 bp/+50 bp of TSS) (Δ; TATA box *in silico* finding downstream of the TSS).

**Figure 5:**
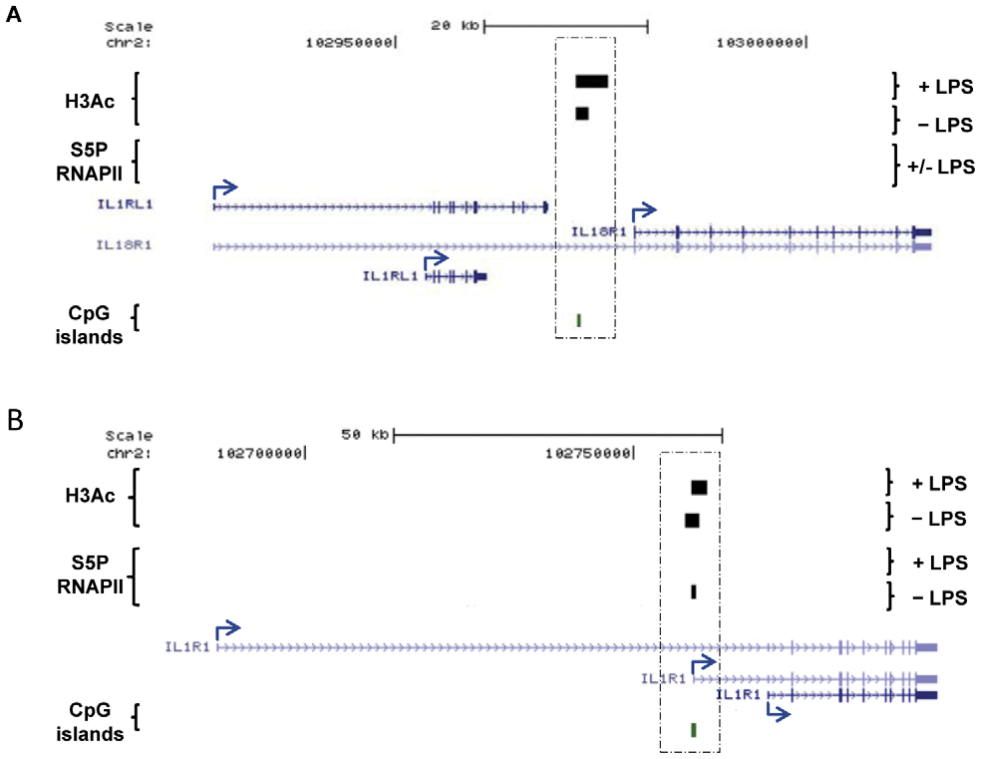
Group 1: Up-regulated cytokines. (A) *IL18R1* lacks a proximal promoter H3Ac peak but LPS opens it to within 3 kb of TSS. Alternative annotated promoters/TSSs of IL18R1 did not contain signals for H3Ac or RNAPII within a 2 kb distance upstream of the known TSS. However, an H3Ac peak is present approximately 5 kb upstream of the TSS (highlighted by the square) and in LPS-stimulated macrophages it is increased in size and comes to within 3 kb of the TSS. (B) The *IL1R1* intermediate isoform promoter harbours a common H3Ac peak and a unique S5P RNAPII peak in its promoter suggesting specific regulation of the gene at this annotated TSS in macrophages. Genes identified by RefSeq using the UCSC browser are shown with blue arrows indicating TSSs locations and directions.

There are some exceptions among the interleukin family. For instance *IL1B* has a number of features of group 1, i.e. presence of a TATA box and a lack of CpG content and S5P RNAPII binding at its annotated promoter [Table 6]. However, we have included this gene in the second group (described below) due to a common H3Ac peak present in its annotated promoter, which is expanded even further upstream in LPS-stimulated cells. Similarly, *IL18R1* conforms to the overall group 1 pattern, but a H3Ac peak could be found outside its proximal promoter (approximately 5 kb upstream) that overlaps a CpG island [Figure 5A]. In +LPS condition the H3Ac peak expanded downstream to just within 3 kb of the proximal promoter.

**Table 6:** Inflammation gene members with histone acetylation in their annotated promoters.

					Annotated promoter compositon					
Gene	Chr. Pos	TSS position	P-value	Fold change	Common H3Ac	Peak extension	P5S RNAPII	Sp1	CpG Island	TATA Box
*CD40*	2q13.12	44,746,906	2.15E-04	3.66	YES	↔	+LPS	+LPS	YES	NO
*CSF1*	1p13.3	110,453,233	4.59E-02	2.27	YES	→	−LPS	NO	YES	NO
*CXCL1*	4q21	74,735,109	5.30E-03	14.09	YES	←	+LPS	NO	YES	YES
*CXCL2*	4q21	74,965,998	1.94E-07	24.61	YES	←	+LPS	NO	YES	NO
*F3*	1p21.3	95,007,371	3.25E-03	2.12	YES	-	−LPS	+LPS	YES	NO
*ICAM1*	19p13.2	10,381,517	8.44E-04	4.97	YES	↔	+/−LPS	NO	YES	NO
*IL1B*	2q14	113,594,356	8.40E-03	2.12	YES	←	NO	NO	NO	YES
*IRAK2*	3p25.3	10,206,563	5.01E-04	5.17	YES	↔	+/−LPS	NO	YES	NO
*NFKB1*	4q24	103,422,486	2.00E-04	3.35	YES	-	+/−LPS	NO	YES	NO
		103,498,871			NO	-	NO	NO	NO	NO
*NFKB2*	10q24	104,155,500	2.00E-03	2.46	YES	↔	NO	+/−LPS	YES	NO
		104,154,229			YES	↔	NO	NO	YES	NO
*NFKBIA*	14q13	35,874,347	1.42E-04	6.44	YES	↔	+/−LPS	NO	YES	NO
*NFKBIZ*	3q12	101,546,857	2.85E-06	21.95	NO	-	NO	NO	NO	YES
		101,568,358			YES	↔	+/−LPS	+/−LPS	YES	NO
*PTGS2*	1q25	186,650,560	5.30E-04	26.32	YES	←	+/−LPS	NO	YES	YES
*PTX3*	3q25.32	157,154,580	1.08E-03	7.10	YES	→	+/−LPS	NO	YES	NO
*SOCS3*	17q25.3	76,356,158	5.83E-07	3.84	YES	↔	+/−LPS	+LPS	YES	NO
*TNF*	6p21.3	31,543,350	6.87E-06	47.23	YES	↔	−LPS	NO	NO	YES
*TNFAIP2*	14q32	103,592,664	4.46E-05	13.37	YES	↔	NO	NO	YES	NO
		103,599,087			YES	↔	NO	NO	NO	NO
*TNFAIP3*	6q23	138,188,581	1.09E-05	9.15	YES	→	+/−LPS	+/−LPS	YES	NO
*TNFAIP8*	5q23.1	118,604,418	1.23E-03	4.19	YES	←	+/−LPS	+/−LPS	YES	NO
		118,668,870			NO	-	NO	NO	NO	NO
		118,691,596			YES	→	+LPS	NO	YES	NO

Listed are representative genes of the results from the integration of gene expression and ChIP-seq data.

Table shows **gene name** and **chromosome position** (Chr.Pos); **TSS position**, annotated transcription start site (hg19); **P-value** (Student's t- test) and **fold change** due to LPS stimulation; **H3Ac peaks** presence in annotated promoter (−2 Kb/+1 Kb of TSS, hg 19); the effect of LPS, **Peak extension**; direction of chromatin expansion (→, upstream only; ←, downstream only; ↔ up- and downstream; -, no expansion); P5S RNAPII peaks (MACS) in annotated promoters (−2 Kb/+1 Kb of TSS in hg19); Sp1 peaks (MACS) in annotated promoters (−2 Kb/+1 Kb of TSS in hg19); **CpG island** presence in promoter (−2 Kb/+1 Kb of TSS); **TATA box** *in silico* finding (−150 bp/+50 bp of TSS).

Group 1 also includes *IL1R1*. This gene has three alternative TSSs/promoters. Expression microarray data did not allow us to determine which one of the TSSs/promoters was involved in the observed up-regulation of *IL1R1.* The upstream and downstream TSSs/promoters followed the pattern of group 1 while the third TSS/promoter located between the other two fulfilled part of the characteristics of group 2 (described below), having a H3Ac peak that was shifted downstream in response to LPS stimulation and this promoter bound S5P RNAPII in unstimulated cells [Table 4, Figure 5B] which suggest that this is promoter is actively regulated.

### Group 2 genes are up-regulated in response to LPS and contain H3 acetylation in their promoter regions, which are high in CpG content

**Figure 6:**
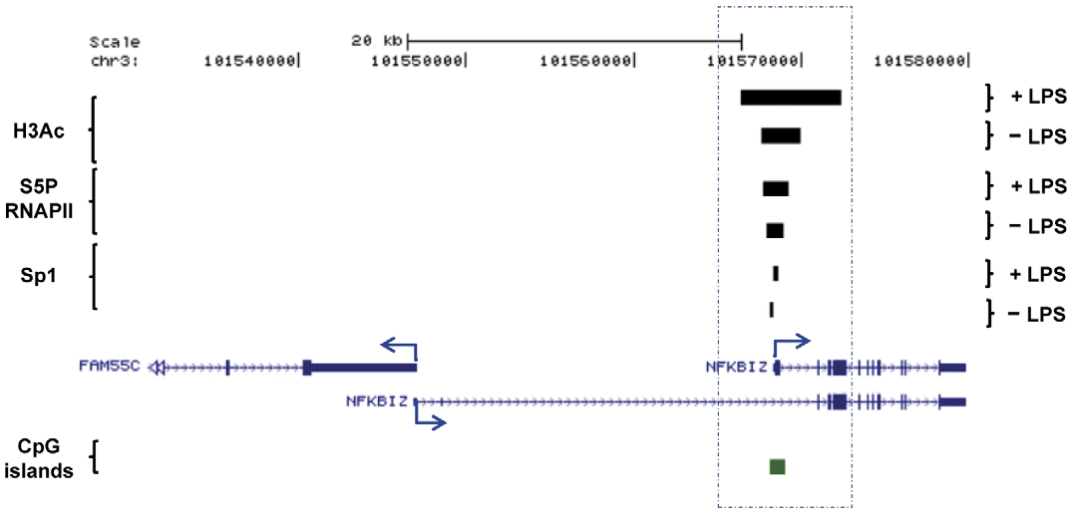
Group 2: Inflammatory up-regulated gene. *NFKBIZ* is shown as a representative cytokine gene with an alternative TSS. No H3Ac peaks or CpG islands were identified for the longer transcript. In the promoter area of the shorter transcript, an H3Ac peak is identified, which increases in size upon LPS stimulation. Genes identified by RefSeq using the UCSC browser are shown with blue arrows indicating TSSs locations and directions (GRCh37/hg19).

This group of inflammatory genes includes highly up-regulated genes such as *TNF*, and a number of genes encoding TNF-induced proteins, *NFkBIZ*, *CXCL1–2*, and *PTGS2* [Table 6 & Table S6]. A general feature of this group is the presence of common H3Ac peaks in the proximal promoters, which are generally expanded upon LPS stimulation (except for the promoters of the modestly up-regulated *CXCL3*, *PTGER2* and *NFKB1* genes where no expansion was observed). Other common features of this group are that the annotated gene promoters generally had S5P RNAPII (16 TSSs out of 25; Table 6) high CpG content (CpG islands) and Sp1 binding, the latter however to a lesser extent (7 out of 25 TSSs). Furthermore, they generally lacked a classical TATA box in their core promoter [Table 6 & Table S7]. As in group 1, there were some genes in this group with alternative TSSs/promoters that did not share all of the features of the group [Table 6 & Table S6]. This is exemplified by the *NFKBIZ*, which had two annotated TSSs/promoters where an H3Ac peak was found only within the promoter of the shortest transcript which also contained S5P RNAPII and Sp1 peaks in both conditions [Figure 6].

**Figure 7:**
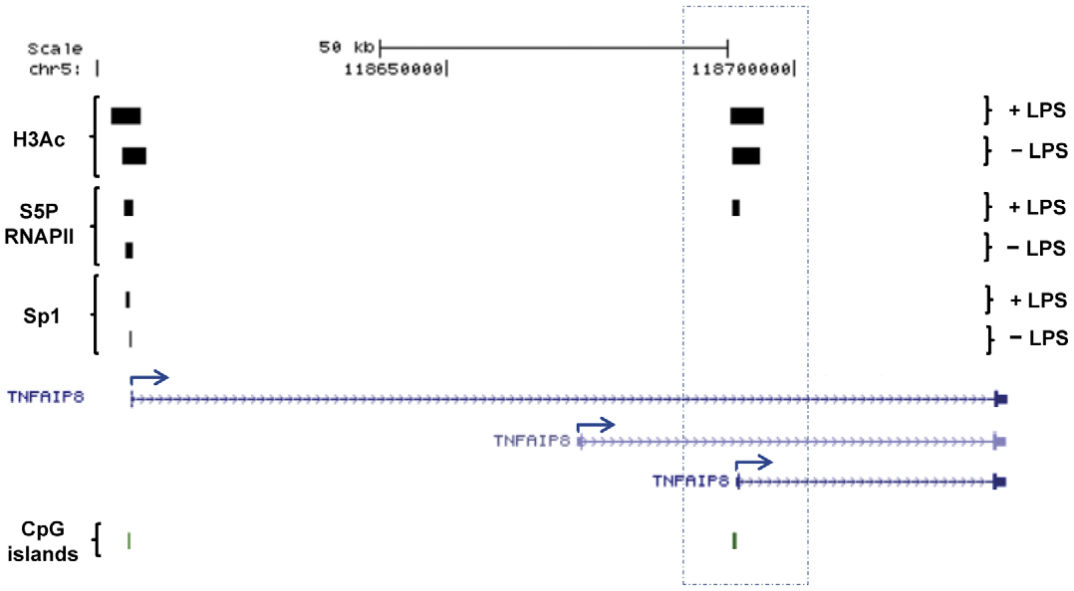
Group 2: Inflammatory up-regulated gene. *TNFAIP8 is* a representative cytokine gene where a new S5P RNAPII binding appears in one of its annotated promoter/TSS. This gene has three alternatives annotated promoters/TSS. The largest transcript has H3Ac peaks, S5P RNAPII and Sp1 binding nearby the TSS. The shortest transcript also harbours an H3Ac signal in its annotated TSS and an inducible S5P RNAPII signal upon LPS stimulation while the medium transcript does not fulfil the group 2 features. Genes identified by RefSeq using the UCSC browser are shown with blue arrows indicating TSSs locations and directions (GRCh37/hg19).

Similarly, *TNFIAP8* is annotated with three alternative promoters, of which; two exhibited the H3Ac and S5P RNAPII pattern of group 2, while one conformed to group 1 [Figure 7].

## Discussion

In this study, we used two genome-wide approaches, ChIP-seq and gene expression profiling, to examine in detail how an LPS-induced immune response affects chromatin remodelling and binding of Sp1 and the S5P RNA polymerase II initiation complex at the macrophage promoters. We investigated the relation of the early phase promoter remodelling/activation and gene expression after 2 hours of LPS stimulation. We found that a relatively low proportion of genes exhibit a detectable significant difference in transcription levels (>+/−2 fold change) after 2 hrs (181 up-regulated and 26 down-regulated). However, the S5P RNA PII data indicated that a major transcriptional reprogramming had occurred already at this early time point (4,371 unique S5P RNAPII peaks disappearing and 3,591 appearing following LPS stimulation). The S5P RNAPII mark represents a promoter-bound initiated RNAPII complex preceding elongation [11], [23]. Thus, S5P RNAPII marked promoters appearing after LPS stimulation could reflect genes poised for transcription, albeit with not yet detectable changes in gene expression levels. Unique S5P RNAPII peaks that disappear upon stimulation could be interpreted as either genes with inactivated transcription or genes where the RNAPII is in elongation phase. The *TNF* gene would be one example of the latter, where an S5P RNAPII peak disappeared upon LPS-stimulation, coinciding with a significant increase in transcription (47-fold up-regulation).

The H3K9Ac is one of the most widely studied active histone modifications, and is known to be associated with transcription initiation of genes by opening up chromatin, thereby allowing the transcription machinery to assemble at the core promoter. It is also a marker of transcription initiation sites of actively initiated and transcribed genes [21], [22], [24]. When specifically focusing on this marker in inflammatory mediators and response genes with altered expression levels after 2 hrs LPS treatment, several interesting observations were made. Given the effect that LPS has on the macrophage transcriptome [2], we expected either an appearance or disappearance of H3Ac peaks upon LPS-stimulation. Surprisingly, few of the H3Ac peaks identified were unique to either +/−LPS-stimulated cells, while the majority of the identified peaks overlapped in genomic location. This indicates that these gene promoters are transcriptionally enabled already prior to stimulation, and the widening/expansion observed following stimulation among these peaks would indicate a further unpacking of chromatin.There was no consistent pattern among the differentially expressed genes regarding in which direction the expansion occurred, i.e. down- or upstream [Table 6], and in some genes the expression was in both directions. Many of the up-regulated genes had S5P RNAPII present in their annotated promoter region already in the unstimulated condition [Table S6], suggesting that the transcription machinery has been assembled and is poised for a rapid transcriptional up-regulation following 2 hrs of LPS stimulation [Table S6].

The expansion of H3Ac peaks was a general feature of our ChIP-seq dataset; although the majority of gene promoters that had an expansion of the H3Ac peak in response to LPS stimulation (10,029 peaks out of 11,834 expanded) did not have a significant change in expression levels of the corresponding genes. This may reflect the early state of the inflammatory activation we have studied, and it may be that transcription initiation has occurred but not yet elongation. Recruitment of transcription factors, such as the nuclear factor NF-(kappa)B (NF-κB) and other inducible factors, is required to enhance the frequency of transcription initiation, elongation and pre-mRNA processing [23], [25]. Thus, the expansion of the H3Ac peaks that we see may also reflect the assembly of the transcription complex in these genes. The mammalian NF-κB family consists of RelA (p65), RelB, c-Rel, p50, and p52 [26], and it was shown by Saccani et al that p65 recruitment and histone acetylation are important for early primary response gene expression in murine macrophages [27]. In a study looking at the binding of NF-κB in human monocytic cells before and after 1 hour of LPS stimulation by Schreiber et al, they found that in unstimulated cells, p50 and p52 were already bound to a large number of target gene promoters in the presence of RNA polymerase II [28]. After LPS-stimulation, additional NF-κB subunits bound to these and other genes, and these were the most likely to show an increase in gene expression. If some NF-κB subunits are already in place with RNA polymerase II, this may be a reason why we see so many H3Ac marks over the whole macrophage genome before LPS treatment. Widening of the open chromatin region would allow the other subunits, such as p65 and c-Rel to bind, and this could result in a quick initiation of gene transcription when needed.

Genes that had significant changes in expression levels (>2 fold change) following LPS-stimulation in our study fell into two groups regarding the features of their proximal promoters. In the first group of genes, there was a total lack of the H3Ac mark in their annotated promoter regions, which were also low in CpG content but had TATA boxes. Also, they tended to lack Sp1 binding under both conditions. This pattern was seen in genes from the following families - chemokines such as the CCL subfamily members and interleukins. Despite this there was still a significant up-regulation in gene expression levels, e.g. nearly a 30-fold up-regulation of *IL1A* and *CCL4* [Table 4]. Individual regulation of these cytokine genes could be due to the presence of TATA-boxes, which together with the low content/absence of CpG islands in their promoter regions are features of genes with narrow single peak TSSs. These are associated with tightly regulated non-house keeping genes that can be specifically induced in response to external stimuli [29], [30]. The second group of genes were characterized by H3 acetylation, high CpG content and a lack of TATA boxes in their annotated promoter regions. This group included a number of highly up-regulated proinflammatory genes such as *TNF* and *PTGS2.* This group conforms to the pattern previously described by Roh et al [31]. The transcription factor Sp1 has been found to recruit TATA-binding protein to promoters lacking TATA boxes [30], [32], and consistent with this, a large proportion of the genes in this group was found to have Sp1 peaks in their promoters [Table 6 & Table S6]. These are features that have been reported for broad promoters, where the position of individual TSSs are less precise than in TATA-box containing promoters [23], [30].

In a previous study of undifferentiated and phorbol 12-myristate 13-acetate (PMA) differentiated THP-1 cells (no LPS stimulation), Krantz et al. used ChIP-chip combined with deep sequencing of 5′-ends of transcripts (DeepCAGE) to analyze H3K9Ac patterns in a subset of 4,481 promoters filtered from a total of 14,607 active promoters [33]. They identified three groups of genes based on the location of the H3K9Ac peaks, which were located upstream, downstream and centered around the TSS, of which the latter two groups were enriched in broad promoters with high CpG content, which would fall into the group 2 that we described above. They found that these tended to regulate genes with a higher gene expression than peak promoters [33].

Our studies were also performed in transformed THP-1 macrophages, except we stimulated them with LPS for 2 hours. The genes of group 1 that lack H3Ac marks may be specific for macrophages under influence of inflammatory stimuli, but as the study by Kratz et al excluded 70% of the DeepCAGE-identified promoters in their analysis, it is not possible to draw any conclusions from the comparison in this respect [33]. It must be remembered that histone modifications are very dynamic and change rapidly; we are examining them very early during the inflammatory response, using one specific mark. It could be that alternative chromatin modifications are at work in the promoter regions that render them accessible to the transcription initiation machinery for the group of genes without H3Ac marks but with TATA boxes.

On a genome-wide scale, i.e. not restricting the analysis to genes with measurable differential expression, we found that a large number of the unique S5P RNAPII binding sites do not overlap known annotated promoter regions. This was especially evident for the LPS-induced unique S5P RNAPII sites where about 70% followed this pattern. The fact that they are outside previously unannotated promoters could indicate a cell-type restricted pattern where in macrophage cells; LPS treatment results in recruitment of novel promoters. Kratz et al found that of the 14,607 promoters identified in THP-1 cells from DeepCAGE data, 16% were unannotated [33]. Using cap-analysis of gene expression (CAGE), Carninci et al. previously demonstrated the existence of macrophage-restricted alternative promoter usage upon inflammatory stimulation [30]. Our data indicate that there is a significant number of additional, previously not annotated alternative promoters involved in the early response to inflammation in macrophage cells.

Using large datasets of chromatin histone modification and RNAPII binding, initiation and elongation from different cell types, including the K562 monocytic cell line, Chen et al. showed that joint enrichment of the H3K9Ac, H3K27Ac, H3K4me2 andH3K4me3 were necessary for RNAPII enrichment at the promoter, and that the acetylation marks of H3K9 and H3K27 were most predictive of active promoter usage [34]. We here analysed the H3K9Ac mark. Wang et al previously showed that H3K4 methylation is needed for H3K9 acetylation, with both marks being necessary for the recruitment and initiation of RNAPII at a promoter (but not sufficient for elongation). Thus, our H3Ac data should be sufficient to indicate promoter regions that could bind the S5P RNAPII initiation complex. However, we found that the majority (83.9%) of the LPS-induced unique S5P RNAPII sites did not co-localise with H3Ac-modified regions. This indicates that alternative mechanisms for RNAPII recruitment and transcription initiation exist in LPS-stimulated macrophage cells.

In summary, our data reported here demonstrates the role that histone modifications, specifically H3Ac, have in the macrophage LPS-mediated early immune response. However, important groups of immune response mediators (such as chemokines and interleukins) showed an independent transcriptional regulation mechanism, where H3Ac plays a minor role. Furthermore, our results point to the existence of a significant number of previously unannotated alternative promoters in LPS-stimulated macrophages. We show that many changes are early and correlate with changes in the number of genes being actively transcribed, and provide novel insights into the epigenetic mechanisms that control transcription and gene expression in response to LPS.

## Supporting Information

Text S1 **Supplementary** **Materials and Methods** **.**
(DOCX)

Figure S1 **Differentiation of THP-1 monocytes to macrophages.** Panel A) Light microscopy photographs of cultured THP-1 monocytes and macrophages. Upper panel A.1 show the normal monocytic cell phenotycpic (round cells grown in suspension). After 24 h of culturing in conditioned media the THP-1 monocytes changed to be adherent flattened macrophage-like cells (A.2). Magnification: 20×. Panel B) Surface *CD11b* expression by FACS. Expression of the surface marker CD11b was analyzed in THP-1 monocytes and differentiated macrophages by immunofluorescence by FACS. Conditioned media differentiated macrophages (MΦ-CM) presented a shift in the mean of fluorescence intensity (MΦ-CM = 9.0 MFI) compared to THP-1 monocytic cells (6.7 MFI) after 24 hours. A similar shift in fluorescence intensity was seen when phorbol 12-myristate 13-acetate (PMA, 50 ng/ml) was used for 24 hours (MΦ-PMA = 11.2 MFI).
(TIF)

Figure S2 **LPS increases the area of already present H3Ac peaks.** The expansion of significant H3Ac peaks is illustrated in the graphic for the *RLF* gene. H3Ac peaks identified by SICER are presented in two formats below in the graphic: Upper panel **a**: +LPS condition representation of the tag density, where in first instance a sharp H3Ac peak could be noted adjacent to the gene transcription start site (TSS). Lower but still significant tag coverage peaks (FDR <10E-3), comprise the widening/expansion of the H3Ac peak along the gene promoter upon stimuli (* indicates the significant acetylated peaks after LPS) Upper panel **b**: +LPS the black strait-bar shows the DNA coverage (from the start to the end point) of the significant peaks identified by SICER. Bottom panel **a**: In the −LPS condition the presence of an H3Ac peak is confined to a location nearby the TSS of the *RLF* gene. Black arrows indicate two chromatin regions without significant acetylation (gap size >1.2 kb each), maximum acetylation gap allowed in SICER was (800 bp). Bottom panel **b**: −LPS the black strait-bar shows the DNA coverage (from the start to the end point) of the significant peaks identified by SICER. *RLF* gene annotated by RefSeq is shown using the UCSC browser, the blue arrow indicating TSS locations and directions (GRCh37/hg19). The presence/absence of CpG Islands is also shown.
(TIF)

Table S1 **List of all genes that were significantly up- or down-regulated by a fold change of ≥+/−2.** A) Listing of all increased genes with a fold change due to the LPS treatment ≥2. Table shows gene symbol (RefSeq hg19), P-value (student's t-test); fold change due to LPS stimulation and gene name (RefSeq hg19). B) Listing of all decreased genes with a fold change due to the LPS treatment ≥−2. Table shows gene symbol (RefSeq hg19), P-value (student's t-test); fold change due to LPS stimulation and gene name (RefSeq hg19).
(DOCX)

Table S2 **Gene Ontology and Disease categories for significantly up- and down-regulated genes (by a fold change of ≥+/−2). Upper and bottom tables** show the Gene Ontology (GO) category description and the disease association categories (MeSH) for the up and down-regulated genes, respectively. Count in category represent the number up- or down-regulated genes the fell into the annotation categories with a statistical significance, P-value (Fisher's Exact Test). (GENOMATIX software v.2 (http://www.genomatix.de).
(DOCX)

Table S3 **Replicated H3Ac peaks in biological replicates.**
(DOCX)

Table S4 ***In silico*** **transcription binding search in unique S5P RNAPII peaks.** Upper table summarize the *in silico* TF search for unique S5P RNAPII peaks (either −LPS or LPS+) that OVERLAP with H3Ac peaks. Bottom table shows the results of the *in silico* TF search for unique S5P RNAPII peaks (either −LPS or LPS+) NOT overlapping H3Ac peaks. Z-score represents the distance from the population mean in units of the population standard deviation. (Genomatix (Matrix Library Version 8.4).
(DOCX)

Table S5 **Summary of the combined analysis of H3Ac, S5P RNAPII and Sp1 ChIP-seq peaks and their locations.**
(DOCX)

Table S6 **Summary of the integration analysis of ChIP-seq data and expression profile in macrophages after LPS.** Tables show gene name and chromosome position (Chr.Pos); P-value (Student's t-test); fold change due to LPS stimulation; [P-value and fold change: – no available expression data] TSS position, annotated transcription start site (hg19); H3AC common peak: presence (YES) or absence (NO) of a significant H3Ac peaks in promoter (−2 Kb/+1 Kb of TSS) for both +/−LPS conditions.; the effect of LPS, Peak extension; direction of chromatin expansion (→, upstream only; ←, downstream only; -, no expansion); S5P RNAPII & Sp1 peaks: presence of a significant peak in unstimulated condition (−LPS), LPS treated cells (+LPS); common in both conditions (+/−LPS) or absence in either condition (NO) in promoters (−2 Kb/+1 Kb of TSS); CpG island presence or absence in promoter (−2 Kb/+1 Kb of TSS); TATA box *in silico* finding (−150 bp/+50 bp of TSS). RED BOLD: 105 genes included in MeSH inflammatory category. Tables are divided into gene families and classified groups.
(DOC)

Table S7 **MatchInspector** ***in silico*** **TATA Box finding.** Tables show the results of *in silico* TATA box finding in the subset of up- and down-regulated genes upon LPS stimulation in a distance of −150 bp/+50 bp of RefSeq gene annotated TSS. Gene symbol, Position of the TATA box from 5′UTR in base pairs (bp), gene strand direction (+, forward; −, reverse), promoter sequence (Lowercase DNA sequence letters: promoter area (Upstream 5′UTR), Uppercase DNA sequence: 5′UTR; and Underlined and in bold: TATA box sequence), TATA box category (VTATA.01: gTATAAAa, VTATA.02: ctATAAAA), and matrix similarity (cut off ≥0.9). (MatchInspector, Genomatix).
(DOCX)
