# Correction: Combined Chromatin and Expression Analysis Reveals Specific Regulatory Mechanisms within Cytokine Genes in the Macrophage Early Immune Response

**DOI:** 10.1371/annotation/689ae222-9eb1-4f80-8ebe-6b80e8ad45ce

**Published:** 2012-04-13

**Authors:** Maria Jesus Iglesias, Sarah-Jayne Reilly, Olof Emanuelsson, Bengt Sennblad, Mohammad Pirmoradian Najafabadi, Lasse Folkersen, Anders Mälarstig, Jens Lagergren, Per Eriksson, Anders Hamsten, Jacob Odeberg

There was an error in the citation. The correct citation is: Iglesias MJ, Reilly S-J, Emanuelsson O, Sennblad B, Pirmoradian Najafabadi M, et al. (2012) Combined Chromatin and Expression Analysis Reveals Specific Regulatory Mechanisms within Cytokine Genes in the Macrophage Early Immune Response. PLoS ONE 7(2): e32306. doi:10.1371/journal.pone.0032306 

