# Supplementary material for: Combined Chromatin and Expression Analysis Reveals Specific Regulatory Mechanisms within Cytokine Genes in the Macrophage Early Immune Response
Source: PLoS One. 2012 Feb 27;7(2):e32306. doi: 10.1371/journal.pone.0032306 (PMC3288078; doi:10.1371/journal.pone.0032306)
Supplement: Text S1 — Supplementary Materials and Methods . (DOCX) [file pone.0032306.s001.docx]

**Iglesias et al. Supplementary Data**

**Text S1. Supplementary Materials and Methods**

**FACS analysis**

Differentiation of THP-1 cells from monocytes to macrophages was confirmed by analyzing *CD11b* expression using FACS [1] THP-1 monocytes and differentiated THP-1 macrophages were harvested and washed in phosphate buffer saline (PBS). Cells were stained using Pacific Blue™ anti-human *CD11b* antibody human antibody (clone ICRFF4) (Biolegend, San Diego, CA, USA) in a PBS/1%BSA (Bovine Serum Albumin) solution for 15 minutes on ice. Excess antibody was removed by washing twice in PBS. Cells were then fixed with 2% paraformaldehyde solution and examined using a BD FACSCalibur™ flow cytometer (BD Biosciences, San Jose, CA, USA). Results were analyzed using Summit® V3.3 software (DakoCytomation Denmark A/S, Glostrup, Denmark).

**mRNA extraction, cDNA preparation and qPCR**

To confirm an inflammatory response cDNA was synthesized using the SuperScript^TM^ II reverse transcriptase enzyme following the manufacturer’s instructions (Invitrogen, Carlsbad, CA, USA). qPCR was performed using Taqman® gene expression assays for *TNF* (Hs_00174128_m1) and the house keeping gene *RPLPO* (Hs_99999902_m1) in an ABIprism 7000 system (Applied Biosystems, Foster City, CA, USA). qPCR was also used to confirm differentiation of THP-1 monocytes to macrophages (described above), using gene expression assays for *CD11b* (Hs_00167304_m1) and *RPLPO*. Relative fold changes in gene expression between experimental conditions were calculated using the 2^-ΔΔct^ method [2]

**Microarray gene expression analysis**

Quality controls comprised Agilent Bioanalyzer examination and visual inspection of scans. CEL files were RMA normalized and log2 transformed using the apt-probe-set-summarize algorithm as implemented in Affymetrix Power Tools 1.12.0. Meta probe sets were selected from the Affymetrix defined *core* set of well-established genes. Genes with low expression values in both experimental conditions (log2exp<5.5) were considered as absent and were discarded from the analysis. This arbitrary expression threshold was chosen based on similar experiments in which it excluded most Y-chromosome gene expression levels in female samples. Expressed genes were analyzed for differential expression in pairs of treatment conditions. The expression array data is available from gene expression omnibus under accession number GSE32141.

**ChIP verification**

The ChIPs were validated using Power SYBR® Green PCR master mix in an ABIprism 7000 system (Applied Biosystems, Foster City, CA). All reactions were performed in triplicate in a final volume of 25μl. Relative enrichment between experimental conditions was assayed using the 2^-ΔΔct^ method [2] and was also scored visually by comparing the PCR amplification from ChIPed IgG DNA and input DNAs. H3Ac and S5P RNAPII ChIP enrichment was addressed using primers for transcriptionally active euchromatin (GPH10001C(+)01A; human *GAPDH*), inactive euchromatin (GPH00002C(+), human *MYOD1*) and heterochromatin (GPH00003C(+); human *SAT2*) (SABiosciences, Frederick, MD, USA) were used. The human dihydrofolate reductase (DHFR) promoter primers and human interleukin 6 (*IL6*) promoter primers were used as positive and negative control, respectively, for Sp1 ChIP enrichment. *DHFR* primers sequence (Sp1 ChIP KIT; Upstate, Charlottesville, VA, USA) Forward: TCGCCTGCACAAATAGGGAC and Reverse: AGAACGCGCGGTCAAGTTT. *IL6* primers sequence (Thermo Fisher Scientific, Wilmington, Delaware USA): Forward=TCGTGCATGACTTCAGCTT; Reverse= ACGTCCTTTAGCATGGCAAG.

**Peak calling**

Mapping quality checks: Sequence reads were 35 bases or longer but truncated to 35 bases to ensure base quality over the entire read. Reads were aligned to the human reference genome (GRCh37/hg19) with Burrows-Wheeler Alignment tool (BWA) [3] and only uniquely mapped reads with a maximum of 2 mismatched bases were considered for further analysis. The sequence tag density generated from the input library (total DNA) was used as background.

SICER analysis for H3Ac [4]: Enriched islands (regions) were identified using the input sample as control library. The SICER window size was set to 200 bp (base pairs), fragment size to 300 bp, and gap size adjusted to be 800 bp. This generated a set of candidate islands with individual p-values calculated using control library as background. Minimum number of tags in a qualify window was two and the number of redundant copies of identical tags in a library was set to 1 tag. Candidate islands were further filtered on an FDR threshold of 1E-3, resulting in a set of significant islands (peaks).

MACS analysis for S5P RNAP II and Sp1: Sequencing tags library from two independent biological experimental either S5P RNAP II or Sp1 were concatenated and merged as suggested in Zhang et al; 2008 [5]. Resulting datasets were used to define enriched regions along the macrophage genomes using MACS, a ChIP-Seq peak-finding algorithm [5]. We ran the analysis using the input DNA sample as control library and we set a p-value threshold of 10E-5 to define significant enriched regions. A tag size of 35 bp was used and the other algorithm parameters were left to default.

References:

1. Rios F, Jancar S, Melo I, Ketelhuth D, Gidlund M (2008) Role of PPAR-gamma in the Modulation of CD36 and FcgammaRII induced by LDL with Low and high degrees of oxidation during the differentiation of the monocytic THP-1 cell line. Cell Physiol Biochem 22: 549-556
2. Livak KJ and Schmittgen TD (2001) Analysis of relative gene expression data using real-time quantitative PCR and the 2(-Delta Delta C(T)) Method) Methods 25: 402-408
3. Li H and Durbinv R (2009) Fast and accurate short read alignment with Burrows-Wheeler transform. Bioinformatics 25: 1754-60
4. Zang C, Schones DE, Zeng C, Cui K, Zhao K, et al. (2009) A clustering approach for identification of enriched domains from histone modification ChIP-Seq data. Bioinformatics 25: 1952-1958.
5. Zhang Y, Liu T, Meyer CA, Eeckhoute J, Johnson DS, et al. (2008) Model-based Analysis of ChIP-Seq (MACS). Genome Biology,. 9: p. R137.
