# Supplementary figures and images for: Combined Chromatin and Expression Analysis Reveals Specific Regulatory Mechanisms within Cytokine Genes in the Macrophage Early Immune Response
Source: PLoS One. 2012 Feb 27;7(2):e32306. doi: 10.1371/journal.pone.0032306 (PMC3288078; doi:10.1371/journal.pone.0032306)

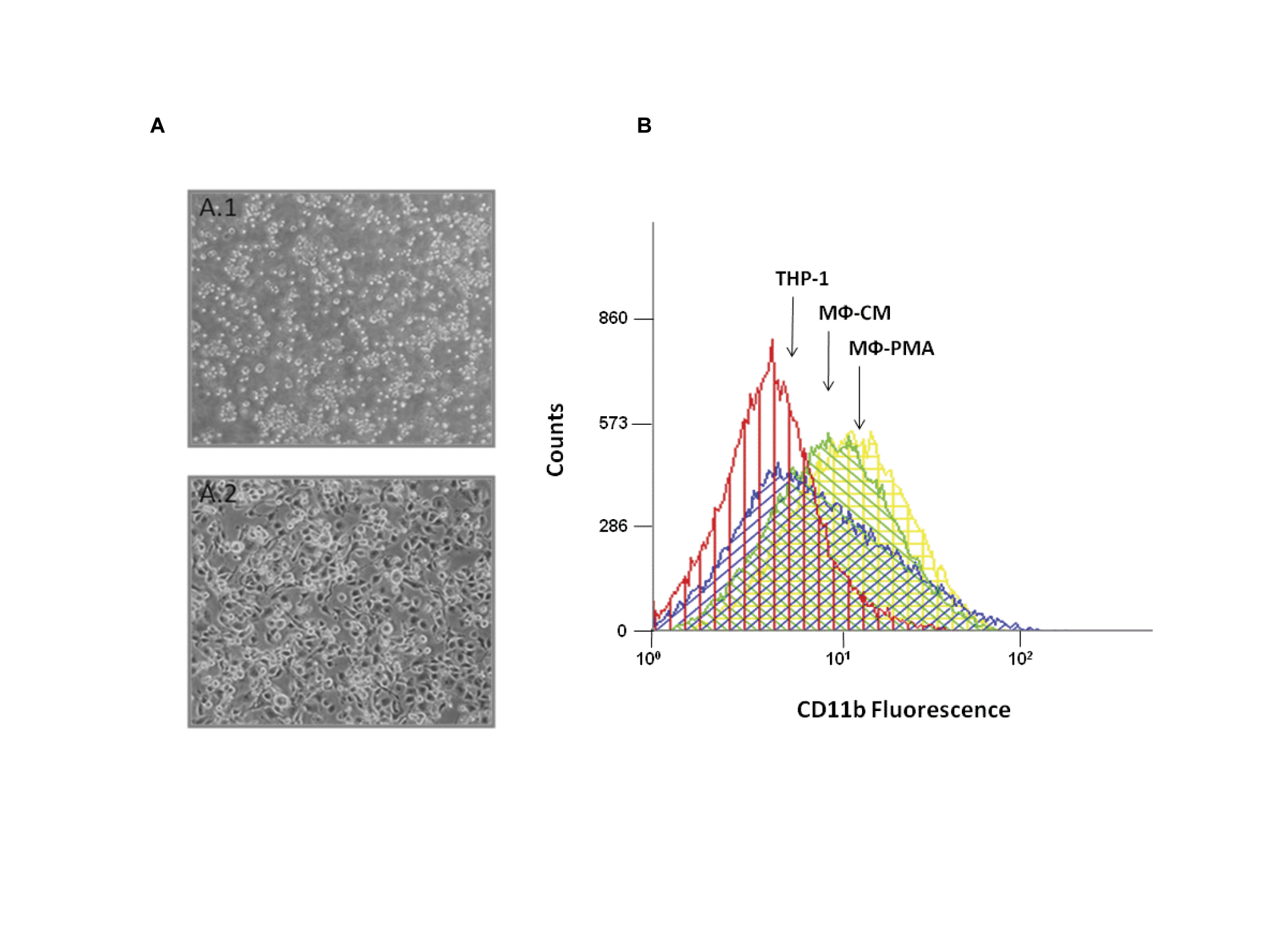

Supplement: Figure S1 — Differentiation of THP-1 monocytes to macrophages. Panel A) Light microscopy photographs of cultured THP-1 monocytes and macrophages. Upper panel A.1 show the normal monocytic cell phenotycpic (round cells grown in suspension). After 24 h of culturing in conditioned media the THP-1 monocytes changed to be adherent flattened macrophage-like cells (A.2). Magnification: 20×. Panel B) Surface CD11b expression by FACS. Expression of the surface marker CD11b was analyzed in THP-1 monocytes and differentiated macrophages by immunofluorescence by FACS. Conditioned media differentiated macrophages (MΦ-CM) presented a shift in the mean of fluorescence intensity (MΦ-CM = 9.0 MFI) compared to THP-1 monocytic cells (6.7 MFI) after 24 hours. A similar shift in fluorescence intensity was seen when phorbol 12-myristate 13-acetate (PMA, 50 ng/ml) was used for 24 hours (MΦ-PMA = 11.2 MFI). (TIF) [file pone.0032306.s002.tif]

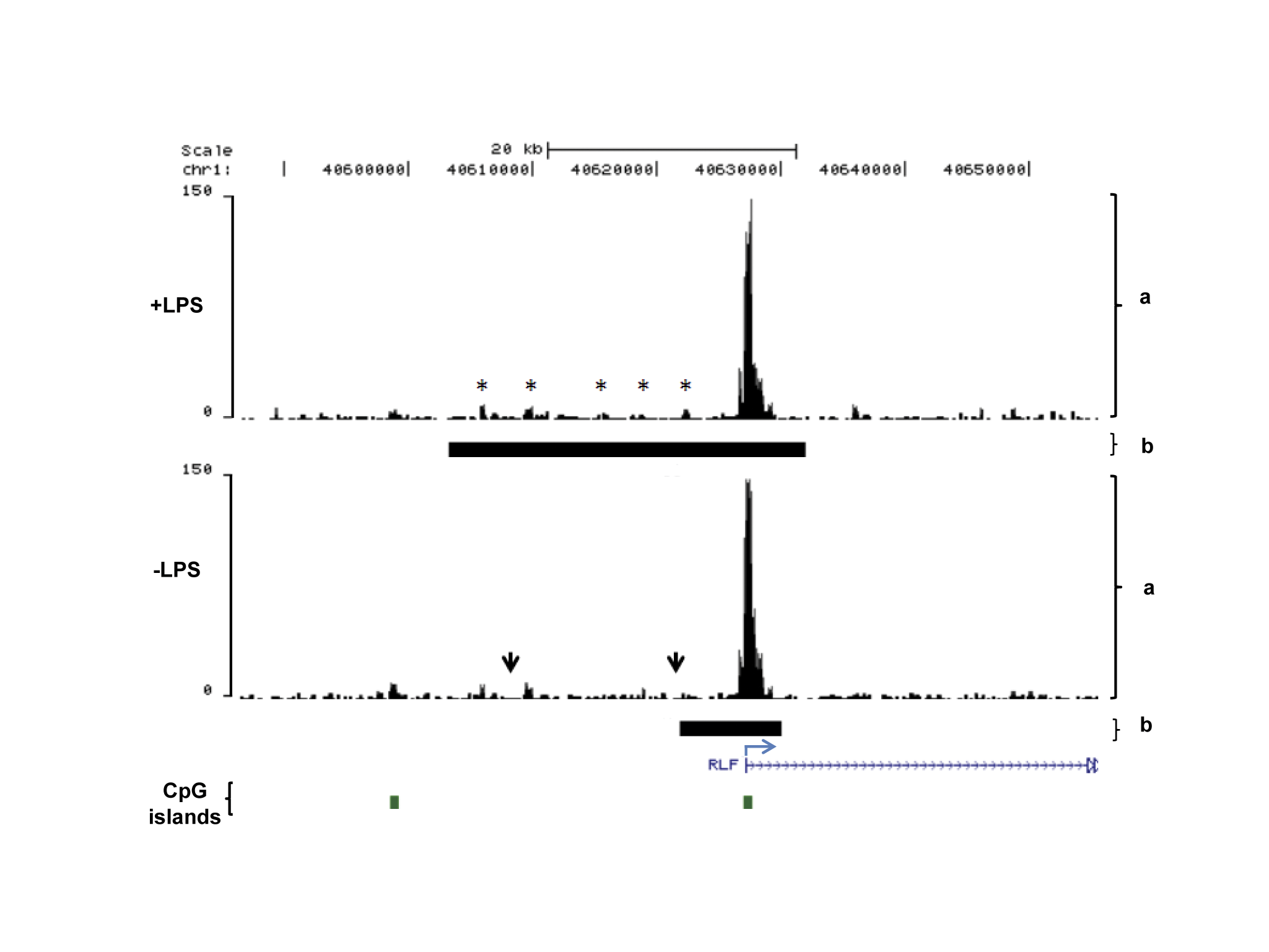

Supplement: Figure S2 — LPS increases the area of already present H3Ac peaks. The expansion of significant H3Ac peaks is illustrated in the graphic for the RLF gene. H3Ac peaks identified by SICER are presented in two formats below in the graphic: Upper panel a: +LPS condition representation of the tag density, where in first instance a sharp H3Ac peak could be noted adjacent to the gene transcription start site (TSS). Lower but still significant tag coverage peaks (FDR <10E-3), comprise the widening/expansion of the H3Ac peak along the gene promoter upon stimuli (* indicates the significant acetylated peaks after LPS) Upper panel b: +LPS the black strait-bar shows the DNA coverage (from the start to the end point) of the significant peaks identified by SICER. Bottom panel a: In the −LPS condition the presence of an H3Ac peak is confined to a location nearby the TSS of the RLF gene. Black arrows indicate two chromatin regions without significant acetylation (gap size >1.2 kb each), maximum acetylation gap allowed in SICER was (800 bp). Bottom panel b: −LPS the black strait-bar shows the DNA coverage (from the start to the end point) of the significant peaks identified by SICER. RLF gene annotated by RefSeq is shown using the UCSC browser, the blue arrow indicating TSS locations and directions (GRCh37/hg19). The presence/absence of CpG Islands is also shown. (TIF) [file pone.0032306.s003.tif]
