# Supplementary material for: Combined Chromatin and Expression Analysis Reveals Specific Regulatory Mechanisms within Cytokine Genes in the Macrophage Early Immune Response
Source: PLoS One. 2012 Feb 27;7(2):e32306. doi: 10.1371/journal.pone.0032306 (PMC3288078; doi:10.1371/journal.pone.0032306)
Supplement: Table S1 — List of all genes that were significantly up- or down-regulated by a fold change of ≥+/−2. A) Listing of all increased genes with a fold change due to the LPS treatment ≥2. Table shows gene symbol (RefSeq hg19), P-value (student's t-test); fold change due to LPS stimulation and gene name (RefSeq hg19). B) Listing of all decreased genes with a fold change due to the LPS treatment ≥−2. Table shows gene symbol (RefSeq hg19), P-value (student's t-test); fold change due to LPS stimulation and gene name (RefSeq hg19). (DOCX) [file pone.0032306.s004.docx]

**Table S1. List of all genes that were significantly up- or down-regulated by a fold change of ≥+/-2.**

1. **Up-regulated genes**

| **Gene symbol** | **P-value** | **Fold Change** | **Gene name** | | | | | | | |
| --- | --- | --- | --- | --- | --- | --- | --- | --- | --- | --- |
| *TNF* | 6.87E-06 | 47.23 | Tumor necrosis factor (TNF superfamily, member 2) | | | | | | | |
| *CCL4* | 5.27E-04 | 29.59 | Chemokine (C-C motif) ligand 4 | | | | | | | |
| *IL1A* | 1.20E-04 | 28.62 | Interleukin 1, alpha | | | | | | | |
| *PTGS2* | 5.30E-04 | 26.32 | Prostaglandin-endoperoxide synthase 2 (prostaglandin G/H synthase and cyclooxygenase) | | | | | | | |
| *CXCL2* | 1.94E-07 | 24.61 | Chemokine (C-X-C motif) ligand 2 | | | | | | | |
| *NFKBIZ* | 2.85E-06 | 21.95 | Nuclear factor of kappa light polypeptide gene enhancer in B-cells inhibitor, zeta | | | | | | | |
| *TNFAIP6* | 1.28E-04 | 19.21 | Tumor necrosis factor, alpha-induced protein 6 | | | | | | | |
| *IL23A* | 4.42E-06 | 17.87 | Interleukin 23, alpha subunit p19 | | | | | | | |
| *TRAF1* | 1.12E-04 | 15.49 | TNF receptor-associated factor 1 | | | | | | | |
| *CXCL1* | 5.30E-03 | 14.09 | Chemokine (C-X-C motif) ligand 1 (melanoma growth stimulating activity, alpha) | | | | | | | |
| *TNFAIP2* | 4.46E-05 | 13.37 | Tumor necrosis factor, alpha-induced protein 2 | | | | | | | |
| *BIRC3* | 1.27E-03 | 13.10 | Baculoviral IAP repeat-containing 3 | | | | | | | |
| *LAMP3* | 3.87E-04 | 11.53 | Lysosomal-associated membrane protein 3 | | | | | | | |
| *LIF* | 7.14E-04 | 10.86 | Leukemia inhibitory factor (cholinergic differentiation factor) | | | | | | | |
| *CTGF* | 2.93E-04 | 9.40 | Connective tissue growth factor | | | | | | | |
| *TNFAIP3* | 1.09E-05 | 9.15 | Tumor necrosis factor, alpha-induced protein 3 | | | | | | | |
| *NIACR2* | 8.01E-04 | 8.78 | Niacin receptor 2 | | | | | | | |
| *UNQ9364* | 1.54E-02 | 8.45 | Homo sapiens clone DNA175919 FLFF9364 (UNQ9364) mRNA, complete cds. | | | | | | | |
| *UNQ9368* | 1.08E-02 | 7.57 | RTFV9368 | | | | | | | |
| *GRAMD3* | 2.25E-06 | 7.36 | GRAM domain containing 3 | | | | | | | |
| *PTX3* | 1.08E-03 | 7.10 | Pentraxin-related gene, rapidly induced by IL-1 beta | | | | | | | |
| *RNF144B* | 1.08E-03 | 6.82 | Ring finger protein 144B | | | | | | | |
| *NFKBIA* | 1.42E-04 | 6.44 | Nuclear factor of kappa light polypeptide gene enhancer in B-cells inhibitor, alpha | | | | | | | |
| **Gene symbol** | **P-value** | **Fold Change** | **Gene name** | | | | | | | |
| *SOD2* | 4.42E-04 | 6.40 | Superoxide dismutase 2, mitochondrial | | | | | | | |
| *CCL20* | 4.51E-03 | 5.97 | Chemokine (C-C motif) ligand 20 | | | | | | | |
| *ZC3H12A* | 7.91E-05 | 5.80 | Zinc finger CCCH-type containing 12A | | | | | | | |
| *DUSP1* | 3.82E-03 | 5.78 | Dual specificity phosphatase 1 | | | | | | | |
| *IL18R1* | 8.13E-05 | 5.62 | Interleukin 18 receptor 1 | | | | | | | |
| *EHD1* | 5.26E-05 | 5.53 | EH-domain containing 1 | | | | | | | |
| *CD274* | 1.89E-02 | 5.48 | CD274 molecule | | | | | | | |
| *SERPINE2* | 3.41E-03 | 5.46 | Serpin peptidase inhibitor, clade E (nexin, plasminogen activator inhibitor type 1), member 2 | | | | | | | |
| *IER3* | 1.40E-03 | 5.46 | Immediate early response 3 | | | | | | | |
| *MOP-1* | 2.18E-02 | 5.44 | MOP-1 | | | | | | | |
| *IRAK2* | 5.01E-04 | 5.17 | Interleukin-1 receptor-associated kinase 2 | | | | | | | |
| *REL* | 5.26E-04 | 5.17 | v-rel reticuloendotheliosis viral oncogene homolog (avian) | | | | | | | |
| *IL7R* | 3.66E-04 | 5.13 | Interleukin 7 receptor | | | | | | | |
| *CD69* | 8.74E-06 | 5.11 | CD69 molecule | | | | | | | |
| *RNF19B* | 1.17E-03 | 5.11 | Ring finger protein 19B | | | | | | | |
| *CXCL3* | 1.80E-03 | 5.10 | Chemokine (C-X-C motif) ligand 3 | | | | | | | |
| *ICAM1* | 8.44E-04 | 4.97 | Intercellular adhesion molecule 1 | | | | | | | |
| *GBP2* | 2.84E-04 | 4.91 | Guanylate binding protein 2, interferon-inducible | | | | | | | |
| *ADORA2A* | 1.07E-04 | 4.90 | Adenosine A2a receptor | | | | | | | |
| *C6orf145* | 6.36E-04 | 4.84 | Chromosome 6 open reading frame 145 | | | | | | | |
| *PLAU* | 4.57E-05 | 4.73 | Plasminogen activator, urokinase | | | | | | | |
| *GPR132* | 6.10E-06 | 4.54 | G protein-coupled receptor 132 | | | | | | | |
| *KCNA3* | 4.36E-06 | 4.44 | Potassium voltage-gated channel, shaker-related subfamily, member 3 | | | | | | | |
| *CD83* | 1.85E-03 | 4.43 | CD83 molecule | | | | | | | |
| *MCOLN2* | 9.65E-05 | 4.37 | Mucolipin 2 | | | | | | | |
| *GEM* | 1.67E-02 | 4.24 | GTP binding protein overexpressed in skeletal muscle | | | | | | | |
| *ZBTB10* | 1.19E-06 | 4.20 | Zinc finger and BTB domain containing 10 | | | | | | | |
| *TNFAIP8* | 1.23E-03 | 4.19 | Tumor necrosis factor, alpha-induced protein 8 | | | | | | | |
| **Gene symbol** | **P-value** | **Fold Change** | **Gene name** | | | | | | | |
| *STX11* | 4.43E-03 | 3.93 | Syntaxin 11 | | | | | | | |
| *HIVEP2* | 2.38E-03 | 3.86 | Human immunodeficiency virus type I enhancer binding protein 2 | | | | | | | |
| *NIACR1* | 1.06E-02 | 3.84 | Niacin receptor 1 |  |  |  |  |  | | |
| *SOCS3* | 5.83E-07 | 3.84 | Suppressor of cytokine signaling 3 | | |  |  |  | | |
| *DLL4* | 2.02E-02 | 3.83 | Delta-like 4 (Drosophila) | |  |  |  |  | | |
| *RIPK2* | 8.72E-04 | 3.79 | Receptor-interacting serine-threonine kinase 2 | | | |  |  | | |
| *KYNU* | 6.80E-04 | 3.71 | Kynureninase (L-kynurenine hydrolase) | | |  |  |  | | |
| *PDE4B* | 1.11E-04 | 3.69 | Phosphodiesterase 4B, cAMP-specific (phosphodiesterase E4 dunce homolog, Drosophila) | | | | | | | |
| *TNIP1* | 6.56E-04 | 3.69 | TNFAIP3 interacting protein 1 | |  |  |  |  |  |  |
| *PMAIP1* | 8.83E-03 | 3.68 | Phorbol-12-myristate-13-acetate-induced protein 1 | | | | |  |  |  |
| *CD40* | 2.15E-04 | 3.66 | CD40 molecule, TNF receptor superfamily member 5 | | | | |  |  |  |
| *NR4A3* | 7.33E-03 | 3.63 | Nuclear receptor subfamily 4, group A, member 3 | | | |  |  |  |  |
| *RASL11A* | 1.99E-03 | 3.53 | RAS-like, family 11, member A | | |  |  |  |  |  |
| *CFLAR* | 8.01E-04 | 3.49 | CASP8 and FADD-like apoptosis regulator | | | |  |  |  |  |
| *OLR1* | 4.75E-04 | 3.47 | Oxidized low density lipoprotein (lectin-like) receptor 1 | | | | |  |  |  |
| *NLRP3* | 6.87E-04 | 3.46 | NLR family, pyrin domain containing 3 | | |  |  |  |  |  |
| *TIFA* | 4.07E-03 | 3.42 | TRAF-interacting protein with forkhead-associated domain | | | | |  |  |  |
| *STAT5A* | 1.17E-03 | 3.41 | Signal transducer and activator of transcription 5A | | | |  |  |  |  |
| *IL8* | 1.82E-02 | 3.36 | Interleukin 8 |  |  |  |  |  |  |  |
| *NFKB1* | 2.07E-04 | 3.35 | Nuclear factor of kappa light polypeptide gene enhancer in B-cells 1 | | | | | |  |  |
| *E2F7* | 8.28E-04 | 3.32 | E2F transcription factor 7 | |  |  |  |  |  |  |
| *PNRC1* | 1.28E-04 | 3.29 | Proline-rich nuclear receptor coactivator 1 | | | |  |  |  |  |
| *GCH1* | 6.21E-04 | 3.28 | GTP cyclohydrolase 1 | |  |  |  |  |  |  |
| *PELI1* | 1.20E-02 | 3.28 | Pellino homolog 1 (Drosophila) | | |  |  |  |  |  |
| *CCRN4L* | 2.04E-04 | 3.24 | CCR4 carbon catabolite repression 4-like (S. cerevisiae) | | | | |  |  |  |
| *CD44* | 1.89E-03 | 3.18 | CD44 molecule (Indian blood group) | | |  |  |  |  |  |
| *SDC4* | 5.63E-04 | 3.14 | Syndecan 4 |  |  |  |  |  |  |  |
| *PPP1R15A* | 4.11E-03 | 3.13 | Protein phosphatase 1, regulatory (inhibitor) subunit 15A | | | | |  |  |  |
| **Gene symbol** | **P-value** | **Fold Change** | **Gene name** | | | | |  |  |  |
| *DDIT4* | 3.91E-03 | 3.10 | DNA-damage-inducible transcript 4 | | |  |  |  |  |  |
| *CCL1* | 3.53E-02 | 3.10 | Chemokine (C-C motif) ligand 1 | | |  |  |  |  |  |
| *MAP3K8* | 2.37E-04 | 3.08 | Mitogen-activated protein kinase kinase kinase 8 | | | |  |  |  |  |
| *MARCKS* | 5.79E-04 | 3.05 | Myristoylated alanine-rich protein kinase C substrate | | | | |  |  |  |
| *SERPINB2* | 2.88E-02 | 3.05 | Serpin peptidase inhibitor, clade B (ovalbumin), member 2 | | | | |  |  |  |
| *FOSL2* | 4.01E-04 | 3.05 | FOS-like antigen 2 |  |  |  |  |  |  |  |
| *PSTPIP2* | 4.15E-03 | 3.03 | Proline-serine-threonine phosphatase interacting protein 2 | | | | |  |  |  |
| *WTAP* | 5.66E-05 | 3.02 | Wilms tumor 1 associated protein | | |  |  |  |  |  |
| *ICAM4* | 2.75E-03 | 3.02 | Intercellular adhesion molecule 4 (Landsteiner-Wiener blood group) | | | | | |  |  |
| *ITGB8* | 5.83E-03 | 3.01 | Integrin, beta 8 |  |  |  |  |  |  |  |
| *PLEKHF2* | 4.11E-03 | 2.99 | Pleckstrin homology domain containing, family F (with FYVE domain) member 2 | | | | | | |  |
| *BTG2* | 1.13E-02 | 2.99 | BTG family, member 2 | |  |  |  |  |  |  |
| *EBI3* | 5.37E-03 | 2.98 | Epstein-Barr virus induced 3 | |  |  |  |  |  |  |
| *MST4* | 8.19E-05 | 2.97 | Serine/threonine protein kinase MST4 | | |  |  |  |  |  |
| *ATP2B1* | 2.83E-03 | 2.95 | ATPase, Ca++ transporting, plasma membrane 1 | | | |  |  |  |  |
| *PVRL3* | 2.35E-02 | 2.87 | Poliovirus receptor-related 3 | |  |  |  |  |  |  |
| *DENND5A* | 1.69E-02 | 2.87 | DENN/MADD domain containing 5A | | |  |  |  |  |  |
| *ABTB2* | 1.39E-02 | 2.86 | Ankyrin repeat and BTB (POZ) domain containing 2 | | | |  |  |  |  |
| *PLK3* | 4.48E-03 | 2.85 | Polo-like kinase 3 (Drosophila) | | |  |  |  |  |  |
| *RASGEF1B* | 3.36E-02 | 2.82 | RasGEF domain family, member 1B | | |  |  |  |  |  |
| *EREG* | 4.68E-02 | 2.80 | Epiregulin |  |  |  |  |  |  |  |
| *GIMAP5* | 3.44E-04 | 2.77 | GTPase, IMAP family member 5 | | |  |  |  |  |  |
| *BCL2A1* | 3.32E-04 | 2.76 | BCL2-related protein A1 | |  |  |  |  |  |  |
| *PTGER4* | 2.82E-03 | 2.76 | Prostaglandin E receptor 4 (subtype EP4) | | |  |  |  |  |  |
| *CCL18* | 9.62E-05 | 2.76 | Chemokine (C-C motif) ligand 18 (pulmonary and activation-regulated) | | | | | |  |  |
| *TNIP2* | 2.08E-03 | 2.73 | TNFAIP3 interacting protein 2 | |  |  |  |  |  |  |
| *KCNN2* | 5.15E-04 | 2.72 | Potassium intermediate/small conductance calcium-activated channel, subfamily N, member 2 | | | | | | | |
| *TXNDC11* | 5.43E-04 | 2.71 | Thioredoxin domain containing 11 | | |  |  |  |  |  |

| **Gene symbol** | **P-value** | **Fold Change** | **Gene name** | | | |  | |  | |  | |  | |  | |  |
| --- | --- | --- | --- | --- | --- | --- | --- | --- | --- | --- | --- | --- | --- | --- | --- | --- | --- |
| *JDP2* | 5.22E-04 | 2.69 | Jun dimerization protein 2 | | | |  | |  | |  | |  | |  | |  |
| *MSC* | 1.01E-02 | 2.67 | Musculin (activated B-cell factor-1) | | | | | |  | |  | |  | |  | |  |
| *DUSP2* | 4.86E-03 | 2.66 | Dual specificity phosphatase 2 | | | |  | |  | |  | |  | |  | |  |
| *PTGER2* | 2.45E-02 | 2.63 | Prostaglandin E receptor 2 (subtype EP2), 53kDa | | | | | | | |  | |  | |  | |  |
| *SERPINB9* | 1.94E-02 | 2.63 | Serpin peptidase inhibitor, clade B (ovalbumin), member 9 | | | | | | | | | |  | |  | |  |
| *TP53INP1* | 1.67E-03 | 2.62 | Tumor protein p53 inducible nuclear protein 1 | | | | | | | |  | |  | |  | |  |
| *TWIST1* | 1.79E-04 | 2.62 | Twist homolog 1 (Drosophila) | | | |  | |  | |  | |  | |  | |  |
| *C13orf29* | 3.91E-02 | 2.60 | Chromosome 13 open reading frame 29 | | | | | |  | |  | |  | |  | |  |
| *OGFRL1* | 2.12E-04 | 2.59 | Opioid growth factor receptor-like 1 | | | | | |  | |  | |  | |  | |  |
| *TBC1D10A* | 4.57E-03 | 2.59 | TBC1 domain family, member 10A | | | | | |  | |  | |  | |  | |  |
| *ACSL1* | 1.01E-04 | 2.57 | Acyl-CoA synthetase long-chain family member 1 | | | | | | | |  | |  | |  | |  |
| *MFSD2A* | 2.83E-04 | 2.57 | Major facilitator superfamily domain containing 2 | | | | | | | |  | |  | |  | |  |
| *CLDN1* | 3.72E-02 | 2.57 | Claudin 1 |  |  | |  | |  | |  | |  | |  | |  |
| *ARL5B* | 1.07E-02 | 2.56 | ADP-ribosylation factor-like 5B | | | | | |  | |  | |  | |  | |  |
| *ETV3* | 7.21E-03 | 2.55 | Ets variant 3 | |  | |  | |  | |  | |  | |  | |  |
| *PLAGL2* | 7.56E-05 | 2.54 | Pleiomorphic adenoma gene-like 2 | | | | | |  | |  | |  | |  | |  |
| *TRIP10* | 1.22E-02 | 2.53 | Thyroid hormone receptor interactor 10 | | | | | |  | |  | |  | |  | |  |
| *STAT4* | 2.24E-02 | 2.51 | Signal transducer and activator of transcription 4 | | | | | | | |  | |  | |  | |  |
| *ETS2* | 9.60E-04 | 2.49 | v-ets erythroblastosis virus E26 oncogene homolog 2 (avian) | | | | | | | | | |  | |  | |  |
| *SLAMF7* | 2.92E-02 | 2.47 | SLAM family member 7 | | | |  | |  | |  | |  | |  | |  |
| *NFKB2* | 2.01E-03 | 2.46 | Nuclear factor of kappa light polypeptide gene enhancer in B-cells 2 (p49/p100) | | | | | | | | | | | | | |  |
| *NFE2L2* | 1.35E-04 | 2.46 | Nuclear factor (erythroid-derived 2)-like 2 | | | | | | | |  | |  | |  | |  |
| *DRAM1* | 1.87E-02 | 2.46 | Damage-regulated autophagy modulator 1 | | | | | |  | |  | |  | |  | |  |
| *GADD45A* | 4.83E-03 | 2.45 | Growth arrest and DNA-damage-inducible, alpha | | | | | | | | |  | |  | |  |  |
| *IL1R1* | 4.59E-02 | 2.43 | Interleukin 1 receptor, type I | | | | |  | |  | |  | |  | |  |  |
| *RAP2C* | 2.13E-05 | 2.42 | RAP2C, member of RAS oncogene family | | | | | | |  | |  | |  | |  |  |
| *FAM49A* | 2.86E-03 | 2.42 | Family with sequence similarity 49, member A | | | | | | | | |  | |  | |  |  |
| *RASSF5* | 1.32E-03 | 2.40 | Ras association (RalGDS/AF-6) domain family member 5 | | | | | | | | | | |  | |  |  |
| **Gene symbol** | **P-value** | **Fold Change** | **Gene name** | | | | |  | |  | |  | |  | |  |  |
| *GFPT2* | 2.19E-02 | 2.40 | Glutamine-fructose-6-phosphate transaminase 2 | | | | |  | |  | |  | |  | |  |  |
| *RPL35* | 3.49E-02 | 2.40 | Ribosomal protein L35 | | | | |  | |  | |  | |  | |  |  |
| *B4GALT5* | 2.32E-03 | 2.39 | UDP-Gal:betaGlcNAc beta 1,4- galactosyltransferase, polypeptide 5 | | | | | | | | | | | | |  |  |
| *NINJ1* | 1.18E-04 | 2.34 | Ninjurin 1 | | |  | |  | |  | |  | |  | |  |  |
| *N4BP2L1* | 3.77E-03 | 2.34 | NEDD4 binding protein 2-like 1 | | | | | | |  | |  | |  | |  |  |
| *PIM2* | 1.21E-02 | 2.33 | Pim-2 oncogene | | |  | |  | |  | |  | |  | |  |  |
| *SAT1* | 3.28E-03 | 2.33 | Spermidine/spermine N1-acetyltransferase 1 | | | | | | | | |  | |  | |  |  |
| *NAMPT* | 6.80E-03 | 2.31 | Nicotinamide phosphoribosyltransferase | | | | | | | | |  | |  | |  |  |
| *KLF6* | 2.94E-02 | 2.29 | Kruppel-like factor 6 | | | | |  | |  | |  | |  | |  |  |
| *XBP1* | 7.84E-05 | 2.28 | X-box binding protein 1 | | | | |  | |  | |  | |  | |  |  |
| *CSF1* | 4.59E-02 | 2.27 | Colony stimulating factor 1 (macrophage) | | | | | | | | |  | |  | |  |  |
| *DUSP16* | 5.27E-05 | 2.27 | Dual specificity phosphatase 16 | | | | | | |  | |  | |  | |  |  |
| *LCP2* | 1.57E-02 | 2.24 | Lymphocyte cytosolic protein 2 (SH2 domain containing leukocyte protein of 76kDa) | | | | | | | | | | | | | | |
| *USP53* | 7.82E-04 | 2.23 | Ubiquitin specific peptidase 53 | | | | | | |  | |  | |  | |  |  |
| *HIVEP1* | 3.54E-03 | 2.21 | Human immunodeficiency virus type I enhancer binding protein 1 | | | | | | | | | | | | |  |  |
| *KLF7* | 1.27E-04 | 2.21 | Kruppel-like factor 7 (ubiquitous) | | | | | | |  | |  | |  | |  |  |
| *TP53BP2* | 1.34E-04 | 2.20 | Tumor protein p53 binding protein, 2 | | | | | | |  | |  | |  | |  |  |
| *SLC39A14* | 5.56E-03 | 2.19 | Solute carrier family 39 (zinc transporter), member 14 | | | | | | | | | | |  | |  |  |
| *NAV3* | 2.82E-02 | 2.19 | Neuron navigator 3 | | |  | |  | |  | |  | |  | |  |  |
| *TNFRSF9* | 1.76E-02 | 2.18 | Tumor necrosis factor receptor superfamily, member 9 | | | | | | | | | | |  | |  |  |
| *CCL22* | 4.81E-02 | 2.18 | Chemokine (C-C motif) ligand 22 | | | | | | |  | |  | |  | |  |  |
| *CRIM1* | 1.07E-02 | 2.17 | Cysteine rich transmembrane BMP regulator 1 (chordin-like) | | | | | | | | | | |  | |  |  |
| *PIM1* | 1.29E-02 | 2.16 | Pim-1 oncogene | | | | | | | | | | |  | |  |  |
| *LONRF1* | 5.48E-03 | 2.16 | LON peptidase N-terminal domain and ring finger 1 | | | | | | | | | | |  | |  |  |
| *JUNB* | 1.27E-03 | 2.16 | Jun B proto-oncogene | | | | | | | | | | |  | |  |  |
| *ATP1B1* | 1.41E-03 | 2.13 | ATPase, Na+/K+ transporting, beta 1 polypeptide | | | | | | | | | | | | | | |
| *F3* | 3.25E-03 | 2.12 | Coagulation factor III (thromboplastin, tissue factor) | | | | | | | | | | | | | | |
| *IL1B* | 8.48E-03 | 2.12 | Interleukin 1, beta | | | | | | | | | | | | | | |
| *GBP3* | 2.90E-02 | 2.10 | Guanylate binding protein 3 | | | | | | | | | | | | | | |
| **Gene symbol** | **P-value** | **Fold Change** | **Gene name** | | | | |  | |  | |  | |  | |  |  |
| *CBR3* | 3.19E-03 | 2.09 | Carbonyl reductase 3 | | | | | | | | | | | | | | |
| *WT1* | 3.36E-02 | 2.09 | Wilms tumor upstream neighbor 1 | | | | | | | | | | | | | | |
| *B3GNT5* | 9.94E-03 | 2.08 | UDP-GlcNAc:betaGal beta-1,3-N-acetylglucosaminyltransferase 5 | | | | | | | | | | | | | | |
| *TNFSF9* | 1.39E-02 | 2.07 | Tumor necrosis factor (ligand) superfamily, member 9 | | | | | | | | | | | | | | |
| *USP12* | 2.41E-03 | 2.07 | Ubiquitin specific peptidase 12 | | | | | | | | | | | | | | |
| *KANK1* | 2.94E-03 | 2.05 | KN motif and ankyrin repeat domains 1 | | | | | | | | | | | | | | |
| *CNKSR3* | 1.32E-02 | 2.05 | CNKSR family member 3 | | | | | | | | | | | | | | |
| *SLC7A2* | 8.03E-03 | 2.04 | Solute carrier family 7 (cationic amino acid transporter, y+ system), member 2 | | | | | | | | | | | | | | |
| *SLC43A2* | 1.16E-03 | 2.04 | Solute carrier family 43, member 2 | | | | | | | | | | | | | | |
| *PDSS1* | 4.26E-03 | 2.04 | Prenyl (decaprenyl) diphosphate synthase, subunit 1 | | | | | | | | | | | | | | |
| *PLEK* | 9.81E-04 | 2.02 | Pleckstrin | | | | | | | | | | | | | | |
| *GPR84* | 1.20E-02 | 2.01 | G protein-coupled receptor 84 | | | | | | | | | | | | | | |
| *RHOF* | 5.85E-03 | 2.00 | Ras homolog gene family, member F (in filopodia) | | | | | | | | | | | | | | |
| *ST20* | 2.36E-03 | 2.00 | Suppressor of tumorigenicity 20 | | | | | | | | | | | | | | |
| *PERP* | 2.55E-03 | 2.00 | PERP, TP53 apoptosis effector | | | | | | | | | | | | | | |
| *PDP1* | 1.69E-02 | 2.00 | Protein phosphatase 2C, Mg-dependent, catalytic subunit | | | | | | | | | | | | | | |
| *ARRDC3* | 7.43E-03 | 2.00 | Arrestin domain containing 3 | | | | | | | | | | | | | | |

1. **Down-regulated genes**

| **Gene symbol** | **P-value** | **Fold Change** | **Gene name** | |  |  |  |  |  |  |
| --- | --- | --- | --- | --- | --- | --- | --- | --- | --- | --- |
| *HHEX* | 1.39E-03 | -3.25 | Hematopoietically expressed homeobox | |  |  |  |  |  |  |
| *P2RY5* | 1.15E-04 | -2.98 | Purinergic receptor P2Y, G-protein coupled, 5 | | |  |  |  |  |  |
| *ARHGEF3* | 1.68E-03 | -2.79 | Rho guanine nucleotide exchange factor (GEF) 3 | | |  |  |  |  |  |
| *SPRY2* | 4.11E-02 | -2.77 | Sprouty homolog 2 (Drosophila) | |  |  |  |  |  |  |
| *ATG16L1* | 3.91E-03 | -2.74 | ATG16 autophagy related 16-like 1 (S. cerevisiae) | | |  |  |  |  |  |
| *C16orf54* | 2.55E-03 | -2.45 | Chromosome 16 open reading frame 54 | |  |  |  |  |  |  |
| *GPR65* | 1.92E-02 | -2.43 | G protein-coupled receptor 65 | |  |  |  |  |  |  |
| *USP2* | 4.39E-03 | -2.29 | Ubiquitin specific peptidase 2 |  |  |  |  |  |  |  |
| *ARHGAP12* | 3.26E-02 | -2.27 | Rho GTPase activating protein 12 | |  |  |  |  |  |  |
| *IMP3* | 5.27E-04 | -2.19 | IMP3, U3 small nucleolar ribonucleoprotein, homolog (yeast) | | | |  |  |  |  |
| *PDP2* | 3.98E-03 | -2.19 | Pyruvate dehydrogenase phosphatase isoenzyme 2 | | | |  |  |  |  |
| *CCR1* | 9.77E-03 | -2.14 | Chemokine (C-C motif) receptor 1 | |  |  |  |  |  |  |
| *LRRC33* | 5.39E-04 | -2.10 | Leucine rich repeat containing 33 | |  |  |  |  |  |  |
| *FRAT1* | 2.20E-03 | -2.05 | Frequently rearranged in advanced T-cell lymphomas | | | |  |  |  |  |
| *ZNF557* | 1.12E-02 | -2.04 | Zinc finger protein 557 |  |  |  |  |  |  |  |
| *ZNF828* | 1.39E-04 | -2.02 | Zinc finger protein 828 |  |  |  |  |  |  |  |
| *CEBPA* | 1.10E-02 | -2.02 | CCAAT/enhancer binding protein (C/EBP), alpha | | |  |  |  |  |  |
| *HERPUD1* | 1.15E-04 | -2.01 | Homocysteine-inducible, endoplasmic reticulum stress-inducible, ubiquitin-like domain member 1 | | | | | | | |
| *TMEM188* | 4.95E-04 | -2.01 | Transmembrane protein 188 |  |  |  |  |  |  |  |
| *ARL4C* | 4.82E-02 | -2.01 | ADP-ribosylation factor-like 4C | |  |  |  |  |  |  |
| *FAM78A* | 1.85E-03 | -2.01 | Family with sequence similarity 78, member A | | |  |  |  |  |  |
| *ZNF627* | 1.39E-03 | -2.00 | Zinc finger protein 627 | | |  |  |  |  |  |
| *PDIK1L* | 5.06E-03 | -2.00 | PDLIM1 interacting kinase 1 like | | |  |  |  |  |  |
| *SLC30A1* | *1.76E-02* | -2.00 | Solute carrier family 30 (zinc transporter), member 1 | | |  |  |  |  |  |
| *RNF168* | 3.97E-03 | -2.00 | Ring finger protein 168 | | |  |  |  |  |  |
| *TIGD2* | 8.03E-03 | -2.00 | Tigger transposable element derived 2 | | |  |  |  |  |  |
