# Supplementary material for: Combined Chromatin and Expression Analysis Reveals Specific Regulatory Mechanisms within Cytokine Genes in the Macrophage Early Immune Response
Source: PLoS One. 2012 Feb 27;7(2):e32306. doi: 10.1371/journal.pone.0032306 (PMC3288078; doi:10.1371/journal.pone.0032306)
Supplement: Table S2 — Gene Ontology and Disease categories for significantly up- and down-regulated genes (by a fold change of ≥+/−2). Upper and bottom tables show the Gene Ontology (GO) category description and the disease association categories (MeSH) for the up and down-regulated genes, respectively. Count in category represent the number up- or down-regulated genes the fell into the annotation categories with a statistical significance, P-value (Fisher's Exact Test). (GENOMATIX software v.2 (http://www.genomatix.de). (DOCX) [file pone.0032306.s005.docx]

**Table S2. Gene Ontology and Disease categories for significantly up- and down-regulated genes (by a fold change of ≥+/-2).**

**Up-regulated genes**

| **GO category description** | **Count in Category** | **P-value** |
| --- | --- | --- |
| Immune response | 42 | 1.37E-18 |
| Immune system process | 50 | 4-05E-18 |
| Apoptosis | 48 | 1.19E-15 |
| Programmed cell death | 48 | 1.61E-15 |
| Response to wounding | 35 | 1.95E-15 |
| Cell death | 50 | 2.17E-15 |
| Death | 50 | 2.46E-15 |
| Defense response | 37 | 3.78E-15 |
| Inflammatory response | 26 | 1.50E-13 |
| Regulation of apoptosis | 39 | 1.63E-13 |
| **Disease (MeSH term )** | **Count in Category** | **P-value** |
| Inflammation | 110 | 4.01E-28 |
| Respiratory Hypersensitivity | 68 | 3.72E-23 |
| Granuloma | 42 | 4.58E-23 |
| Atherosclerosis | 61 | 4.84E-23 |
| Lung Diseases, Obstructive | 71 | 6.40E-23 |
| Asthma | 64 | 9.74E-23 |
| Pneumococcal infections | 36 | 7.02E-22 |
| Shock, Septic | 44 | 1.35E-21 |
| Hypersensitivity, Immediate | 73 | 1.72E-21 |
| Systemic inflammatory Response Syndrome | 46 | 2.79E-21 |

**Down-regulated genes**

| **GO category description** | **Count in Category** | **P-value** |
| --- | --- | --- |
| Lung development | 3 | 4.23E-04 |
| Respiratory tube development | 3 | 4.60E-04 |
| Lung morphogenesis | 2 | 6.00E-04 |
| Embryonic development | 5 | 1.53E-03 |
| Signaling | 12 | 1.92E-03 |
| Signaling pathway | 10 | 2.02E-03 |
| Regulation of cellular process | 16 | 2.21E-03 |
| Negative regulation of transcription by TF localization | 1 | 2.92E-03 |
| Gall bladder development | 1 | 2.92E-03 |
| Calcium ion import | 1 | 2.92E-03 |
| **Disease (MeSH term )** | **Count in Category** | **P-value** |
| Cystadenocarcinoma, serous | 4 | 5.76E-03 |
| Genital diseases, female | 11 | 7.76E-03 |
| Genital neoplasms, female | 10 | 9.13E-03 |
