# Supplementary material for: Combined Chromatin and Expression Analysis Reveals Specific Regulatory Mechanisms within Cytokine Genes in the Macrophage Early Immune Response
Source: PLoS One. 2012 Feb 27;7(2):e32306. doi: 10.1371/journal.pone.0032306 (PMC3288078; doi:10.1371/journal.pone.0032306)
Supplement: Table S3 — Replicated H3Ac peaks in biological replicates. (DOCX) [file pone.0032306.s006.docx]

**Table S3. Replicated H3Ac peaks in biological replicates**

|  | **Number of H3Ac**  **Peaks** | **Replicated H3Ac**  **peaks**^*^ | **Common H3Ac**  **peaks**^*^ |
| --- | --- | --- | --- |
| Unstimulated sample 1 | 17,458 | 14,704 | 11,834 |
| Unstimulated sample 2 | 18,882 |  |  |
| LPS-stimulated sample 1 | 13,223 | 12,113 |  |
| LPS-stimulated Sample 2 | 14,610 |  |  |

^*^ Replicated peaks and common peaks have to fulfil the condition of 1 bp minimum overlapping position between peaks and samples.
