# Supplementary material for: Combined Chromatin and Expression Analysis Reveals Specific Regulatory Mechanisms within Cytokine Genes in the Macrophage Early Immune Response
Source: PLoS One. 2012 Feb 27;7(2):e32306. doi: 10.1371/journal.pone.0032306 (PMC3288078; doi:10.1371/journal.pone.0032306)
Supplement: Table S4 — In silico transcription binding search in unique S5P RNAPII peaks. Upper table summarize the in silico TF search for unique S5P RNAPII peaks (either −LPS or LPS+) that OVERLAP with H3Ac peaks. Bottom table shows the results of the in silico TF search for unique S5P RNAPII peaks (either −LPS or LPS+) NOT overlapping H3Ac peaks. Z-score represents the distance from the population mean in units of the population standard deviation. (Genomatix (Matrix Library Version 8.4). (DOCX) [file pone.0032306.s007.docx]

**Table S4. *In silico* transcription binding search in unique S5P RNAPII peaks**.

| *In silico* TF search for unique S5P RNAPII peaks (either –LPS or LPS+) that OVERLAP with H3Ac peaks | | |
| --- | --- | --- |
| Top 10 TF matrices (shorted by Z-score of overrepresentation against the genome) | | TF family |
| NRF1.01 | Nuclear respiratory factor 1 | NRF1 |
| ZF5.02 | ZF5 POZ domain zinc finger, zinc finger protein 161 | ZF5F |
| E2F2.01 | E2F transcription factor 2 | E2FF |
| E2F3.01 | E2F transcription factor 3 | E2FF |
| HDBP1_2.01 | Huntington's disease gene regulatory region-binding protein 1 and 2 (SLC2A4 regulator and papillomavirus binding factor) | HDBP |
| SP1.03 | Stimulating protein 1, ubiquitous zinc finger transcription factor | SP1F |
| SP1.02 | Stimulating protein 1, ubiquitous zinc finger transcription factor | SP1F |
| EGR1.02 | EGR1, early growth response 1 | EGRF |
| CTCF.01 | CCCTC-binding factor | CTCF |
| ZF5.01 | Zinc finger / POZ domain transcription factor | ZF5F |

Z-score= the distance from the population mean in units of the population standard deviation. (Genomatix (Matrix Library Version 8.4)

| *In silico* TF search for unique S5P RNAPII peaks (either –LPS or LPS+) NOT overlapping H3Ac peaks | | |
| --- | --- | --- |
| Top 10 TF matrices (shorted by Z-score of overrepresentation against the genome) | | TF family |
| TIEG.01 | TGFbeta-inducible early gene (TIEG) / Early growth response gene alpha (EGRalpha) | SP1F |
| EGR2.01 | Egr-2/Krox-20 early growth response gene product | EGRF |
| MYF5.01 | Myf5 myogenic bHLH protein | MYOD |
| NRF1.01 | Nuclear respiratory factor 1 | NRF1 |
| NGFIC.01 | Nerve growth factor-induced protein C | EGRF |
| ZNF202.01 | Transcriptional repressor, binds to elements found predominantly in genes that participate in lipid metabolism | ZF02 |
| ASCL2.01 | Achaete-scute complex homolog-like 2 | NEUR |
| HEN1.02 | HEN1 | HAND |
| TCFAP2B.01 | Transcription factor AP-2, beta | AP2F |
| RREB1.01 | Ras-responsive element binding protein 1 | RREB |

Z-score= the distance from the population mean in units of the population standard deviation. Genomatix (Matrix Library Version 8.4)
