# Supplementary material for: Combined Chromatin and Expression Analysis Reveals Specific Regulatory Mechanisms within Cytokine Genes in the Macrophage Early Immune Response
Source: PLoS One. 2012 Feb 27;7(2):e32306. doi: 10.1371/journal.pone.0032306 (PMC3288078; doi:10.1371/journal.pone.0032306)
Supplement: Table S5 — Summary of the combined analysis of H3Ac, S5P RNAPII and Sp1 ChIP-seq peaks and their locations. (DOCX) [file pone.0032306.s008.docx]

**Table S5. Summary of the combined analysis of H3Ac, S5P RNAPII and Sp1 ChIP-seq peaks and their locations**.

| **H3Ac ChIP-seq peaks co-localized with S5P RNAPII and Sp1 ChIP-seq peaks** | | | | | |
| --- | --- | --- | --- | --- | --- |
|  | **-LPS** | **+LPS** | **+/-LPS common peaks** | **-LPS unique peaks** | **+LPS unique peaks** |
| **Number of peaks** | 2,581 | 3,258 | 1,690 | 891 | 1,576 |
| **% Located in promoter** | 97.9% | 98% | 98.3% | 97.2% | 97.5% |
| **High CpG island content peaks** | 2,511 | 3,182 | 1,650 | 861 | 1,537 |
| **% Located in promoter** | 98% | 98% | 98,3% | 97.4% | 97.7% |
| **Low CpG island content peaks** | 70 | 76 | 40 | 30 | 39 |
| **% Located in promoter** | 94.3% | 94% | 97.5% | 90% | 89.7% |
