# Supplementary material for: Combined Chromatin and Expression Analysis Reveals Specific Regulatory Mechanisms within Cytokine Genes in the Macrophage Early Immune Response
Source: PLoS One. 2012 Feb 27;7(2):e32306. doi: 10.1371/journal.pone.0032306 (PMC3288078; doi:10.1371/journal.pone.0032306)
Supplement: Table S6 — Summary of the integration analysis of ChIP-seq data and expression profile in macrophages after LPS. Tables show gene name and chromosome position (Chr.Pos); P-value (Student's t-test); fold change due to LPS stimulation; [P-value and fold change: – no available expression data] TSS position, annotated transcription start site (hg19); H3AC common peak: presence (YES) or absence (NO) of a significant H3Ac peaks in promoter (−2 Kb/+1 Kb of TSS) for both +/−LPS conditions.; the effect of LPS, Peak extension; direction of chromatin expansion (→, upstream only; ←, downstream only; -, no expansion); S5P RNAPII & Sp1 peaks: presence of a significant peak in unstimulated condition (−LPS), LPS treated cells (+LPS); common in both conditions (+/−LPS) or absence in either condition (NO) in promoters (−2 Kb/+1 Kb of TSS); CpG island presence or absence in promoter (−2 Kb/+1 Kb of TSS); TATA box in silico finding (−150 bp/+50 bp of TSS). RED BOLD: 105 genes included in MeSH inflammatory category. Tables are divided into gene families and classified groups. (DOC) [file pone.0032306.s009.doc]

**Table S6. Summary of the integration analysis of ChIP-seq data and expression profile in macrophages after LPS**

1. CC Chemokines & Receptors
2. CXC Chemokines & Receptors
3. C Chemokine & Receptors
4. C3XC Chemokine & Receptors
5. Interleukins & Receptors
6. Interferon & Receptors
7. Up-regulated Inflammation elated Genes
8. Down-regulated Inflammation Related Genes
9. **CC Chemokine family**

| **Gene** | **Chr. Pos** | | | **P-value** | **Fold change** | **TSS**  **position** | | **Common H3Ac peak** | **Peak extension** | | **S5P RNAPII**  **peak** | **Sp1**  **peak** | **CpG**  **Island** | | **TATA**  **Box** | |
| --- | --- | --- | --- | --- | --- | --- | --- | --- | --- | --- | --- | --- | --- | --- | --- | --- |
| ***CCL1*** | | | 17q12 | 3.50E-02 | 3.10 | 32,691,253 | | NO | - | | NO | NO | NO | | NO | |
| *CCL2* | | | 17q12 | 5.20E-02 | 2.18 | 32,582,296 | | NO | - | | NO | NO | NO | | NO | |
| *CCL3* | | | 17q12 | -- | -- | 34,417,506 | | NO | - | | +/- LPS | NO | NO | | YES | |
| ***CCL4*** | | | 17q12 | 5.20E-04 | 29.59 | 34,431,220 | | NO | - | | + LPS | NO | NO | | YES | |
| *CCL5* | | | 17q12 | 2.10E-02 | 1.46 | 34,207,377 | | NO | - | | +/- LPS | NO | NO | | YES | |
| *CCL7* | | | 17q12 | -- | -- | 32,597,240 | | NO | - | | NO | NO | NO | | YES | |
| *CCL8* | | | 17q12 | 0.140 | 3.85 | 32,646,066 | | NO | - | | NO | NO | NO | | NO | |
| *CCL11* | | | 17q12 | -- | -- | 32,612,687 | | NO | - | | NO | NO | NO | | NO | |
| *CCL13* | | | 17q12 | -- | -- | 32,683,471 | | NO | - | | NO | NO | NO | | YES | |
| *CCL14* | | | 17q12 | -- | -- | 34,313,764 | | NO | - | | NO | NO | NO | | NO | |
| *CCL15* | | | 17q12 | -- | -- | 34,329,100 | | NO | - | | NO | NO | NO | | YES | |
| *CCL16* | | | 17q12 | -- | -- | 34,308,523 | | NO | - | | NO | NO | NO | | NO | |
| *CCL17* | | | 16q13 | -- | -- | 57,438,679 | | NO | - | | NO | NO | NO | | NO | |
| ***CCL18*** | | | 17q2 | 9.62E-05 | 2.76 | 34,391,643 | | NO | - | | NO | NO | NO | | YES | |
| *CCL19* | | | 9p13.3 | -- | -- | 34,691,274 | | NO | - | | NO | NO | NO | | NO | |
| ***CCL20*** | | | 2q36 | 4.50E-03 | 5.97 | 228,678,558 | | NO | - | | + LPS | NO | NO | | YES | |
| *CCL21* | | | 9p13.3 | -- | -- | 34,710,147 | | NO | - | | NO | NO | NO | | NO | |
| ***CCL22*** | | | 16q13 | 4.80E-02 | 2.18 | 57,392,718 | | NO | - | | NO | NO | NO | | NO | |
| *CCL23* | | | 17q12 | -- | -- | 34,345,005 | | NO | - | | NO | NO | NO | | YES | |
| *CCL24* | | | 7q11.23 | 0.555 | 1.27 | 75,443,092 | | NO | - | | NO | NO | NO | | NO | |
| *CCL25^#^* | | | 19p13.2 | -- | -- | 8,117,884 | | YES | → | | NO | NO | NO | | NO | |
|  | | |  |  |  | ^#^8,117,646 | | YES | → | | NO | NO | NO | | YES | |
| *CCL26* | | | 7q11.23 | -- | -- | 75,419,064 | | NO | - | | NO | NO | NO | | NO | |
| *CCL27^#^* | | | 9p13.3 | -- | -- | ^#^34,662,689 | | YES | ← | | NO | NO | NO | | NO | |
| *CCL28* | | 5p12 | | -- | -- | | 43,397,230 | YES | | ← | +/- LPS | NO | | YES | | NO |
|  | |  | |  |  | | 43,412,488 | NO | | - | NO | NO | | NO | | NO |

# within 2.5Kb analysis, these genes CCL25 and CCL27 behave as positive promoter for H3Ac,and CpG islands, respectively.

**CC Chemokines Receptors**

| **Gene** | **Chr. Pos** | **P-value** | **Fold**  **change** | **TSS**  **position** | **Common H3Ac peak** | **Peak extension** | **S5P RNAPII**  **peak** | **Sp1**  **peak** | **CpG**  **Island** | **TATA**  **Box** |
| --- | --- | --- | --- | --- | --- | --- | --- | --- | --- | --- |
| ***CCR1*** | 3p21.31 | 9.77E-03 | 2.14 | 46,249,832 | YES | → | NO | NO | NO | YES^Δ^ |
| *CCR2* | 3p21.31 | *--* | -- | 46,395,235 | NO | - | NO | NO | NO | NO |
| *CCR3* | 3p21.31 | *--* | -- | 46,283,688 | NO | - | NO | NO | NO | NO |
|  |  |  |  | 46,205,096 | NO | - | NO | NO | NO | NO |
| *CCR4* | 3p22.3 | *--* | -- | 32,993,066 | NO | - | NO | NO | NO | NO |
| *CCR5* |  | **--** | -- | 46,411,633 | NO | - | NO | NO | NO | NO |
| *CCR6* | 6q27 | *--* | -- | 167,536,241 | NO | - | NO | NO | NO | NO |
|  |  |  |  | 167,525,295 | NO | - | + LPS | NO | NO | NO |
|  |  |  |  | 167,412,816 | YES | - | +/- LPS | +/- LPS | YES | NO |
| *CCR7* | 17q21.2 | 5.30E-02 | 1.75 | 38,721,724 | NO | - | NO | NO | NO | NO |
| *CCR8* | 3p22.2 | **--** | -- | 39,371,197 | NO | - | NO | NO | NO | NO |
| *CCR9* | 3p21.31 | *--* | -- | 45,928,019 | NO | - | NO | NO | NO | NO |
| *CCR10* | 17q21.2 | *--* | -- | 40,833,845 | NO | - | NO | NO | YES | NO |

YES^Δ^ = *in silico* finding of TATA box motif downstream the 5´UTR

**2. CXC Chemokines**

| **Gene** | **Chr. Pos** | | | **P-value** | | **Fold**  **change** | | **TSS position** | **Common**  **H3Ac peak** | **Peak extension** | | **S5P RNAPII**  **peak** | | | | **Sp1**  **peak** | **CpG**  **Island** | | **TATA**  **Box** |
| --- | --- | --- | --- | --- | --- | --- | --- | --- | --- | --- | --- | --- | --- | --- | --- | --- | --- | --- | --- |
| ***CXCL1*** | | 4q21 | | | 5.30E-03 | | 14.09 | 74,735,109 | YES | ← | | | + LPS | NO | | | YES | | YES |
| ***CXCL2*** | | 4q21 | | | 1.94E-07 | | 24.61 | 74,965,998 | YES | ← | | | + LPS | NO | | | YES | | NO |
| ***CXCL3*** | | 4q21 | | | 1.80E-03 | | 5.10 | 74,905,491 | YES | - | | | +/- LPS | + LPS | | | YES | | NO |
| *CXCL5* | | 4q13.3 | | | -- | | -- | 74,864,416 | NO | - | | | NO | NO | | | YES | | NO |
| *CXCL6* | | 4q13.3 | | | -- | | -- | 74,702,273 | YES | - | | | NO | NO | | | YES | | NO |
| ***CXCL8=IL8*** | | 4q13-q21 | | | 1.80E-02 | | 3.36 | 74,606,275 | NO | - | | | +/- LPS | NO | | | NO | | YES |
| *CXCL9* | | 4q21.1 | | | -- | | -- | 76,928,641 | NO | - | | | NO | NO | | | NO | | NO |
| *CXCL10* | | 4q21.1 | | | 0.106 | | 3.12 | 76,944,650 | NO | - | | | NO | NO | | | NO | | NO |
| *CXCL11* | | 4q21.1 | | | 9.0E-02 | | 3.90 | 76,957,233 | NO | - | | | NO | NO | | | NO | | YES |
| *CXCL12* | | 10q11.22 | | | 0.39 | | 1.18 | 44,880,542 | NO | - | | | NO | NO | | | YES | | NO |
| *CXCL13* | | 4q21.1 | | | -- | | -- | 78,432,907 | NO | - | | | NO | NO | | | NO | | NO |
| *CXCL14* | | 5q31.1 | | | -- | | -- | 134,914,969 | NO | - | | | NO | NO | | | YES | | NO |
| *CXCL16* | | 17p13.2 | | | 0.61 | | 1.17 | 4,639,675 | NO | - | | | NO | NO | | | NO | | NO |
|  | |  | | |  | |  | 4,643,223 | YES | ↔ | | | - LPS | NO | | | YES | | NO |
| *CXCL17* | | 19q13.2 | | | -- | | -- | 42,947,136 | NO | - | | | NO | NO | | | NO | | NO |
|  | |  | | |  | |  |  |  |  | | |  |  | | |  | |  |
| **CXC Chemokine Receptors** | | | | | | | | | | | | | | | | | | |  |
| **Gene** | | | **Chr. Pos** | | **P-value** | **Fold**  **change** | | **TSS**  **position** | **Common**  **H3Ac peak** | | **Peak extension** | | **S5P RNAPII peak** | | **Sp1**  **peak** | | | **CpG Island** | **TATA Box** |
| *CXCR1=IL8RA* | | | 2q35 | | -- | -- | | 219,031,716 | NO | | - | | NO | | NO | | | NO | NO |
| *CXCR2=IL8RB* | | | 2q35 | | -- | -- | | 218,990,746 | NO | | - | | NO | | NO | | | NO | NO |
|  | | |  | |  |  | | 218,989,998 | NO | | - | | NO | | NO | | | NO | NO |
| *CXCR3* | | | Xq13.1 | | -- | -- | | 70,838,367 | NO | | - | | NO | | NO | | | NO | NO |
| *CXCR4* | | | 2q22.1 | | 0.655 | -1.23 | | 136,873,813 | YES | | - | | NO | | NO | | | YES | NO |
|  | | |  | |  |  | | 136,875,307 | YES | | - | | NO | | NO | | | YES | NO |
|  | | |  | |  |  | | 136,875,725 | YES | | - | | NO | | NO | | | YES | YES |
| *CXCR5* | | | 11q23.3 | | 0.201 | -1.63 | | 118,764,101 | NO | | - | | NO | | NO | | | NO | NO |
|  | | |  | |  |  | | 118754,541 | YES | | - | | NO | | NO | | | NO | NO |
| *CXCR6* | | | 3p21.31 | | -- | -- | | 45,986,541 | NO | | - | | NO | | NO | | | NO | NO |
|  | | |  | |  |  | | 45,984,973 | NO | | - | | NO | | NO | | | NO | NO |
| *CXCR7* | | | 2q37.3 | | -- | -- | | 237,478,380 | YES | | ↔ | | NO | | NO | | | YES | NO |
|  | | |  | |  |  | | 237,469,424 | NO | | - | | NO | | NO | | | NO | NO |

**3. C Chemokines & Receptors**

| **Gene** | **Chr. Pos** | **P-value** | **Fold change** | **TSS**  **position** | **Common H3Ac peak** | **Peak extension** | **S5P RNAPII peak** | **Sp1**  **peak** | **CpG Island** | **TATA Box** |
| --- | --- | --- | --- | --- | --- | --- | --- | --- | --- | --- |
| *XCL1* | 1q24.2 | -- | -- | 168,545,856 | NO | - | NO | NO | NO | YES |
| *XCL2* | 1q24.2 | **--** | **--** | 168,513,235 | NO | - | NO | NO | NO | YES |
| *XCR1* | 3p21.31 | -- | -- | 46,068,979 | NO | - | NO | NO | NO | NO |

**4. CX3C Chemokines & Receptor**s

| **Gene** | **Chr. Pos** | **P-value** | **Fold**  **change** | **TSS position** | **Common**  **H3Ac peak** | **Peak extension** | **S5P RNAPII peak** | **Sp1**  **peak** | **CpG Island** | **TATA Box** |
| --- | --- | --- | --- | --- | --- | --- | --- | --- | --- | --- |
| *CX3CL1* | 16q21 | -- | -- | 57,406,414 | NO | - | NO | NO | NO | YES |
| CX_3_CR1 | 3p22.2 | 7.10E-02 | -2.47 | 39,321,527 | NO | - | NO | NO | NO | NO |

**5. Interleukins**

| **Gene** | **Chr. Pos** | **P-value** | **Fold change** | **TSS position** | | **Common**  **H3Ac peak** | | **Peak extension** | **S5P RNAPII peak** | **Sp1**  **peak** | **CpG Island** | **TATA Box** |
| --- | --- | --- | --- | --- | --- | --- | --- | --- | --- | --- | --- | --- |
| ***IL1A*** | 2q14 | 1.20E-04 | 28.62 | 113,543,971 | | NO | | - | NO | NO | NO | YES |
| ***IL1B*** | 2q14 | 8.40E-03 | 2.12 | 113,594,356 | | YES | | ← | NO | NO | NO | YES |
| *IL1F5* | 2q13 | -- | -- | 113,816,215 | | NO | | - | NO | NO | NO | NO |
| *IL1F6* | 2q13 | -- | -- | 113763449 | | NO | | - | NO | NO | NO | YES |
| *IL1F7* | 2q13 | -- | -- | 113,670,548 | | NO | | - | NO | NO | NO | NO |
| *IL1F8* | 2q13 | -- | -- | 113810,440 | | NO | | - | NO | NO | NO | NO |
| *IL1F9* | 2q13 | -- | -- | 113,735,606 | | NO | | - | NO | NO | NO | NO |
| *IL1F10* | 2q13 | -- | -- | 113,825,547 | | NO | | - | NO | NO | NO | YES |
| *IL2* | 4q27 | -- | -- | 123,377,650 | | NO | | - | NO | NO | NO | YES |
| *IL3* | 5q31.1 | -- | -- | 131,396,347 | | NO | | - | NO | NO | NO | NO |
| *IL4* | 5q31.1 | -- | -- | 132,037,272 | | NO | | - | NO | NO | NO | NO |
| *IL5* | 5q31.1 | -- | -- | 131,879,214 | | NO | | - | NO | NO | NO | NO |
| *IL6* | 7p15.3 | -- | -- | 22,766,766 | | NO | | - | NO | NO | NO | NO |
|  |  |  |  | 22,765,503 | | NO | | - | NO | NO | NO | NO |
| *IL7* | 8q21.12 | -- | -- | 79,710,443 | | NO | | - | NO | NO | NO | NO |
|  |  |  |  | 79,717,758 | | YES | | ← | + LPS | NO | YES | NO |
| ***IL8=CXCL8*** | 4q13-q21 | 1.80E-02 | 3.36 | 74,606,275 | | NO | | - | +/- LPS | NO | NO | YES |
| *IL9* | 5q31.1 | -- | -- | 135,231,516 | | NO | | - | NO | NO | NO | YES |
| *IL10* | 1q32.1 | **--** | -- | 206,945,839 | | NO | | - | NO | NO | NO | YES |
| *IL11* | 19q13.42 | 0.371 | 1.08 | 55,881,814 | | YES | | - | NO | NO | YES | YES |
| *IL12A* | 3q25.33 | -- | -- | 159,706,629 | | NO | | - | NO | NO | YES | NO |
| IL12B | 5q33.3 | -- | -- | 158,757,481 | | NO | | - | NO | NO | YES | NO |
| *IL13* | 5q31.1 | -- | -- | 131,993,865 | | NO | | - | NO | NO | YES | YES |
| *IL15* | 4q31.21 | -- | -- | 142,557,754 | | YES | | ↔ | NO | NO | YES | NO |
| *IL16* | 15q25.1 | 8.20E-02 | -1.10 | 81,591,721 | | NO | | - | NO | NO | NO | YES^Δ^ |
|  |  |  |  | 81,589,269 | | NO | | - | NO | NO | NO | NO |
|  |  |  |  | 81,587,467 | | NO | | - | NO | NO | NO | NO |
|  |  |  |  | 81,584,265 | | NO | | - | NO | NO | NO | YES |
|  |  |  |  | 81,571,158 | | NO | | - | NO | NO | NO | YES |
|  |  |  |  | 81,489,219 | | NO | | - | NO | NO | NO | NO |
|  |  |  |  | 81,480,694 | | NO | | - | NO | NO | NO | NO |
|  |  |  |  | 81,475,093 | | NO | | - | NO | NO | NO | NO |
| *IL17A* | 6p12.2 | -- | -- | 52,051,185 | | NO | | - | NO | NO | NO | YES |
|  |  |  |  | *Interleukins (continuation)* | | | |  |  |  |  |  |
| **Gene** | **Chr. Pos** | **P-value** | **Fold change** | **TSS position** | | **Common**  **H3Ac peak** | | **Peak extension** | **S5P RNAPII peak** | **Sp1**  **peak** | **CpG Island** | **TATA Box** |
| *IL17B* | 5q32 | 0.488 | 1.11 | 148,758,838 | | NO | | - | NO | NO | NO | NO |
| *IL17C^#^* | 16q24.3 | 0.285 | 1.08 | #88,705,001 | | YES | | ↔ | NO | NO | NO | NO |
| *IL17D* | 13q12.11 | 0.712 | 1.03 | 21,277,482 | | YES | | → | - LPS | NO | YES | NO |
| *IL17F* | 6p12.2 | -- | -- | 52,103,965 | | NO | | - | NO | NO | NO | NO |
|  |  |  |  | 52,109,298 | | NO | | - | NO | NO | NO | YES |
| *IL18* | 11q23.1 | 0.224 | 1.39 | 112,021,796 | | NO | | - | NO | NO | NO | NO |
|  |  |  |  | 112,034,840 | | NO | | - | NO | NO | NO | NO |
| *IL19* | 1q32.1 | -- | -- | 207,010,006 | | NO | | - | NO | NO | NO | NO |
|  |  |  |  | 207,002,222 | | NO | | - | NO | NO | NO | NO |
|  |  |  |  | 206,972,215 | | NO | | - | NO | NO | NO | NO |
| *IL20* | 1q32.1 | -- | -- | 207,039,154 | | NO | | - | NO | NO | NO | NO |
|  |  |  |  | 207,038,699 | | NO | | - | NO | NO | NO | YES |
| *IL21* | 4q27 | -- | -- | 123,542,211 | | NO | | - | NO | NO | NO | NO |
| *IL22* | 12q15 | -- | -- | 68,647,385 | | NO | | - | NO | NO | NO | YES |
| *IL23* | Heterodimeric cytokine consisting of two subunits IL23A & IL12B | | | | | | | | | | |  |
| ***IL23A*** | 12q13.3 | 4.42E-06 | 17.87 | 56,732,663 | | | NO | - | + LPS | NO | NO | YES |
| *IL24* | 1q32.1 | -- | -- | 207,070,789 | | | NO | - | NO | NO | NO | YES |
| *IL25* | 14q11.2 | -- | -- | 23,842,018 | | | NO | - | NO | NO | NO | NO |
| *IL26* | 12q15 | -- | -- | 68,619,571 | | | NO | - | NO | NO | NO | YES |
| *IL27* | 16p11.2 | -- | -- | 28,518,155 | | | NO | - | NO | NO | NO | NO |
| *IL27B=EBI3* | 19p13.3 | 5.36E-03 | 2.98 | 4,229,540 | | | NO | - | + LPS | NO | NO | NO |
| *IL28A=IFNL2* | 19q13.2 | -- | -- | 39,759,157 | | | NO | - | NO | NO | NO | NO |
| *IL28B=IFNL3* | 19q13.2 | -- | -- | 39,735,609 | | | NO | - | NO | NO | NO | NO |
| *IL29=IFNL1* | 19q13.2 | -- | -- | 39,786,965 | | | NO | - | NO | NO | NO | NO |
| *IL31* | 12q24.31 | -- | -- | | 122,658,746 | | NO | - | NO | NO | NO | NO |
| *IL32^#^* | 16p13.3 | 0.949 | 1.01 | | 3,115,785 | | NO | - | NO | NO | NO | NO |
|  |  |  |  | | 3,115,663 | | NO | - | NO | NO | NO | YES |
|  |  |  |  | | 3,115,612 | | NO | - | NO | NO | NO | YES^Δ^ |
|  |  |  |  | | *^#^*3,115,313 | | NO | - | NO | NO | NO | NO |
| *IL33* | 9p24.1 | -- | -- | | 6,241,684 | | NO | - | NO | NO | NO | NO |
|  |  |  |  | | 6,215,807 | | NO | - | NO | NO | NO | NO |
| *IL34* | 16q22.1 | 0.146 | -1.11 | | 70,680,468 | | NO | - | NO | NO | NO | NO |
| # within 5Kb and 6.5Kb analysis, these genes have a positive signal for common H3AC peak, and CpG islands, respectively. Δ *In silico* TATA box motif finded downstream the TSS | | | | | | | | | | | |  |

**Interleukin Receptors**

| **Gene** | | **Chr. Pos** | **P-value** | | | **Fold change** | | **TSS position** | **Common H3Ac peak** | **Peak extension** | | **S5P RNAPII peak** | | **Sp1**  **peak** | | | **CpG Island** | | **TATA Box** |
| --- | --- | --- | --- | --- | --- | --- | --- | --- | --- | --- | --- | --- | --- | --- | --- | --- | --- | --- | --- |
| ***IL1R1*** | | 2q12 | 4.58E-02 | | | 2.43 | | 102,770,401 | NO | - | | NO | | NO | | | NO | | YES |
|  | |  |  | | |  | | 102,759,246 | YES | → | | - LPS | | NO | | | YES | | NO |
|  | |  |  | | |  | | 102,686,836 | NO | - | | NO | | NO | | | NO | | NO |
| *IL1R2* | | 2q11.2 | 0.781 | | | 1.06 | | 102,624,977 | NO | - | | NO | | NO | | | NO | | NO |
|  | |  |  | | |  | | 102,615,459 | NO | - | | NO | | NO | | | NO | | NO |
|  | |  |  | | |  | | 102,608,306 | NO | - | | NO | | NO | | | NO | | YES |
| *IL1RL1* | | 2q12.1 | -- | | | -- | | 102,953,717 | NO | - | | NO | | NO | | | NO | | YES |
|  | |  |  | | |  | | 102,927,962 | NO | - | | NO | | NO | | | NO | | YES^Δ^ |
| *IL1RL2* | | 2q12.1 | -- | | | -- | | 102,803,433 | NO | - | | NO | | NO | | | YES | | NO |
| *IL2RA* | | 10p15.1 | -- | | | -- | | 6,067,988 | NO | - | | NO | | NO | | | NO | | NO |
|  | |  |  | | |  | | 6,104,272 | NO | - | | NO | | NO | | | NO | | NO |
| *IL2RB* | | 22q12.3 | -- | | | -- | | 37,545,962 | NO | - | | NO | | NO | | | NO | | NO |
| *IL2RG* | | Xq13.1 | 0.719 | | | 1.14 | | 70,329,240 | NO | - | | NO | | NO | | | NO | | NO |
|  | |  |  | | |  | | 70,331,403 | NO | - | | NO | | NO | | | NO | | NO |
| *IL3RA* | | Xp22.33 | -- | | | -- | | 1,460,621 | NO | - | | NO | | NO | | | NO | | NO |
|  | |  |  | | |  | | 1,455,509 | NO | - | | NO | | NO | | | NO | | NO |
| *IL4R* | | 16p12.1 | 0.231 | | | 1.13 | | 27,325,251 | YES | ↔ | | +/- LPS | | NO | | | YES | | NO |
| *IL5RA* | | 3p26.2 | 0.758 | | | 1.01 | | 3,134,046 | NO | - | | NO | | NO | | | NO | | NO |
|  | |  |  | | |  | | 3,146,671 | NO | - | | NO | | NO | | | NO | | NO |
|  | |  |  | | |  | | 3,152,058 | NO | - | | NO | | NO | | | NO | | NO |
| *IL6R* | | 1q21.3 | -- | | | -- | | 154,377,669 | YES | - | | NO | | NO | | | YES | | NO |
| ***IL7R*** | | 5p13 | 3.60E-04 | | | 5.13 | | 35,856,991 | NO | - | | NO | | NO | | | NO | | YES |
| *IL8RA* | | 2q35 | -- | | | -- | | 219,031,716 | NO | - | | NO | | NO | | | NO | | NO |
| *IL8RB* | | 2q35 | -- | | | -- | | 218,990,746 | NO | - | | NO | | NO | | | NO | | NO |
|  | |  |  | | |  | | 218,989,998 | NO | - | | NO | | NO | | | NO | | NO |
| *IL9R* | | Xq28 | -- | | | -- | | 155,227,246 | NO | - | | NO | | NO | | | NO | | NO |
| *IL10RA* | | 11q23.3 | 0.120 | | | 2.03 | | 117,857,106 | YES | ← | | +/- LPS | | + LPS | | | YES | | NO |
| *IL11RA^#^* | | 9p13.3 | 0.166 | | | -1.12 | | 34,653,932 | NO | - | | NO | | NO | | | NO | | NO |
|  | |  |  | | |  | | 34,652,182 | NO | - | | NO | | NO | | | NO | | NO |
|  | |  |  | | |  | | *^#^*34,650,699 | YES | → | | NO | | NO | | | NO | | NO |
| *IL12RB1* | | 19p13.11 | 0.854 | | | -1.03 | | 18,197,742 | NO | - | | NO | | NO | | | NO | | NO |
|  | |  |  | | |  | | 18,209,626 | YES | ↔ | | NO | | + LPS | | | YES | | NO |
|  | |  |  | | |  | |  |  |  | |  | |  | | |  | |  |
|  |  | | |  | *Interleukins receptors (continuation)* | | | | | | | |  | |  |  | |  | |
| **Gene** | **Chr. Pos** | | | **P-value** | **Fold change** | | **TSS position** | | **Common**  **H3Ac peak** | | **Peak extension** | | **S5P RNAPII peak** | | **Sp1**  **peak** | **CpG Island** | | **TATA Box** | |
| *IL12RB2* | | 1p31.1 | 1.3E-02 | | | 1.62 | | 67,786,016 | NO | - | | NO | | NO | | | NO | | NO |
|  | |  |  | | |  | | 67,773,047 | YES | → | | NO | | NO | | | YES | | NO |
| *IL13RA1* | | Xq24 | 0.915 | | | 1.03 | | 117,861,559 | YES | ↔ | | +/- LPS | | + LPS | | | YES | | NO |
| *IL13RA2* | | Xq23 | -- | | | -- | | 114,252,207 | NO | - | | NO | | NO | | | NO | | NO |
| *IL15RA* | | 10p15.1 | 0.204 | | | 1.45 | | 6,002,530 | NO | - | | NO | | NO | | | NO | | NO |
|  | |  |  | | |  | | 6,019,537 | YES | - | | + LPS | | NO | | | YES | | NO |
|  | |  |  | | |  | | 6,020,142 | YES | - | | + LPS | | NO | | | YES | | NO |
| *IL17RA* | | 22q11.1 | 3.10E-02 | | | -1.55 | | 17,565,849 | YES | → | | +/- LPS | | NO | | | YES | | NO |
| *IL17RB* | | 3p21.1 | -- | | | -- | | 53,880,577 | YES | ↔ | | NO | | NO | | | YES | | YES |
| *IL17RC* | | 3p25.3 | 0.767 | | | -1.03 | | 9,970,712 | NO | - | | NO | | NO | | | NO | | NO |
|  | |  |  | | |  | | 9,958,764 | YES | → | | - LPS | | NO | | | YES | | NO |
| *IL17RD* | | 3p14.3 | -- | | | -- | | 57,176,603 | NO | - | | NO | | NO | | | NO | | NO |
|  | |  |  | | |  | | 57,199,403 | YES | ↔ | | NO | | NO | | | YES | | NO |
|  | |  |  | | |  | | 57,204,331 | NO | - | | NO | | - LPS | | | YES | | NO |
| *IL17RE* | | 3p25.3 | 0.897 | | | 1.00 | | 9,944,303 | NO | - | | NO | | NO | | | NO | | NO |
| ***IL18R1****^#^* | | 2q12 | 8.13E-05 | | | 5.62 | | *^#^*102,979,093 | NO | - | | NO | | NO | | | NO | | YES |
|  | |  |  | | |  | | 102,927,962 | NO | - | | NO | | NO | | | NO | | YES^Δ^ |
| *IL20RA* | | 6q23.3 | -- | | | -- | | 137,330,001 | NO | - | | NO | | NO | | | NO | | NO |
|  | |  |  | | |  | | 137,365,411 | YES | ↔ | | + LPS | | NO | | | YES | | NO |
|  | |  |  | | |  | | 137,366,298 | YES | ↔ | | + LPS | | NO | | | YES | | NO |
| *IL20RB* | | 3q22.3 | -- | | | -- | | 136,676,707 | NO | - | | NO | | NO | | | NO | | NO |
| *IL21R* | | 16p12.1 | 0.803 | | | -1.06 | | 27,438,579 | NO | - | | NO | | NO | | | NO | | NO |
|  | |  |  | | |  | | 27,413,723 | YES | - | | NO | | NO | | | NO | | NO |
| *IL22RA1* | | 1p36.11 | -- | | | -- | | 24,463,799 | NO | - | | NO | | NO | | | NO | | NO |
|  | |  |  | | |  | | 24,469,611 | NO | - | | NO | | NO | | | NO | | NO |
| *IL22RA2* | | 6q23.3 | -- | | | -- | | 137,494,785 | NO | - | | NO | | NO | | | NO | | NO |
| *IL23R* | | 1p31.3 | **--** | | | -- | | 67,673,299 | NO | - | | NO | | NO | | | NO | | NO |
|  | |  |  | | |  | | 67,648,519 | NO | - | | NO | | NO | | | NO | | NO |
|  | |  |  | | |  | | 67,635,025 | NO | - | | NO | | NO | | | NO | | YES^Δ^ |
|  | |  |  | | |  | | 67,632,169 | NO | - | | NO | | NO | | | NO | | NO |
|  | |  |  | | |  | | 67,604,590 | NO | - | | NO | | NO | | | NO | | NO |
| *IL27RA* | | 19p13.12 | 0.394 | | | -1.13 | | 14,142,262 | YES | ↔ | | UNTS | | NO | | | YES | | NO |
| *IL28RA=IFNLR1* | | 1p36.11 | -- | | | -- | | 24,513,751 | NO | - | | NO | | NO | | | YES | | NO |
| *IL31RA* | | 5q11.2 | -- | | | -- | | 55,149,150 | NO | - | | NO | | NO | | | NO | | NO |
|  | |  |  | | |  | | 55,147,334 | NO | - | | NO | | NO | | | NO | | YES |

# within 5Kb analysis, This gene behave as positive promoter for H3AC and CpG islands, respectively. YES^Δ^ = *in silico* finding of TATA box motif downstream the 5´UTR

**6. Interferons**

| **Gene** | **Chr. Pos** | **P-value** | **Fold change** | | | **TSS position** | **Common H3Ac peak** | **Peak extension** | **S5P RNAPII peak** | **Sp1 peak** | **CpG Island** | **TATA Box** |
| --- | --- | --- | --- | --- | --- | --- | --- | --- | --- | --- | --- | --- |
| *IFNA1* | 9p21.3 | -- | | -- | 21,440,440 | | NO | - | NO | NO | NO | NO† |
| *IFNA2* | 9p21.3 | -- | | -- | 21,385,396 | | NO | - | NO | NO | NO | NO† |
| *IFNA4* | 9p21.3 | -- | | -- | 21,187,598 | | NO | - | NO | NO | NO | NO† |
| *IFNA5* | 9p21.3 | -- | | -- | 21,305,255 | | NO | - | NO | NO | NO | NO |
| *IFNA6* | 9p21.3 | -- | | -- | 21,350,886 | | NO | - | NO | NO | NO | NO† |
| *IFNA7* | 9p21.3 | -- | | -- | 21,202,204 | | NO | - | NO | NO | NO | NO† |
| *IFNA8* | 9p21.3 | -- | | -- | 21,409,146 | | NO | - | NO | NO | NO | YES |
| *IFNA10* | 9p21.3 | -- | | -- | 21,207,142 | | NO | - | NO | NO | NO | NO† |
| *IFNA13* | 9p21.3 | -- | | -- | 21,368,075 | | NO | - | NO | NO | NO | NO† |
| *IFNA14* | 9p21.3 | -- | | -- | 21,239,978 | | NO | - | NO | NO | NO | NO† |
| *IFNA16* | 9p21.3 | -- | | -- | 21,217,310 | | NO | - | NO | NO | NO | NO† |
| *IFNA17* | 9p21.3 | -- | | -- | 21,228,221 | | NO | - | NO | NO | NO | NO† |
| *IFNA21* | 9p21.3 | -- | | -- | 21,166,659 | | NO | - | NO | NO | NO | NO† |
| *IFNB1* | 9p21.3 | -- | | -- | 21,077,943 | | NO | - | NO | NO | NO | YES |
| *IFNE* | 9p21.3 | -- | | -- | 21,482,312 | | NO | - | NO | NO | NO | YES^Δ^ |
| *IFNK* | 9p21.2 | -- | | -- | 27,524,312 | | NO | - | NO | NO | NO | YES |
| *IFNW1* | 9p21.3 | -- | | -- | 21,142,144 | | NO | - | NO | NO | NO | YES |
| *IFNG* | 12q15 | -- | | -- | 68,553,521 | | NO | - | NO | NO | NO | YES |
| *IFNL1=IL29* | 19q13.2 | **--** | | -- | 39,786,965 | | NO | - | NO | NO | NO | NO |
| *IFNL2=IL28A* | 19q13.2 | -- | | -- | 39,759,157 | | NO | - | NO | NO | NO | NO |
| *IFNL3=IL28B* | 19q13.2 | -- | | -- | 39,735,609 | | NO | - | NO | NO | NO | NO |

*NO†: IN SILICO FINDING TATA BOX (AVIAN TATA BOX)matrix similarity= 0.903* cactattTAAGacctat

**Interferon Receptors**

| **Gene** | **Chr. Pos** | **P-value** | **Fold change** | **TSS position** | **Common H3Ac peak** | **Peak extension** | **S5P RNAPII peak** | **Sp1**  **peak** | **CpG Island** | **TATA**  **Box** |
| --- | --- | --- | --- | --- | --- | --- | --- | --- | --- | --- |
| *IFNAR1* | 21q22.11 | 0.95 | 1.01 | 34,697,214 | YES | → | +/-LPS | NO | YES | NO |
|  |  |  |  | 34,696,734 | YES | → | +/-LPS | NO | YES | NO |
| *IFNAR2* | 21q22.11 | 3.2E-03 | 1.40 | 34,617,798 | NO | - | NO | NO | NO | NO |
|  |  |  |  | 34,602,231 | YES | ↔ | +/-LPS | LPS | YES | NO |
| *IFNGR1* | 6q23.3 | 1.7E-02 | -1.34 | 137,539,651 | YES | → | +/-LPS | LPS | YES | NO |
|  |  |  |  | 137,540,567 | YES | → | +/-LPS | LPS | YES | NO |
| *IFNGR2* | 21q22.11 | 9.3E-04 | 1.48 | 34,787,186 | NO | - | NO | NO | NO | NO |
|  |  |  |  | 34,775,202 | YES | ↔ | +/-LPS | NO | YES | NO |
| *IL10RB* | 21q22.11 | 0.977 | -1.00 | 34,640,233 | YES | ← | +/-LPS | NO | YES | NO |
|  |  |  |  | 34,638,672 | YES | ← | +/-LPS | NO | YES | NO |
|  |  |  |  | 34,621,014 | NO | - | NO | NO | NO | NO |
| *IFNLR1=IL28RA* | 1p36.11 | -- | -- | 24,513,751 | NO | - | NO | NO | YES | NO |

**8.Up-regulated Inflammatory Related Genes**

| **Gene** | **Chr. Pos** | **P-value** | **Fold change** | **TSS position** | **Common H3Ac peak** | **Peak extension** | **S5P RNAPII peak** | **Sp1**  **peak** | **CpG Island** | **TATA Box** |
| --- | --- | --- | --- | --- | --- | --- | --- | --- | --- | --- |
| *ABTB2* | 11p13 | 1.39E-02 | 2.86 | 34,378,573 | YES | ↔ | + LPS | -LPS | YES | NO |
| *ACSL1* | 4q35.1 | 1,00E-04 | 2.57 | 185,687,910 | NO | - | NO | NO | NO | NO |
|  |  |  |  | 185726942 | NO | - | + LPS | NO | NO | NO |
|  |  |  |  | 185733613 | NO | - | NO | NO | NO | YES |
|  |  |  |  | 185,747,215 | YES | - | +/- LPS | NO | YES | NO |
| ***ADORA2A^#^*** | 22q11.23 | 1.07E-04 | 4.90 | 24,829,099 | NO | - | NO | NO | NO | NO |
|  |  |  |  | 24,828,087 | NO | - | NO | NO | NO | NO |
|  |  |  |  | 24,827,819 | NO | - | NO | NO | NO | NO |
|  |  |  |  | #24,823,530 | YES | - | NO | NO | NO | NO |
|  |  |  |  | 24,813,709 | NO | - | NO | NO | NO | NO |
| *ARL5B* | 10p12.31 | 1.06E-02 | 2.56 | 18,948,313 | YES | ↔ | +/- LPS | NO | YES | NO |
| *ARRDC3* | 5q14 | 7.43E-03 | 2.00 | 90,679,149 | YES | → | +/- LPS | NO | YES | NO |
| ***ATP1B1*** | 1q24.2 | 1.41E-03 | 2.13 | 169,075,947 | YES | ← | +/- LPS | NO | YES | NO |
| ***ATP2B1*** | 12q21.33 | 2.82E-03 | 2.95 | 90,020,392 | NO | - | NO | NO | NO | YES |
|  |  |  |  | 90,049,844 | NO | - | NO | NO | NO | NO |
| *B3GNT5* | 3q27.1 | 9.94E-03 | 2.08 | 182,983,090 | NO | - | NO | NO | NO | NO |
|  |  |  |  | 182,971,606 | YES | ↔ | + LPS | NO | YES | NO |
|  |  |  |  | 182,971,032 | YES | ↔ | + LPS | NO | YES | NO |
| *B4GALT5* | 20q13.13 | 2.31E-03 | 2.39 | 48,330,421 | YES | ↔ | +/- LPS | NO | YES | NO |
| ***BCL2A1*** | 15q25.1 | 3.32E-04 | 2.76 | 80,263,643 | NO | - | NO | NO | NO | YES^Δ^ |
| ***BIRC3*** | 11q22.2 | 1.27E-03 | 13.10 | 102,188,194 | YES | ↔ | + LPS | NO | YES | NO |
| ***BTG2*** | 1q32.1 | 1.12E-02 | 2.99 | 203,274,664 | YES | ↔ | +/- LPS | NO | YES | NO |
| *C6ORF145* | 6p25.2 | 6.36E-04 | 4.84 | 37,52,246 | YES | - | +/- LPS | NO | YES | NO |
| *C13ORF29* | 13q34 | 3.91E-02 | 2.60 | 111,522,655 | NO | - | +/- LPS | NO | NO | NO |
| *CBR3* | 21q22.12 | 3.19E-03 | 2.09 | 37,507,263 | YES | ↔ | + LPS | NO | YES | NO |
| *CCRN4L* | 4q31.1 | 2.04E-04 | 3.24 | 139,936,943 | YES | → | +/- LPS | NO | YES | NO |
| ***CD40*** | 2q13.12 | 2.15E-04 | 3.66 | 44,746,906 | YES | ↔ | + LPS | + LPS | YES | NO |
| ***CD44*** | 11p13 | 1.80E-03 | 3.18 | 35,211,382 | NO | - | NO | NO | NO | NO |
|  |  |  |  | 35,160,417 | YES | ↔ | +/- LPS | NO | YES | NO |
| ***CD69*** | 12p13.31 | 8.74E-06 | 5.11 | 9,804,764 | NO | - | NO | NO | NO | YES |
| ***CD83*** | 6p23 | 1.85E-03 | 4.43 | 14,117,865 | YES | → | +/- LPS | NO | YES | NO |
| ***CD274*** | 9p24.1 | 1.89E-02 | 5.48 | 5,456,100 | NO | - | NO | NO | NO | NO |
|  |  |  |  | 5,450,559 | NO | - | +/- LPS | + LPS | YES | NO |
| *Up-regulated inflammatory related genes (continuation)* | | | | | | | | | | |
| **Gene** | **Chr. Pos** | **P-value** | **Fold change** | **TSS position** | **Common H3Ac peak** | **Peak extension** | **S5P RNAPII peak** | **Sp1**  **peak** | **CpG Island** | **TATA Box** |
| ***CFLAR*** | 2q33.1 | 8.01E-04 | 3.49 | 202,004,998 | NO | - | NO | NO | NO | NO |
|  |  |  |  | 201,997,707 | NO | - | NO | NO | NO | NO |
|  |  |  |  | 201,994,452 | NO | - | NO | NO | NO | NO |
|  |  |  |  | 201,983,387 | YES | → | +/- LPS | NO | YES | NO |
|  |  |  |  | 201,980,816 | YES | → | +/- LPS | NO | YES | NO |
| ***CLDN1*** | 3q28 | 3.72E-02 | 2.57 | 190,023,490 | YES | ↔ | NO | NO | YES | NO |
| *CNKSR3* | 6q25.2 | 1.32E-02 | 2.05 | 154,831,753 | YES | → | + LPS | + LPS | YES | NO |
| *CRIM1* | 2p22.2 | 1.07E-02 | 2.17 | 36,436,874 | YES | - | NO | NO | YES | NO |
| ***CSF1*** | 1p13.3 | 4.59E-02 | 2.27 | 110,453,233 | YES | → | - LPS | NO | YES | NO |
| ***CTGF*** | 6q23.2 | 2.93E-04 | 9.40 | 132,272,518 | YES | ↔ | + LPS | NO | YES | YES |
| ***DDIT4*** | 10q22.1 | 3.91E-03 | 3.10 | 74,033,677 | YES | - | - LPS | + LPS | YES | NO |
| *DENND5A* | 11p15.4 | 1.69E-02 | 2.87 | 9,193,792 | NO | - | NO | NO | NO | NO |
|  |  |  |  | 9,286,871 | YES | ← | + LPS | NO | YES | NO |
| ***DLL4*** | 15q15.1 | 2.02E-02 | 3.83 | 41,221,547 | YES | → | +/- LPS | NO | YES | YES |
| *DRAM1* | 12q23.2 | 1.87E-02 | 2.46 | 102,271,105 | YES | ↔ | +/- LPS | + LPS | YES | NO |
| ***DUSP1*** | 5q35.1 | 3.82E-03 | 5.78 | 172,197,497 | YES | → | - LPS | + LPS | YES | NO |
|  |  |  |  | 172,197,634 | YES | → | - LPS | + LPS | YES | NO |
|  |  |  |  | 172,198,203 | YES | → | - LPS | + LPS | YES | YES |
| ***DUSP2*** | 2q11.2 | 4.86E-03 | 2.66 | 96,811,179 | YES | → | +/- LPS | + LPS | YES | NO |
| *DUSP16* | 12p13.2 | 5.26E-05 | 2.27 | 12,715,317 | YES | - | + LPS | NO | YES | NO |
| *E2F7* | 12q21.2 | 8.28E-04 | 3.32 | 77,424,112 | NO | - | NO | NO | NO | NO |
|  |  |  |  | 77,459,360 | YES | → | + LPS | NO | YES | NO |
| *EHD1* | 11q13.1 | 5.26E-05 | 5.53 | 64,624,124 | YES | → | NO | NO | NO | NO |
|  |  |  |  | 64,646,191 | YES | → | +/- LPS | + LPS | YES | NO |
|  |  |  |  | 64,647,149 | YES | → | +/- LPS | + LPS | YES | NO |
| ***EREG*** | 4q13.3 | 4,68E-02 | 2.80 | 75,230,860 | NO | - | NO | NO | NO | YES |
| ***ETS2*** | 21q22.2 | 9.6E-04 | 2.49 | 40,177,849 | YES | - | +/- LPS | + LPS | YES | NO |
|  |  |  |  | 40,177,231 | YES | - | +/- LPS | + LPS | YES | NO |
| ***ETV3*** | 1q23.1 | 7.21E-03 | 2.55 | 157,108,383 | YES | ↔ | +/- LPS | NO | YES | NO |
| ***F3*** | 1p21.3 | 3.25E-03 | 2.12 | 95,007,371 | YES | - | - LPS | + LPS | YES | NO |
| *FAM49A* | 2p24.2 | 2.86E-03 | 2.42 | 16,804,344 | NO | - | NO | NO | NO | NO |
|  |  |  |  | 16,847,096 | YES | ← | NO | NO | NO | NO |
| ***FOSL2*** | 2p23.2 | 4.00E-04 | 3.05 | 28,615,779 | YES | ↔ | - LPS | NO | YES | NO |
| ***GADD45A*** | 1p31.3 | 4.83E-03 | 2.45 | 68,150,883 | YES | ← | NO | NO | YES | NO |
| **Gene** | **Chr. Pos** | **P-value** | **Fold change** | **TSS position** | **Common H3Ac peak** | **Peak extension** | **S5P RNAPII peak** | **Sp1**  **peak** | **CpG Island** | **TATA Box** |
| *GBP2* | 1p22.2 | 2.84E-04 | 4.91 | 89,591,799 | NO | - | NO | NO | NO | NO |
| *GBP3* | 1p22.2 | 2.90E-02 | 2.10 | 89,488,549 | NO | - | NO | NO | NO | NO |
| ***GCH1*** | 14q22.2 | 6.21E-04 | 3.28 | 55,369,542 | YES | ↔ | +/- LPS | + LPS | YES | YES |
| *GEM* | 8q22.1 | 1.67E-02 | 4.24 | 95,274,557 | YES | - | +/- LPS | + LPS | YES | NO |
| *GFPT2* | 5q35.3 | 2.18E-02 | 2.40 | 179,780,315 | YES | - | NO | + LPS | YES | NO |
| ***GIMAP5*** | 7q36.1 | 3.44E-04 | 2.77 | 150,434,451 | YES | ← | NO | NO | NO | NO |
| ***GPR132*** | 14q32.33 | 6.10E-06 | 4.54 | 105,522,503 | YES | ↔ | NO | NO | NO | NO |
|  |  |  |  | 105,531,754 | YES | ↔ | - LPS | NO | NO | NO |
| ***GPR84*** | 12q13.3 | 1.20E-02 | 2.01 | 54,758,258 | NO | - | NO | NO | NO | NO |
| *GRAMD3* | 5q23.2 | 2.25E-06 | 7.36 | 125,695,788 | NO | - | NO | NO | NO | NO |
|  |  |  |  | 125,707,123 | NO | - | NO | NO | NO | NO |
|  |  |  |  | 125,758,869 | YES | - | +/- LPS | + LPS | YES | NO |
|  |  |  |  | 125,798,037 | NO | - | NO | NO | NO | NO |
|  |  |  |  | 125,800,787 | NO | - | NO | NO | NO | NO |
| *HIVEP1* | 6p24.1 | 3.54E-03 | 2.21 | 12,012,724 | YES | - | NO | NO | YES | NO |
| *HIVEP2* | 6q24.2 | 2.38E-03 | 3.86 | 143,266,338 | YES | ↔ | NO | NO | YES | NO |
| ***ICAM1*** | 19p13.2 | 8.44E-04 | 4.97 | 10,381,517 | YES | ↔ | +/- LPS | NO | YES | NO |
| *ICAM4* | 19p13.2 | 2.74E-03 | 3.02 | 10,397,650 | YES | ↔ | NO | NO | YES | NO |
| ***IER3*** | 6p21.23 | 1.40E-03 | 5.46 | 30,820,306 | YES | - | +/- LPS | + LPS | YES | NO |
| ***IRAK2*** | 3p25.3 | 5.01E-04 | 5.17 | 10,206,563 | YES | ↔ | +/- LPS | NO | YES | NO |
| ***ITGB8*** | 7p21.1 | 5.82E-03 | 3.01 | 20,370,725 | YES | → | + LPS | NO | YES | NO |
| *JDP2* | 14q24.3 | 5.20E-04 | 2.69 | 75,895,060 | YES | ← | NO | NO | YES | NO |
|  |  |  |  | 75,894,509 | YES | ← | NO | NO | YES | NO |
|  |  |  |  | 75,894,920 | YES | ← | NO | NO | YES | NO |
|  |  |  |  | 75,898,837 | NO | - | NO | NO | NO | NO |
| *JUNB* | 19p13.2 | 1.27E-03 | 2.16 | 12,902,310 | YES | ↔ | - LPS | NO | YES | YES |
| ***KANK1*** | 9p24.3 | 2.94E-03 | 2.05 | 734,748 | NO | - | NO | NO | NO | NO |
|  |  |  |  | 732,378 | NO | - | NO | NO | NO | NO |
|  |  |  |  | 706,896 | YES | - | +/- LPS | NO | YES | NO |
|  |  |  |  | 686,641 | NO | - | NO | NO | NO | NO |
|  |  |  |  | 684,194 | NO | - | NO | NO | NO | NO |
|  |  |  |  | 569,461 | NO | - | NO | NO | NO | NO |
|  |  |  |  | 547,148 | NO | - | NO | NO | NO | NO |
|  |  |  |  | 504,703 | YES | - | NO | + LPS | YES | NO |
|  |  |  |  | 470,294 | YES | ↔ | - LPS | NO | YES | NO |
| *Up-regulated inflammatory related genes (continuation)* | | | | | | | |  |  |  |
| **Gene** | **Chr. Pos** | **P-value** | **Fold change** | **TSS position** | **Common H3Ac peak** | **Peak extension** | **S5P RNAPII peak** | **Sp1**  **peak** | **CpG Island** | **TATA Box** |
| ***KCNA3*** | 1p13.3 | 4.36E-06 | 4.44 | 111,217,655 | YES | - | NO | NO | YES | NO |
| *KCNN2* | 5q22.3 | 5.15E-04 | 2.72 | 113,698,016 | YES | → | - LPS | NO | YES | YES |
|  |  |  |  | 113,769,227 | NO |  | NO | NO | NO | NO |
| ***KLF6*** | 10p15.1 | 2.94E-02 | 2.29 | 3,827,473 | YES | → | +/- LPS | +/- LPS | YES | NO |
| *KLF7* | 2q33.3 | 1.27E-04 | 2.21 | 208,030,614 | YES | ↔ | +/- LPS | + LPS | YES | NO |
| *KYNU* | 2q22.2 | 6.80E-04 | 3.71 | 143,635,195 | NO | - | NO | NO | NO | YES |
| ***LAMP3*** | 3q27.1 | 3.87E-04 | 11.53 | 182,880,667 | YES | ↔ | + LPS | NO | YES | NO |
| ***LCP2*** | 5q35.1 | 1.56E-02 | 2.24 | 169,724,822 | YES | ↔ | + LPS | NO | NO | NO |
| ***LIF*** | 22q12 | 7.14E-04 | 10.86 | 30,642,796 | NO | - | NO | NO | YES | YES^Δ^ |
| *LONRF1* | 8p23.1 | 5.48E-03 | 2.16 | 12,594,649 | NO | - | NO | NO | NO | NO |
|  |  |  |  | 12,600,791 | NO | - | NO | NO | NO | YES |
|  |  |  |  | 12,612,992 | YES | ↔ | +/- LPS | + LPS | YES | NO |
| ***MAP3K8*** | 10p11.23 | 2.37E-04 | 3.08 | 30,727,751 | NO | - | NO | NO | NO | YES |
|  |  |  |  | 30,722,866 | YES | ← | +/- LPS | + LPS | YES | NO |
| *MARCKS* | 6q22.1 | 5.78E-04 | 3.05 | 114,178,527 | YES | ← | NO | + LPS | NO | NO |
| *MCOLN2* | 1p22.3 | 9.65E-05 | 4.37 | 85,462,796 | YES | → | + LPS | NO | YES | NO |
| *MFSD2A* | 1p34.2 | 2.83E-04 | 2.57 | 40,420,784 | YES | → | NO | +/- LPS | YES | NO |
| *MOP-1* | 4q21.21 | 2.18E-02 | 5.44 | 82,390,474 | YES | - | NO | NO | NO | NO |
| *MSC* | 8q13.3 | 1.01E-02 | 2.67 | 72,756,731 | YES | ↔ | - LPS | NO | YES | NO |
| *MST4* | Xq26.2 | 8.19E-05 | 2.97 | [131,157,536](http://genome.ucsc.edu/cgi-bin/hgc?hgsid=203660707&g=htcGeneInGenome&i=uc004ewm.1&c=chrX&l=131157535&r=131209971&o=knownGene&table=knownGene) | YES | → | +/- LPS | + LPS | YES | NO |
|  |  |  |  | 131,157,245 | YES | → | +/- LPS | + LPS | YES | NO |
| *N4BP2L1* | 13q13.1 | 3.77E-03 | 2.34 | 33,002,315 | YES | ↔ | +/- LPS | + LPS | YES | NO |
| ***NAMPT*** | 7q22.3 | 6.80E-03 | 2.31 | 105,925,638 | YES | → | +/- LPS | +/- LPS | YES | NO |
| *NAV3* | 12q21.2 | 2.81E-02 | 2.19 | 78,511,807 | NO | - | NO | NO | NO | YES |
|  |  |  |  | 78,430,643 | NO | - | NO | NO | NO | NO |
|  |  |  |  | 78,225,069 | NO | - | NO | NO | NO | NO |
| ***NFE2L2*** | 2q31.2 | 1.3E-04 | 2.46 | 178,128,617 | YES | ↔ | +/- LPS | + LPS | YES | NO |
|  |  |  |  | 178,129,859 | YES | ↔ | +/- + LPS | + LPS | YES | NO |
|  |  |  |  | 178,257,419 | YES | → | +/- LPS | NO | YES | NO |
| ***NFKB1*** | 4q24 | 2.00E-04 | 3.35 | 103,422,486 | YES | - | +/- LPS | NO | YES | NO |
|  |  |  |  | 103,498,871 | YES | - | NO | NO | NO | NO |

|  |  | *Up-regulated inflammatory related genes (continuation)* | | | | | | |  |  |  | |
| --- | --- | --- | --- | --- | --- | --- | --- | --- | --- | --- | --- | --- |
| **Gene** | **Chr. Pos** | **P-value** | **Fold change** | **TSS position** | **Common H3Ac peak** | **Peak extension** | **S5P RNAPII peak** | **Sp1**  **peak** | **CpG Island** | **TATA Box** |  | |
| ***NFKB2*** | 10q24.32 | 2.0E-03 | 2.46 | 104,155,500 | YES | ↔ | NO | +/- LPS | YES | NO |  | |
|  |  |  |  | 104,155,432 | YES | ↔ | NO | +/- LPS | YES | NO |  | |
|  |  |  |  | 104,154,339 | YES | ↔ | NO | +/- LPS | YES | NO |  | |
|  |  |  |  | 104,154,229 | YES | ↔ | NO | NO | YES | NO |  | |
| ***NFKBIA*** | 14q13 | 1.42E-04 | 6.44 | 35,874,347 | YES | ↔ | +/- LPS | NO | YES | NO |  | |
| ***NFKBIZ*** | 3q12 | 2.85E-06 | 21.95 | 101,546,857 | NO | - | NO | NO | NO | YES |  | |
|  |  |  |  | 101,568,358 | YES | ↔ | +/- LPS | +/- LPS | YES | NO |  | |
| *NIACR1* | 12q24.31 | 1.06E-02 | 3.84 | 123,187,904 | NO | - | NO | NO | NO | NO |  | |
| *NIACR2* | 12q24.31 | 8.01E-04 | 8.78 | 121,767,392 | NO | - | NO | NO | NO | NO |  | |
| ***NINJ1*** | 9q22.31 | 1.18E-04 | 2.34 | 95,896,570 | YES | → | - LPS | + LPS | YES | NO |  | |
| ***NLRP3*** | 1q44 | 6.87E-04 | 3.46 | 247,581,355 | YES | ↔ | +/- LPS | NO | NO | NO |  | |
|  |  |  |  | 247,579,458 | YES | ↔ | +/- LPS | NO | NO | NO |  | |
| ***NR4A3*** | 9q22.33 | 7.33E-03 | 3.63 | 102,589,009 | YES | - | NO | NO | YES | NO |  | |
|  |  |  |  | 102,584,137 | YES | - | - LPS | NO | YES | NO |  | |
| *OGFRL1* | 6q13 | 2.11E-04 | 2.59 | 71,998,477 | YES | ↔ | +/- LPS | + LPS | YES | NO |  | |
| ***OLR1*** | 12p13.2 | 4.75E-04 | 3.47 | 10,320,198 | NO | - | NO | NO | NO | NO |  | |
|  |  |  |  | 10,324,790 | NO | - | NO | NO | NO | NO |  | |
| ***PDE4B*** | 1p31.3 | 1.11E-04 | 3.69 | 66,820,070 | NO | - | NO | NO | NO | NO |  | |
|  |  |  |  | 66,797,793 | NO | - | NO | NO | NO | NO |  | |
|  |  |  |  | 66,458,390 | NO | - | NO | NO | NO | NO |  | |
|  |  |  |  | 66,258,193 | YES | ← | + LPS | NO | YES | NO |  | |
| ***PDP1*** | 8q22.1 | 1.69E-02 | 2.00 | 94,929,992 | YES | → | + LPS | + LPS | YES | NO |  | |
|  |  |  |  | 94,929,175 | YES | → | + LPS | + LPS | YES | NO |  | |
|  |  |  |  | 94,929,083 | YES | → | + LPS | + LPS | YES | NO |  | |
| *PDSS1* | 10p12.1 | 4.26E-03 | 2.04 | 27,023,464 | NO | - | NO | NO | NO | NO |  | |
|  |  |  |  | 26,986,595 | YES | ↔ | +/- LPS | NO | YES | NO |  | |
| ***PELI1*** | 2p14 | 1.20E-02 | 3.28 | 64,325,364 | NO | - | NO | NO | NO | NO |  | |
|  |  |  |  | 64,339,483 | NO | - | NO | NO | NO | NO |  | |
|  |  |  |  | 64,371,605 | YES | ← | +/- LPS | + LPS | YES | NO |  | |
| *PERP* | 6q23.3 | 2.55E-03 | 2.00 | 138,428,660 | YES | ↔ | NO | NO | YES | NO |  | |
| ***PIM1*** | 6p21.2 | 1.29E-02 | 2.16 | 37,140,233 | YES | ← | +/- LPS | NO | YES | NO |  | |
|  |  |  |  | 37,137,922 | YES | ← | +/- LPS | +/- LPS | YES | NO |  | |
| *Up-regulated inflammatory related genes (continuation)* | | | | | | | | | | |  |  |
| **Gene** | **Chr. Pos** | **P-value** | **Fold change** | **TSS position** | **Common H3Ac peak** | **Peak extension** | **S5P RNAPII peak** | **Sp1**  **peak** | **CpG Island** | **TATA Box** |  | |
| ***PIM2*** | Xp11.23 | 1.21E-02 | 2.33 | 48,776,413 | YES | - | +/- LPS | + LPS | YES | NO |  | |
| *PLAGL2* | 20q11.21 | 7.562E-05 | 2.54 | 30,795,546 | YES | ↔ | +/- LPS | +/- LPS | YES | NO |  | |
| ***PLAU*** | 10q22.2 | 4.57E-05 | 4.73 | 75,671,283 | YES | - | NO | NO | YES | NO |  | |
|  |  |  |  | 75,670,918 | YES | - | NO | NO | YES | NO |  | |
|  |  |  |  | 75,670,918 | YES | - | NO | NO | YES | NO |  | |
| ***PLEK*** | 2p14 | 9.81E-04 | 2.02 | 68,592,322 | YES | → | +/- LPS | NO | NO | YES |  | |
| *PLEKHF2* | 8q22.1 | 4.11E-03 | 2.99 | 96,146,038 | YES | → | +/- LPS | + LPS | YES | NO |  | |
| *PLK3* | 1p34.1 | 4.47E-03 | 2.85 | 45,266,036 | YES | → | +/- LPS | + LPS | YES | NO |  | |
| ***PMAIP1*** | 18q21.32 | 8.83E-03 | 3.68 | 57,567,192 | YES | ↔ | +/- LPS | + LPS | YES | NO |  | |
| ***PNRC1*** | 6q15 | 1.28E-04 | 3.29 | 89,791,555 | YES | ↔ | +/- LPS | +/- LPS | YES | NO |  | |
|  |  | 1.28E-04 | 3.29 | 89,790,429 | YES | ↔ | +/- LPS | +/- LPS | YES | NO |  | |
| ***PPP1R15A*** | 19q13.33 | 4.11E-03 | 3.13 | 49,375,649 | YES | ↔ | +/- LPS | NO | YES | YES |  | |
| ***PSTPIP2*** | 18q21.1 | 4.14E-03 | 3.03 | 43,652,250 | YES | ↔ | + LPS | NO | YES | NO |  | |
| ***PTGER2*** | 14q22 | 2.40E-02 | 2.63 | 52781016 | YES | -- | NO | + LPS | YES | NO |  | |
| ***PTGER4*** | 5p13.1 | 2.80E-03 | 2.76 | 40680032 | YES | ← | +/- LPS | + LPS | YES | NO |  | |
| ***PTGS2*** | 1q25 | 5.30E-04 | 26.32 | 186,650,560 | YES | ← | +/- LPS | NO | YES | YES |  | |
| ***PTX3*** | 3q25.32 | 1.08E-03 | 7.10 | 157,154,580 | YES | → | +/- LPS | NO | YES | NO |  | |
| *PVRL3* | 3q13.13 | 2.35E-02 | 2.87 | 110,790,865 | YES | → | NO | NO | YES | NO |  | |
| *RASGEF1B* | 4q21.21 | 3.35E-02 | 2.82 | 82,393,061 | YES | - | +/- LPS | +/- LPS | YES | YES^Δ^ |  | |
| *RASL11A* | 13q12.2 | 1.99E-03 | 3.53 | 27,844,464 | YES | ↔ | +/- LPS | NO | YES | NO |  | |
| ***RASSF5*** | 1q32.1 | 1.32E-03 | 2.40 | 206,756,601 | NO | - | NO | NO | NO | NO |  | |
|  |  |  |  | 206,730,492 | YES | ← | - LPS | NO | YES | NO |  | |
|  |  |  |  | 206,680,879 | YES | ← | - LPS | + LPS | YES | NO |  | |
| *RAP2C* | Xq26.2 | 2.13E-05 | 2.42 | 131,352,189 | YES | - | +/- LPS | NO | YES | NO |  | |
| ***REL*** | 2p16.1 | 5.26E-04 | 5.17 | 61,108,752 | YES | ← | +/- LPS | +/- LPS | YES | NO |  | |
| *RHOF* | 12q24.31 | 5.85E-03 | 2.00 | 122,231,594 | YES | → | NO | + LPS | YES | NO |  | |
| ***RIPK2*** | 8q21.3 | 8.72E-04 | 3.79 | 90,769,975 | YES | ↔ | +/- LPS | NO | YES | NO |  | |
| *RNF144B* | 6p22.3 | 1.08E-03 | 6.82 | 18,387,594 | NO | - | + LPS | NO | YES | YES |  | |
| *RNF19B* | 1p35.1 | 1.17E-03 | 5.11 | 33,430,286 | YES | ↔ | +/- LPS | - LPS | YES | NO |  | |
| *RPL35* | 9q33.3 | 3.40E-02 | 2.40 | 127,624,240 | YES | ↔ | +/- LPS | NO | YES | NO |  | |
| ***SAT1*** | Xp22.11 | 3.27E-03 | 2.33 | 23,801,275 | YES | → | +/- LPS | NO | YES | NO |  | |
| ***SDC4*** | 20q13.12 | 5.60E-04 | 3.14 | [43,977,064](http://genome.ucsc.edu/cgi-bin/hgTracks?hgsid=197586389&db=hg19&position=chr20%3A43953929-43977064) | YES | ↔ | +/- LPS | NO | YES | NO |  | |
| ***SERPINB2*** | 18q21.33 | 2.88E-02 | 3.05 | 61,554,939 | NO | - | NO | NO | NO | YES |  | |
|  |  |  |  | 61,564,325 | NO | - | NO | NO | NO | YES |  | |
| *Up-regulated inflammatory related genes (continuation)* | | | | | | | | | | |  | |
| **Gene** | **Chr. Pos** | **P-value** | **Fold change** | **TSS position** | **Common H3Ac peak** | **Peak extension** | **S5P RNAPII peak** | **Sp1**  **peak** | **CpG Island** | **TATA Box** |  | |
| ***SERPINB9*** | 6p25.2 | 1.93E-02 | 2.63 | 2,892,367 | NO | - | NO | NO | YES | NO |  | |
|  |  |  |  | 2,903,545 | NO | - | + LPS | NO | YES | YES |  | |
| ***SERPINE2*** | 2q36.1 | 3.41E-03 | 5.46 | 224,896,195 | NO | - | NO | NO | NO | NO |  | |
|  |  |  |  | 224,903,357 | YES | ↔ | + LPS | NO | YES | NO |  | |
|  |  |  |  | 224,904,036 | YES | ↔ | + LPS | NO | YES | NO |  | |
| *SDC4* | 20q13.12 | 5.63E-04 | 3.14 | [43,977,064](http://genome.ucsc.edu/cgi-bin/hgTracks?hgsid=197586389&db=hg19&position=chr20%3A43953929-43977064) | YES | ↔ | +/- LPS | NO | YES | NO |  | |
| ***SLC39A14*** | 8p21.3 | 5.56E-03 | 2.19 | 22,225,050 | YES | ↔ | +/- LPS | + LPS | YES | NO |  | |
|  |  |  |  | 22,224,762 | YES | ↔ | +/- LPS | + LPS | YES | NO |  | |
| *SLC43A2* | 17p13.3 | 1.16E-03 | 2.04 | 1,508,211 | NO | - | NO | NO | YES | NO |  | |
|  |  |  |  | 1,531,544 | YES | ↔ | NO | NO | YES | NO |  | |
|  |  |  |  | 1,531,635 | YES | ↔ | NO | NO | YES | NO |  | |
|  |  |  |  | 1,532,130 | YES | ↔ | NO | NO | YES | YES |  | |
| ***SLC7A2*** | 8p22 | 8.03E-03 | 2.04 | 17,400,827 | NO | - | NO | NO | NO | NO |  | |
|  |  |  |  | 17,396,286 | NO | - | NO | NO | NO | NO |  | |
|  |  |  |  | 17,354,600 | YES | - | + LPS | + LPS | YES | NO |  | |
| ***SLAMF7*** | 1q23.3 | 2.91E-02 | 2.47 | 160,709,077 | NO | - | NO | NO | NO | NO |  | |
| ***SOCS3*** | 17q25.3 | 5.83E-07 | 3.84 | 76,356,158 | YES | ↔ | +/- LPS | + LPS | YES | NO |  | |
| ***SOD2*** | 6q25.3 | 4.42E-04 | 6.40 | 160,114,353 | YES | ↔ | +/- LPS | + LPS | YES | NO |  | |
| *ST20* | 15q25.1 | 2.36E-03 | 2.00 | 80,207,598 | NO | - | NO | NO | NO | NO |  | |
|  |  |  |  | 80,216,077 | YES | ↔ | +/- LPS | + LPS | YES | NO |  | |
| ***STAT4*** | 2q32.3 | 2.24E-02 | 2.51 | 191,934,497 | NO | - | NO | NO | NO | NO |  | |
|  |  |  |  | 192,015,925 | NO | - | + LPS | NO | NO | NO |  | |
| ***STAT5A*** | 17q21.2 | 1.17E-03 | 3.41 | 40,458,134 | NO | - | NO | NO | NO | YES |  | |
|  |  |  |  | 40,440,074 | YES | ↔ | - LPS | NO | YES | NO |  | |
|  |  |  |  | 40,439,885 | YES | ↔ | - LPS | NO | YES | NO |  | |
|  |  |  |  | 40,439,565 | YES | ↔ | - LPS | NO | YES | NO |  | |
| ***STX11*** | 6q24.2 | 4.43E-03 | 3.93 | 144,471,654 | YES | ↔ | +/- LPS | + LPS | YES | NO |  | |
| *TBC1D10A* | 22q12.2 | 4.57E-03 | 2.59 | 30,722,894 | YES | ← | NO | + LPS | YES | NO |  | |
| *TIFA* | 4q25 | 4.07E-03 | 3.42 | 113,207,059 | YES | ↔ | +/- LPS | NO | YES | NO |  | |
| ***TNF*** | 6p21.3 | 6.87E-06 | 47.23 | 31,543,350 | YES | ↔ | - LPS | NO | NO | YES |  | |
| ***TNFAIP2*** | 14q32 | 4.46E-05 | 13.37 | 103,592,664 | YES | ↔ | NO | NO | YES | NO |  | |
|  |  |  |  | 103,599,087 | YES |  | NO | NO | NO | NO |  | |
| ***TNFAIP3*** | 6q23 | 1.09E-05 | 9.15 | 138,188,581 | YES | → | +/- LPS | +/- LPS | YES | NO |  | |
| ***TNFAIP6*** | 2q23.3 | 1.27E-04 | 19.21 | 152,214,105 | NO | - | NO | NO | NO | NO |  | |
| *Up-regulated inflammatory related genes (continuation)* | | | | | | | | | | |  | |
| **Gene** | **Chr. Pos** | **P-value** | **Fold change** | **TSS position** | **Common H3Ac peak** | **Peak extension** | **S5P RNAPII peak** | **Sp1**  **peak** | **CpG Island** | **TATA Box** |  | |
| ***TNFAIP8*** | 5q23.1 | 1.23E-03 | 4.19 | 118,604,418 | YES | ← | +/- LPS | +/- LPS | YES | NO |  | |
|  |  |  |  | 118,668,870 | NO | - | NO | NO | NO | NO |  | |
|  |  |  |  | 118,691,596 | YES | → | + LPS | NO | YES | NO |  | |
| ***TNFRSF9*** | 1p36.23 | 1.76E-02 | 2.18 | 8,000,887 | NO | - | NO | NO | YES | NO |  | |
| ***TNFSF9*** | 19p13.3 | 1.39E-02 | 2.07 | 6,531,010 | YES | → | +/- LPS | NO | YES | YES |  | |
| ***TNIP1*** | 5q33.1 | 6.56E-04 | 3.69 | 150,443,308 | NO | - | NO | NO | NO | NO |  | |
|  |  |  |  | 150,444,688 | NO | - | NO | NO | NO | NO |  | |
|  |  |  |  | 150,460,997 | YES | - | +/- LPS | +/- LPS | YES | NO |  | |
|  |  |  |  | 150,466,719 | NO | - | NO | NO | NO | NO |  | |
| *TNIP2* | 4p16.3 | 2.08E-03 | 2.73 | 2,757,752 | YES | ↔ | +/- LPS | NO | YES | NO |  | |
|  |  |  |  | 2,758,103 | YES | ↔ | +/- LPS | NO | YES | NO |  | |
| ***TP53BP2*** | 1q41 | 1.34E-04 | 2.20 | 224,033,674 | YES | ↔ | +/- LPS | + LPS | YES | NO |  | |
| *TP53INP1* | 8q22.1 | 1.67E-03 | 2.62 | 95,938,200 | YES | - | NO | +/- LPS | YES | NO |  | |
| ***TRAF1*** | 9q33.2 | 1.12E-04 | 15.49 | 123,676,850 | NO | -- | NO | NO | NO | NO |  | |
|  |  |  |  | 123,689,173 | YES | → | +/- LPS | NO | YES | NO |  | |
|  |  |  |  | 123,691,480 | YES | → | - LPS | NO | YES | NO |  | |
| *TRIP10* | 19p13.3 | 1.22E-02 | 2.53 | 6,739,707 | YES | ↔ | + LPS | +/- LPS | YES | NO |  | |
| ***TWIST1*** | 7p21.1 | 1.78E-04 | 2.62 | 19,157,295 | YES | ↔ | + LPS | NO | YES | YES |  | |
| *TXNDC11* | 16p13.13 | 5.43E-04 | 2.71 | 11,8366,48 | YES | - | +/- LPS | +/- LPS | YES | YES^Δ^ |  | |
| *UNQ9364* | 6p23 | 1.54E-02 | 8.45 | 14,124,115 | NO | - | NO | NO | NO | NO |  | |
| *UNQ9368* | 4q35.1 | 1.08E-02 | 7.57 | 185,719,451 | NO | - | NO | NO | NO | NO |  | |
| *USP12* | 13q12.13 | 2.41E-03 | 2.07 | 27,746,029 | YES | → | + LPS | +/- LPS | YES | NO |  | |
| *USP53* | 4q26 | 7.82E-04 | 2.23 | 120,133,782 | YES | ↔ | +/- LPS | + LPS | YES | NO |  | |
| *WT1* | 11p13 | 3.36E-02 | 2.09 | 32,457,087 | YES | ↔ | - LPS | + LPS | YES | NO |  | |
| *WTAP* | 6q25.3 | 5.662E-05 | 3.02 | 160,172,234 | NO | - | NO | NO | NO | NO |  | |
|  |  |  |  | 160,148,620 | YES | ↔ | +/- LPS | + LPS | YES | NO |  | |
|  |  |  |  | 160,148,129 | YES | ↔ | +/- LPS | + LPS | YES | NO |  | |
| ***XBP1*** | 22q12.1 | 7.84E-05 | 2.28 | 29,196,560 | YES | ↔ | +/- LPS | + LPS | YES | NO |  | |
| *ZBTB10* | 8q21.13 | 1.19E-06 | 4.20 | 81,398,448 | YES | - | + LPS | + LPS | YES | NO |  | |
|  |  |  |  | 81,397,854 | YES | - | + LPS | + LPS | YES | YES |  | |
| ***ZC3H12A*** | 1p34.3 | 7.91E-05 | 5.80 | 37,945,751 | YES | ↔ | NO | + LPS | NO | NO |  | |
|  |  |  |  | 37,940,119 | YES | ↔ | - LPS | + LPS | YES | YES^Δ^ |  | |

**9. Down-regulated Inflammation Related Genes**

| **Gene** | **Chr. Pos** | **P-value** | **Fold change** | **TSS position** | **Common**  **H3Ac peak** | **Peak extension** | **S5P RNAPII peak** | **Sp1**  **peak** | **CpG Island** | **TATA Box** |
| --- | --- | --- | --- | --- | --- | --- | --- | --- | --- | --- |
| *ARHGAP12* | 10p11.22 | 3.26E-02 | -2.27 | 32,197,854 | NO | - | NO | NO | NO | NO |
|  |  |  |  | 32,217,770 | YES | - | +/-LPS | NO | YES | NO |
| *ARHGEF3* | 3p14.3 | 1.68E-03 | -2.79 | 56,809,595 | NO | - | NO | NO | NO | NO |
|  |  |  |  | 56,835,995 | YES | - | +/-LPS | +/-LPS | YES | NO |
|  |  |  |  | 56,950,499 | NO | - | NO | NO | NO | NO |
|  |  |  |  | 57,113,336 | YES | ← | NO | +LPS | YES | NO |
| *ARL4C* | 2q37.1 | 4.82E-02 | -2.01 | 235,405,693 | NO | - | NO | NO | YES | NO |
| ***ATG16L1*** | 2q37.1 | 3.91E-03 | -2.74 | 234,160,217 | YES | - | +/-LPS | NO | YES | NO |
| *C16orf54* | 16p11.2 | 2.55E-03 | -2.45 | 29,757,340 | YES | ← | -LPS | NO | NO | NO |
| ***CEBPA*** | 19q13.11 | 1.10E-02 | -2.02 | 33,793,430 | YES | ↔ | +/-LPS | +LPS | YES | YES |
| ***CCR1*** | 3p21.31 | 9.77E-03 | -2.14 | 46,249,832 | YES | → | NO | NO | NO | YES^Δ^ |
| *FAM78A* | 9q34.13 | 1.85E-03 | -2.01 | 134,145,880 | YES | → | NO | NO | NO | NO |
|  |  | 1.85E-03 | -2.01 | 134,151,906 | YES | → | NO | NO | YES | NO |
| *FRAT1* | 10q24.1 | 2.20E-03 | -2.05 | 99,079,022 | YES | ↔ | NO | NO | YES | NO |
| ***GPR65*** | 14q31.3 | 1.92E-02 | -2.43 | 88,471,496 | NO | - | NO | NO | NO | YES |
| *HERPUD1* | 16q13 | 1.15E-04 | -2.01 | 56,969,863 | YES | ↔ | NO | NO | NO | YES |
|  |  | 1.15E-04 | -2.01 | 56,965,748 | YES | ↔ | +/-LPS | +LPS | YES | NO |
| *HHEX* | 10q23.33 | 1.39E-03 | -3.25 | 94,449,681 | YES | ← | -LPS | NO | YES | NO |
| *IMP3* | 15q24.2 | 5.27E-04 | -2.19 | 75,932,595 | YES | - | +/-LPS | -LPS | YES | NO |
|  |  |  |  | 75,941,047 | YES | ↔ | NO | +LPS | YES | NO |
| *LRRC33* | 3q29 | 5.39E-04 | -2.10 | 197,851,053 | YES | ↔ | NO | NO | YES | NO |
| *P2RY5= LPAR6* | 13q14.2 | 1.15E-04 | -2.98 | 48,987,653 | NO | - | NO | NO | NO | YES |
|  |  |  |  | 49,001,043 | NO | - | NO | NO | NO | YES |
|  |  |  |  | 49,018,840 | NO | - | NO | NO | NO | YES |
| *PDIK1L* | 1p26.11 | 5.06E-03 | -2.00 | 26,437,656 | YES | ↔ | -LPS | +/-LPS | YES | NO |
| *PDP2* | 16q22.1 | 3.98E-03 | -2.19 | 66,914,436 | YES | ↔ | +LPS | +/-LPS | YES | NO |
| *RNF168* | 3q29 | 3.97E-03 | -2.00 | 196,230,582 | YES | - | -LPS | +LPS | YES | NO |
| ***SLC30A1*** | 1q32.3 | 1.76E-02 | -2.00 | 211,752,099 | YES | - | LPS | +/-LPS | YES | NO |
| *SPRY2* | 13q31.1 | 4.11E-02 | -2.77 | 80,913,794 | YES | ↔ | NO | NO | YES | NO |
|  |  |  |  | 80,915,086 | YES | ↔ | NO | NO | YES | NO |

| *Down-regulated inflammation related genes (continuation)* | | | | | | | | | | |
| --- | --- | --- | --- | --- | --- | --- | --- | --- | --- | --- |
| **Gene** | **Chr. Pos** | **P-value** | **Fold change** | **TSS position** | **Common**  **H3Ac peak** | **Peak extension** | **S5P RNAPII peak** | **Sp1**  **peak** | **CpG Island** | **TATA Box** |
| *TIGD2* | 4q22.1 | 8.03E-03 | -2.00 | 90,033,968 | YES | - | -LPS | NO | YES | NO |
| *TMEM188* | 16q12.1 | 4.95E-04 | -2.01 | 50,059,189 | YES | ← | +/-LPS | +/-LPS | YES | NO |
| *USP2* | 11q23.3 | 4.39E-03 | -2.29 | 119,234,892 | YES | ↔ | -LPS | +/-LPS | YES | NO |
|  |  |  |  | 119,252,436 | YES | - | -LPS | +/-LPS | YES | NO |
| *ZNF627* | 19p13.2 | 1.39E-03 | -2.00 | 1,1708,235 | YES | → | +/-LPS | +/-LPS | YES | NO |
| *ZNF557* | 19q13.2 | 1.12E-02 | -2.04 | 7,069,471 | YES | - | +/-LPS | +LPS | YES | NO |
| *ZNF828* | 13q34 | 1.39E-04 | -2.02 | 115,079,965 | YES | → | +/-LPS | +/-LPS | YES | NO |
