# Supplementary material for: Combined Chromatin and Expression Analysis Reveals Specific Regulatory Mechanisms within Cytokine Genes in the Macrophage Early Immune Response
Source: PLoS One. 2012 Feb 27;7(2):e32306. doi: 10.1371/journal.pone.0032306 (PMC3288078; doi:10.1371/journal.pone.0032306)
Supplement: Table S7 — MatchInspector in silico TATA Box finding. Tables show the results of in silico TATA box finding in the subset of up- and down-regulated genes upon LPS stimulation in a distance of −150 bp/+50 bp of RefSeq gene annotated TSS. Gene symbol, Position of the TATA box from 5′UTR in base pairs (bp), gene strand direction (+, forward; −, reverse), promoter sequence (Lowercase DNA sequence letters: promoter area (Upstream 5′UTR), Uppercase DNA sequence: 5′UTR; and Underlined and in bold: TATA box sequence), TATA box category (VTATA.01: gTATAAAa, VTATA.02: ctATAAAA), and matrix similarity (cut off ≥0.9). (MatchInspector, Genomatix). (DOCX) [file pone.0032306.s010.docx]

**Table S7. MatchInspector *in silico* TATA Box finding (Genomatix)**

Cut off matrix similarity: ≥ 0.9

Underlined & in bold: TATA box sequence

Lowercase DNA sequence letters: Promoter area (Upstream 5´UTR)

Uppercase DNA sequence: 5´UTR

**1. Chemokines**

| **Gene** | **Position from 5’UTR** | **Gene Strand** | **Promoter Sequence** | **TATA BOX** | **Matrix similarity** |
| --- | --- | --- | --- | --- | --- |
| *CCL3* | -12bp | - | cc**tataaa**gaggagAGC | VTATA.01 | 0.955 |
| *CCL4* | -29bp | + | cc**tataaa**gagagattcccaagtcagtatcAGC | VTATA.01 | 0.949 |
| *CCL5* | -18bp | - | cc**tataaa**gggccagcctgaGCT | VTATA.01 | 0.958 |
| *CCL7* | -30bp | + | cc**tataaa**aggcagagacagagcttccagaggAGC | VTATA.01 | 0.999 |
| *CCL13* | -3bp | + | cc**tatAAA**AGGCCGGC | VTATA.01 | 0.999 |
| *CCL15* | -89bp | - | ca**tataaa**accccaatccaaaggtcaaaccaggcacttgaatctctcc  agtcacctgcttggcacttttccaagtgttctttactttcattCCT | VTATA.01 | 0.969 |
| *CCL18* | -43bp | + | cc**tataaa**aaggagagacaacagctcataccccagaaggaggccAGG | VTATA.01 | 0.996 |
| *CCL20* | -30bp | + | gc**tataaa**tagggccatcccaggctgctgtcAGA | VTATA.01 | 0.972 |
| *CCL23* | -19bp | - | ct**tataaa**tacaagggcagaaCTG | VTATA.01 | 0.95 |
| *CCL25 (L)* | -33bp | + | tc**tataaa**gagtgaagctcagcgtgttggtcctgcAGA | VTATA.01 | 0.943 |
| *CCR1* | +1bp | - | tc**ATAAAA**ACCCAGAAAG | VTATA.02 | 0.90 |
| *CXCL1* | -31bp | + | gc**ataaaa**ggggttcgcggatctcggagagcCAC | VTATA.02 | 0.918 |
| *CXCL8=IL8* | -20bp | + | ga**tataaa**aagccaccggagcaCTC | VTATA.01 | 0.987 |
| *CXCL11* | -52bp | - | cc**ataaaa**ggattgctggtgtataaaatgctctatatatgccaattatcaatTTC | VTATA.02 | 0.956 |
| *CXCL11* | -32 & -19bp | - | cc**ataaa**aggattgctggtg**tataaa**atgctctatatatgccaattatcaatTTC | VTATA.01 | 0.981/0.92 |
| *CXCR4 (L)* | -24bp | - | tt**tataaa**agtccggccgcggccagaAAC | VTATA.01 | 0.971 |
| *XCL1* | -44bp | + | gc**ataaaa**gaggtcctcaaagagcccgatcctcactctccttgcacAGC | VTATA.02 | 0.914 |
| *XCL2* | -31bp | + | gc**ataaa**agagatcctcaaagagcccgatcctcACT | VTATA0.2 | 0.914 |
| *CX3CL1* | **-**42bp | **+** | **attataaaaagccacagatctctggcggcggcaaggggacagcaCTG** | VTATA0.1 | 0.983 |

**2. Interleukins & Receptors**

| **Gene** | **Position from 5’UTR** | **Strand** | **Promoter Sequence** | **TATA BOX** | **Matrix similarity** |
| --- | --- | --- | --- | --- | --- |
| *IL1A* | -32bp | - | at**tataaa**agctgagaaattctttaataatagtaACC | VTATA.01 | 0.967 |
| *IL1B* | -32bp | - | cc**ataaaaa**cagcgagggagaaactggcagatACC | VTATA.02 | 0.942 |
| *IL1F6* | -46bp | + | ac**ataaaa**ggactcctatccttggcagttctgaaacaacaccaccaca | VTATA.02 | 0.901 |
| *IL1F10* | -30bp | + | gg**tataaa**cacagcgcaatgccctggagaaatcAGT | VTATA.01 | 0.939 |
| *IL2* | -24bp | - | ag**tataaa**ttgcatctcttgttcaagAGT | VTATA.01 | 0.958 |
| *IL8* | -20bp | + | ga**tataaaaagc**caccggagcaCTC | VTATA.01 | 0.987 |
| *IL9* | -129bp | - | ag**tataaa**gtggccccaacttacagagaaaaagtgggctcttggtatca  gtttgatgtcagggtttttccgtgtttgagagggagctttaaataccact  cgatttgaaggtgtctgcaagcgagctccagtCCG | VTATA.01 | 0.938 |
| *IL10* | -30bp | - | aa**tataaa**agggggacagagaggtgaaggtctACA | VTATA.01 | 0.985 |
| *IL11* | -46bp | - | ta**tataa**ccccccaggcgtccacactccctcactgccgcggccctgctGCT | VTATA.01 | 0.928 |
| *IL13* | -31bp | + | cc**tataaaa**gctgccacaagacgccaaggccacAAG | VTATA.02 | 0.997 |
| *IL16 (S)* | +17bp | + | TGACCCAAACATCCCCG**ATAAAA**CACCCACTGCTTAAGAGGCAGGCTCGG | VTATA.02 | 0.921 |
| *IL16 (M2)* | -43bp | + | ct**tataaa**aaatcagcctaaactctcccccgtgctttaaaaaactTGC | VTATA.01 | 0.958 |
| *IL16(M3)* | -26bp | + | ct**ttaaaa**ttatcatcctcattttacagATG | VTATA.02 | 0.910 |
| *IL17A* | -42bp | + | cc**tataaaa**agagagacgatagcgctacattttgtccatctcataGCA | VTATA.01 | 0.991 |
| *IL17F(L)* | -29 bp | - | gc**tataaaa**ctaacaggtactctcattgataGAA | VTATA.02 | 0.979 |
| *IL20(S)* | -34bp | + | cc**ataaaa**tccagacaatttccccctaggtgttttcGAT | VTATA.02 | 0.942 |
| *IL22* | -30bp | - | gc**tataaaa**gcagcagcttctaccttccccgtCAC | VTATA.02 | 0.996 |
| *Il23A* | -58bp | + | gg**tataa**agggcgggccttacaatgcagggaccttaaaagactcagagacaaagggagaAAA | VTATA.01 | 0.958 |
| *IL24* | -29bp | + | at**atata**tgcccaaatctccacaaagccttgCTT | VTATA.01 | 0.910 |
| *IL26* | -109 | - | ct**atataaa**ctgtaaattatctttcacagcacctcattggactggtggctttgagggc  tacattaagtaggttatcctctcatctaaccggcattagtatctcttgggctgCT | VTATA.01 | 0.931 |
| *IL32(M1)* | -51bp | + | cc**ataaa**accagctgagtatttgtgccaggaagactgcgtgcagaaggtgactGTC | VTATA.02 | 0.941 |
| *IL32(M2)* | +1bp | + | cc**ATAAAA**CCAG | VTATA.02 | 0.941 |
| *IL1R1(S)* | -137bp | + | tt**tataaat**tacccagtctcagatatttctttatagcagcgtgaaaatagactaa  tacatgcctcttgagatcctcacagcaattggtgcctttagacctcttcattcac  acttcactcatgtgttcttccttccccagGTA | VTATA.01 | 0.93 |
| *IL1R2(L)* | -141bp | + | ac**tttaaaa**ccacctctcggctggaagtacgtaatttttagcgagtcacagaaaaa  tagggaaacttatgcggcgtttccttggccacttccccatctgggtgatcatgtact  cagacccagcactgcagcctgggggtgctcCCC | VTATA.02 | 0.908 |
| *IL1RL1 (S)* | -28bp | + | ct**ttaaaa**tagaagagagtgagtagtctatGAG | VTATA.02 | 0.910 |
| *IL1RL1 (L)* | +33bp | + | ttAAAGAGAGGCTGGCTGTTGTATTTAGTAAAGC**TATAAA**GC | VTATA.01 | 0.926 |
| *IL7R* | -28bp | + | ag**tataaag**ccctagatctaagcttctctGTC | VTATA.01 | 0.939 |
| *IL17RB* | -10bp | + | gg**ataaaa**gcgcAGC | VTATA.01 | 0.902 |
| *IL18R1(S)* | -122bp | + | at**tataaa**aatcttctaggttgtttttttaaaaatctgtgtg  ccagaagatttttaaaccttcataagataggcacacttttgttt  gaaagctttggctgaatctgttttattctgttttccagAGA | VTATA.01 | 0.971 |
| *IL18R1(L)* | +31bp | + | AAAGAGAGGCTGGCTGTTGTATTTAGTAAAG**CTATAAA**GCTGT | VTATA.01 | 0.926 |
| *IL23R(M2)* | +10bp | + | GAATTACAAA**TATAAA**CTGCT | VTATA.01 | 0.918 |
| *IL31RA(L)* | -70bp | + | tc**ataaaa**ggcaaaaaattgcaaaaaaaaatagtaataaccagcatggca  ctaaatagaccatgaaaagacaTGT | VTATA.02 | 0.911 |

**3. Interferons**

| **Gene** | **Position from 5’UTR** | **Strand** | **Promoter Sequence** | **TATA BOX** | **Matrix similarity** |
| --- | --- | --- | --- | --- | --- |
| *IFNA8* | -60bp | + | ct**atataa**ggcggtgtacaaaccaaagtcttcagagaccca  ggttaagggtcatccatctgaACC | VTATA0.1 | 0.947 |
| *IFNB1* | -29bp | - | ca**tataaa**taggccatacccatggagaaaggACA | VTATA0.1 | 0.961 |
| *IFNE* | +28bp | - | CTTAGATATTAAACTGATAGGATAAGA**TATAAA**ATAATTTAAGATTGCTG | VTATA0.1 | 0.950 |
| *IFNK* | -55bp | + | gt**atata**aaggcacatgaaggaaaactcaaaacatcattgtcatatacaca  tcttctGGA | VTATA0.1 | 0.945 |
| *IFNW1* | -65bp | - | ct**ctaaaa**aacaaacaaacaacagcaaaatgctgttcttggttctaaagag  cttatttgctgcagatGAT | VTATA0.2 | 0.9 |
| *IFNG* | -30bp | - | gg**tataaa**taccagcagccagaggaggtgcagCAC | VTATA0.1 | 0.974 |

**4. Up-Regulated Genes**

| **Gene** | **Position from 5’UTR** | **Strand** | **Promoter Sequence** | **TATA BOX** | **Matrix similarity** |
| --- | --- | --- | --- | --- | --- |
| *ACSL1 (M3)* | +26bp | - | GGGACTGTGTTAAGAACCAAGGGCA**TATAAA**GA | VTATA.01 | 0.936 |
| *ATP2B1 (S)* | -65bp | - | ct**ttaaaa**tgttaatagtgatatgtgcctttaaagtatagtgaagttctatt  tgctcttaaatgtagCAA | VTATA.02 | 0.913 |
| *BCL2A1* | +35bp | - | AGCCTACGCACGAAAGTGACTAGGAGGAAGGATAT**TATAAA**GTGATGCAA | VTATA.01 | 0.916 |
| *CD69* | -30bp | - | ag**tataaa**ctctgagatgcctcagagcctcacAGA | VTATA.01 | 0.914 |
| *CTGF* | -26bp | - | cg**tataaaa**gcctcgggccgcccgccccAAA | VTATA.01 | 0.998 |
| *DLL4* | -40bp |  | at**atata**aggaaggcccccagcgcgcaggtttcagtagcggcGCT | VTATA.01 | 0.928 |
| *DUSP1(L)* | -25bp | - | ca**tataaa**cgcgctccccgggccaggcTCG | VTATA.01 | 0.932 |
| *EREG* | -30bp | + | ag**tataaa**gttcgcagcaccagacagttgagcTCA | VTATA.01 | 0.928 |
| *GCH1* | -30bp | - | cc**ataaaa**aggaggcgcggccgggctttccagCCT | VTATA.02 | 0.942 |
| *JUNB* | -41bp | + | gg**tataaa**ggcgtgtggctcaggctgagcggctgggaccttgaGAG | VTATA.01 | 0.954 |
| *KCNN2 (S)* | -47bp | + | ga**ataaa**agcaggctactggagccagcagtggcaacccgctcgggtcctCTT | VTATA.02 | 0.901 |
| *KYNU* | -28bp | + | tt**tataaa**ttctccactgggtggagtctgaGCA | VTATA.01 | 0.925 |
| *LIF* | +20bp | - | TACAACACAGGCTCCAGTAT**ATAAA**TCA | VTATA.01 | 0.933 |
| *LONRF1 (M)* | -19bp | - | ct**tataaa**atatttatttcagAAC | VTATA.01 | 0.939 |
| *MAP3K8* | -4bp | + | ca**tataAA**ATCCTGT | VTATA.01 | 0.967 |
| *NAV3 (S)* | -47bp | + | tt**tataaa**tttttctacataaagtttttctgtaatatttgtctttatagCTG | VTATA.01 | 0.915 |
| *NFKBIZ (S)* | -53bp | + | cg**ctataaagggc**gccagtgagaggggctggcctcctcttgccacgaggtcagacGGC | VTATA.01 | 0.957 |
| *PLEK* | -142bp | + | ct**ttaaa**agagggattttctgtatggtagtaagaatttcccttaggtaaa  tattcaacccaactgtggttttaataagtctccagcattagcaaggaatg  ggtggtttactctgcagttccttattctgatcttccaacccagtGAG | VTATA.02 | 0.927 |
| *PPP1R15A* | -1bp | + | c**tATAAA**AGCCTAGTGGCC | VTATA.01 | 0.997 |
| *PTGS2* | -28bp | - | ct**tataaaaagg**aaggttctctcggttagcGAC | VTATA.01 | 0.98 |
| *RASGEF1B* | +3bp | - | ttcAG**TATAA**ACAAGGAACC | VTATA.01 | 0.935 |
| *RNF144B* | -44bp | + | tg**tataaa**taaacgcgctgctaccgctgctggcgagctgtgccccaCGC | VTATA.01 | 0.957 |
| *SERPINB2 (s)* | -29bp | + | at**atataaa**gaattccttctttcttttcaagGCA | VTATA.01 | 0.928 |
| *SERPINB2 (L)* | -30bp | + | tg**tataaa**accagtcattaccatgtctgaactGTA | VTATA.01 | 0.973 |
| *SERPINB9 (L)* | -27bp | - | g**tataaa**agcggtgcctgcagaggccctaGCG | VTATA.01 | 0.996 |
| *SLC43A2* | -26bp | - | ga**ataaa**agggctggaataaaagagggcAGA | VTATA.01 | 0.904 |
| *STAT5A* | -30bp | + | agta**tataaa**agccgccgcatttcctgcccttgcATT | VTATA.01 | 0.986 |
| *TNF* | -37bp | + | ca**tataaa**ggcagttgttggcacacccagccagcagacg | VTATA.01 | 0.936 |
| *TNFSF9* | -4bp | + | gc**tatAAAA**AGCGGCGCG | VTATA.01 | 0.998 |
| *TWIST1* | -75bp | - | cc**tataaaa**cttcgaaaagtccctcctcctcacgtcaggccaatgac  actgctgcccccaaactttccgcctgcacgGAG | VTATA.02 | 0.980 |
| *TXNDC11* | +36bp | - | GGGCCGGGCCTTCGGGCCCGAGGCGGCGGCGGCGG**TATAAA**GCCGGCGAC | VTATA.01 | 0.937 |
| *ZBTB10(L)* | -145bp | + | ag**tataaa**cacgactggagttatctgattggctgctggtgatgtaacacc  catgtcaatacttcccagtgaacgcacgtgttcgaccttttatttaaatt  tccatttcatcacagcaataagacactcggggcggtgcattttctgtGAA | VTATA.01 | 0.944 |
| *ZC3H12A (L)* | +36bp | + | AGTGCCTCACCACCACCTGCCCGCGCGCCGCCCCAT**ATAAA**GGCGCCGCC | VTATA.01 | 0.942 |

**5. Down-Regulated Genes**

| **Gene** | **Position from 5’UTR** | **Strand** | **Promoter Sequence** | **TATA BOX** | **Matrix similarity** |
| --- | --- | --- | --- | --- | --- |
| *CCR1* | +1bp | - | tc**ATAAAA**ACCCAGAAAG | VTATA.02 | 0.90 |
| *CEBPA* | -40bp | - | g**tataaa**agctgggccggcgcgggccgggccattcgcgaccCGG | VTATA.01 | 0.984 |
| *GPR65* | -134bp | + | ct**ttaaaa**gtatttaagagactttttttgttcaaacttggataacgcaca  cccttctcattgagatgctgcttcctcattggtttgtgctgaaatatgag  tgcaatttcctgtcccctcagcagtgttggtttctcTTC | VTATA.02 | 0.927 |
| *HERPUD1* | -124bp | + | tt**tataaa**gagtacaaaagtgaatatatttcctttggaaattttcattg  Tgattagttggataggaaaagatcagagtttttatcaagtgatctttaaa  gaattttttttttttaaaaatggtctcGCT | VTATA.01 | 0.927 |
| *P2RY5(s)* | +35bp | - | AATGCTATGTTAAGATATCACTAACAATTTCAATT**TATAAA**TACA | VTATA.01 | 0.95 |
| *P2RY5(m)* | -24bp | - | aa**tataaa**cttcctcaggactcaccaGAA | VTATA.01 | 0.905 |
| *P2RY5(L)* | -31 & -8bp | - | tc**ataaaa**atcccaatgctgacatc**ataaaa**acTGG | VTATA.02 (X2) | 0.897 |

**Genomatix Sequences Logo used in the *in silico* analysis :**

**Cellular and viral TATA box elements (VTATA.01)**

- Bucher P

Weight matrix descriptions of four eukaryotic RNA polymerase II promoter elements derived from 502 unrelated promoter sequences.

J Mol Biol 212, 563-78 (1990)

**Mammalian C-type LTR TATA box (VTATA.02)**

- Golemis EA, Speck NA, Hopkins N

Alignment of U3 region sequences of mammalian type C viruses: identification of highly conserved motifs and implications for enhancer design. J Virol 64, 534-42 (1990)

- Frech K, Danescu-Mayer J, Werner T

A novel method to develop highly specific models for regulatory units detects a new LTR in GenBank which contains a functional promoter. J Mol Biol 270, 674-87 (1997)
